# Correction: Spatial patterns and predictors of antenatal care interruption in war-torn Tigray, northern Ethiopia: Spatial modelling approach

**DOI:** 10.1371/journal.pone.0336494

**Published:** 2025-11-07

**Authors:** Assefa Ayalew Gebrselassie, Mussie Alemayehu, Haftu Gebrehiwot, Brhane Ayele, Hailay Gebretnsae, Ferehiwot Hailemariam, Tsegay Wellay, Adhena Ayalew, Araya Abrha Medhanyie, Znabu Hadush Kahsay, Liya Mamo, Mebrahtu Kalayu Chekole, Reda Shamie, Mohammedtahir Yahya, Melaku Abraha, Hayelom Kahsay, Tsegay Hadgu, Fana Gebreslassie, Asfawosen Aregay, Kiros Demoz, Mulugeta Tilahun, Mulugeta Woldu, Ataklti Gebrtsadik, Abraham Aregay Desta, Gebrehaweria Gebrekuristos, Amanuel Haile, Rieye Esayas, Tsegay Berihu, Abrham Gebrelibanos, Tadele Tesfean, Ashenafi Asmelash, Afework Mulugeta Bezabh

The captions for Figs 1 and 2 are incorrectly switched. Please see the complete, correct Figs 1 and 2 caption here.

**Fig 1:**
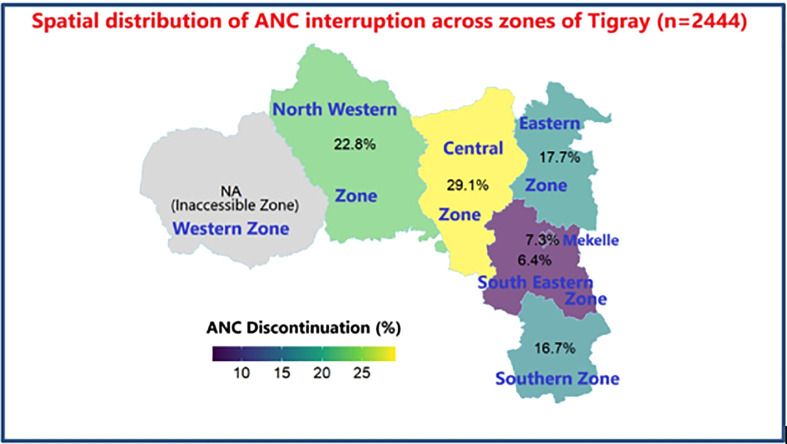
Special distribution of ANC interruption across the zone of Tigray Region (n = 2444).

**Fig 2:**
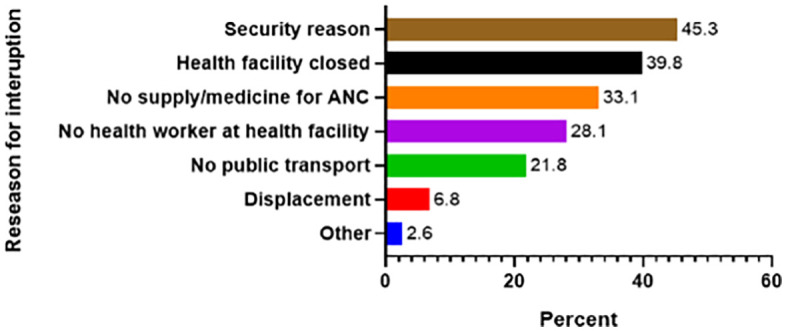
Reasons for interruption of ANC care during the war from Pretoria November 2020 to August 2022 agreement of the Tigray region, Northern Ethiopia.
