# Supplementary material for: Spatial patterns and predictors of antenatal care interruption in war-torn Tigray, northern Ethiopia: Spatial modelling approach
Source: PLoS One. 2025 Aug 14;20(8):e0328802. doi: 10.1371/journal.pone.0328802 (PMC12352755; doi:10.1371/journal.pone.0328802)
Supplement: Attachment — Submitted filename: PONE-D-25-08979_reviewer_Commented.docx [file pone.0328802.s001.docx]

PLOS ONE

Spatial patterns and predictors of antenatal care interruption in war-torn Tigray, northern Ethiopia: spatial modeling approach

--Manuscript Draft--

| Manuscript Number: | PONE-D-25-08979 |
| --- | --- |
| Article Type: | Research Article |
| Full Title: | Spatial patterns and predictors of antenatal care interruption in war-torn Tigray, northern Ethiopia: spatial modelling approach |
| Short Title: | Spatial patterns and predictors of antenatal care interruption |
| Corresponding Author: | Assefa Ayalew Gebreslassie, PhD student Mekelle University College of Health Sciences Mekelle, Tigray ETHIOPIA |
| Keywords: | ANC; interruption; spatial logistic; war; Tigray |
| Abstract: | Introduction  Antenatal care (ANC) is crucial for maternal health, yet its provision has been severely disrupted in conflict-affected Tigray, Ethiopia. This study uses a community-based approach to analyse the spatial patterns and predictors of ANC interruption.  Materials &Methods  A multistage cross-sectional design was employed to select 2,444 eligible women from 13,915 women of reproductive age across from six zones and 57 clusters. Data were collected through structured questionnaires, and geographic coordinates were obtained for spatial geo-points. A spatial logistic regression model assessed the effects of geographic and socio-demographic factors on ANC interruptions, accounting for spatial dependencies.  Results  In this study, the prevalence of ANC interruption was 24.8% (n=605, 95% CI:23.1%, 26.5%). Each additional minute of travel increased the log-odds of interruption by 0.000353 (SE = 0.000103, p < 0.001), highlighting accessibility challenges due to security checkpoints and conflict-related dangers. Geographic zone significantly affected ANC interruptions, with women in the southeast and eastern zones experiencing lower log-odds compared to the northwest zone. Additionally, having more children under five raised the log-odds of interruption by 0.083134 (SE = 0.017295, p < 0.001). Households without a radio had a 0.08939 lower log-odds of interruption (SE = 0.033425, p = 0.007489), indicating the importance of access to information.  Conclusions  The findings reveal significant ANC interruptions in Tigray, influenced by geographic disparities, household dynamics, and information access. Improving infrastructure and security, implementing targeted interventions, enhancing mass media health information dissemination, and further research on geographic disparities are essential. |
| Order of Authors: | Assefa Ayalew Gebreslassie, PhD student |
|  | Mussie Alemayehu |
|  | Haftu Gebrehiwot |
|  | Brhane Ayele |
|  | Hailay Gebretnsae |
|  | Ferehiwot Hailemariam |
|  |  |

|  | Tsegay Wellay |
| --- | --- |
|  | Adhena Ayalew |
|  | Araya Abrha Medhanyie |
|  | Znabu Hadush Kahsay |
|  | Liya Esayas Mamo |
|  | Mebrahtu Kalayu Chekole |
|  | Reda Kalayu Shamie |
|  | Mohammedtahir Mulugeta Yahya |
|  | Melaku Mulugeta Abraha |
|  | Hayelom Kalayu Kahsay |
|  | Tsegay Hadgu |
|  | Fana Gebreslassie |
|  | Asfawosen Aregay |
|  | Kiros Demoz |
|  | Mulugeta Tilahun |
|  | Mulugeta Woldu |
|  | Ataklti Gebrtsadik |
|  | Abraham Aregay Desta |
|  | Gebrehaweria Gebrekurstos |
|  | Amanuel Mulugeta Haile |
|  | Rieye Esayas |
|  | Tsegay Esayas Berihu |
|  | Abrham Gebrelibanos |
|  | Tadele Tesfean |
|  | Ashenafi Asmelash |
|  | Afework Mulugeta Bezabh |
| Additional Information: |  |
| Question | Response |
| Financial Disclosure  Enter a financial disclosure statement that describes the sources of funding for the work included in this submission. Review the [submission guidelines](http://journals.plos.org/plosone/s/submission-guidelines#loc-financial-disclosure-statement) for detailed requirements. View published research articles from [*PLOS ONE*](https://journals.plos.org/plosone/) for specific examples.  This statement is required for submission and will appear in the published article if the submission is accepted. Please make sure it is accurate. | Yes |

| Funded studies  Enter a statement with the following details:   - Initials of the authors who received each award - Grant numbers awarded to each author - The full name of each funder - URL of each funder website - Did the sponsors or funders play any role in the study design, data collection and analysis, decision to publish, or preparation of the manuscript? |  |
| --- | --- |
| Did you receive funding for this work? |  |
| Please add funding details.  as follow-up to "Financial Disclosure  Enter a financial disclosure statement that describes the sources of funding for the work included in this submission. Review the [submission guidelines](http://journals.plos.org/plosone/s/submission-guidelines#loc-financial-disclosure-statement) for detailed requirements. View published research articles from [*PLOS ONE*](https://journals.plos.org/plosone/) for specific examples.  This statement is required for submission and will appear in the published article if the submission is accepted. Please make sure it is accurate. | UNICEF, WHO, Amref Health Africa and UNFPA and Tigray regional health bureau and Mums for Mums (MfM) for all-rounded support in ensuring a successful and productive integrated research data collection process, we assure there is no any competing programming and any other support that influences the research output |
| Funded studies  Enter a statement with the following details:   - Initials of the authors who received each award - Grant numbers awarded to each author - The full name of each funder - URL of each funder website - Did the sponsors or funders play any role in the study design, data collection and analysis, decision to publish, or preparation of the manuscript? |  |
| Did you receive funding for this work?" |  |
| Please select the country of your main research funder (please select carefully as in some cases this is used in fee | ETHIOPIA - ET |

| calculation).  as follow-up to "Financial Disclosure  Enter a financial disclosure statement that describes the sources of funding for the work included in this submission. Review the [submission guidelines](http://journals.plos.org/plosone/s/submission-guidelines#loc-financial-disclosure-statement) for detailed requirements. View published research articles from [*PLOS ONE*](https://journals.plos.org/plosone/) for specific examples.  This statement is required for submission and will appear in the published article if the submission is accepted. Please make sure it is accurate. |  |
| --- | --- |
| Funded studies  Enter a statement with the following details:   - Initials of the authors who received each award - Grant numbers awarded to each author - The full name of each funder - URL of each funder website - Did the sponsors or funders play any role in the study design, data collection and analysis, decision to publish, or preparation of the manuscript? |  |
| Did you receive funding for this work?" |  |
| Competing Interests  Use the instructions below to enter a competing interest statement for this submission. On behalf of all authors, disclose any [competing interests](http://journals.plos.org/plosone/s/competing-interests) that could be perceived to bias this work—acknowledging all financial support and any other relevant financial or non- financial competing interests.  This statement is required for submission and will appear in the published article if the submission is accepted. Please make sure it is accurate and that any funding sources listed in your Funding Information later in the submission form are also declared in your Financial Disclosure | I have read the journal's policy and the authors of this manuscript have no any competing interest |

| statement.  View published research articles from  [*PLOS ONE*](http://journals.plos.org/plosone/) for specific examples. |  |
| --- | --- |
| NO authors have competing interests  Enter: *The authors have declared that no competing interests exist.*  Authors with competing interests  Enter competing interest details beginning with this statement:  *I have read the journal's policy and the authors of this manuscript have the following competing interests: [insert competing interests here]* |  |
| * typeset |  |
| Ethics Statement  Enter an ethics statement for this submission. This statement is required if the study involved:   - Human participants - Human specimens or tissue - Vertebrate animals or cephalopods - Vertebrate embryos or tissues - Field research   Write "N/A" if the submission does not require an ethics statement.  General guidance is provided below. Consult the [submission guidelines](http://journals.plos.org/plosone/s/submission-guidelines#loc-guidelines-for-specific-study-types) for detailed instructions. Make sure that all information entered here is included in the Methods section of the manuscript. | N/A |

| Format for specific study types  Human Subject Research (involving human participants and/or tissue)   - Give the name of the institutional review board or ethics committee that approved the study - Include the approval number and/or a statement indicating approval of this research - Indicate the form of consent obtained (written/oral) or the reason that consent was not obtained (e.g. the data were analyzed anonymously)   Animal Research (involving vertebrate animals, embryos or tissues)   - Provide the name of the Institutional Animal Care and Use Committee (IACUC) or other relevant ethics board that reviewed the study protocol, and indicate whether they approved this research or granted a formal waiver of ethical approval - Include an approval number if one was obtained - If the study involved *non-human primates*, add *additional details* about animal welfare and steps taken to ameliorate suffering - If anesthesia, euthanasia, or any kind of animal sacrifice is part of the study, include briefly which substances and/or methods were applied   Field Research  Include the following details if this study involves the collection of plant, animal, or other materials from a natural setting:   - Field permit number - Name of the institution or relevant body that granted permission |  |
| --- | --- |
|  |  |
| Data Availability  Authors are required to make all data underlying the findings described fully available, without restriction, and from the time of publication. PLOS allows rare exceptions to address legal and ethical concerns. See the [PLOS Data Policy](http://journals.plos.org/plosone/s/data-availability) and [FAQ](http://journals.plos.org/plosone/s/data-availability#loc-faqs-for-data-policy) for detailed information. | Yes - all data are fully available without restriction. |

| A Data Availability Statement describing where the data can be found is required at submission. Your answers to this question constitute the Data Availability Statement and will be published in the article, if accepted. |  |
| --- | --- |
| Important: Stating ‘data available on request from the author’ is not sufficient. If your data are only available upon request, select ‘No’ for the first question and explain your exceptional situation in the text box. |  |
| Do the authors confirm that all data underlying the findings described in their manuscript are fully available without restriction? |  |
| Describe where the data may be found in full sentences. If you are copying our sample text, replace any instances of XXX with the appropriate details.   - If the data are held or will be held in a public repository, include URLs, accession numbers or DOIs. If this information will only be available after acceptance, indicate this by ticking the box below. For example: *All XXX files are available from the XXX database (accession number(s) XXX, XXX.*). - If the data are all contained within the manuscript and/or Supporting Information files, enter the following: *All relevant data are within the manuscript and its Supporting Information files.* - If neither of these applies but you are able to provide details of access elsewhere, with or without limitations, please do so. For example:   *Data cannot be shared publicly because of [XXX]. Data are available from the XXX Institutional Data Access / Ethics Committee (contact via XXX) for researchers who meet the criteria for access to confidential data.*  *The data underlying the results presented in the study are available from (include the name of the third party* | Data is available within the manuscript. |

| *and contact information or URL).*   - This text is appropriate if the data are owned by a third party and authors do not have permission to share the data.   * typeset |  |
| --- | --- |
| Additional data availability information: |  |

<https://www2.cloud.editorialmanager.com/pone/download.aspx?id=39405924&guid=f805c2be-3512-4bfb-a0c5-cea95f7472f7&scheme=1>Manuscript	[Click here to access/download;Manuscript;Manuscript.docx](https://www2.cloud.editorialmanager.com/pone/download.aspx?id=39405924&guid=f805c2be-3512-4bfb-a0c5-cea95f7472f7&scheme=1)

1 ***Spatial patterns and predictors of antenatal care interruption***
2 ***in war-torn Tigray, northern Ethiopia: spatial modelling***
3 ***approach***

4

5 Assefa Ayalew Gebrselassie *^1^ ,Mussie Alemayehu^1,4^, Haftu Gebrehiwot^4^ , Brhane Ayele^4^, Hailay
6 Gebretnsae^4^ ,Ferehiwot Hailemariam^1^, Tsegay Wellay^1^, Adhena Ayalew^1^, Araya Abrha
7 Medhanyie^1,6^, Znabu Hadush Kahsay^1^, Liya Mamo^1^, Mebrahtu Kalayu Chekole^1^, Reda Shamie^1^,
8 Mohammedtahir Yahya^2,3^ , Melaku Abraha^3^, Hayelom Kahsay^4^, Tsegay Hadgu^4^, Fana
9 Gebreslassie^4^, Asfawosen Aregay^4^ , Kiros Demoz ^4^ , Mulugeta Tilahun^4^, Mulugeta Woldu^4^, Ataklti
10 Gebrtsadik^4^, Abraham Aregay Desta^4^, Gebrehaweria Gebrekuristos^5^, Amanuel Haile^5^, Rieye
11 Esayas^5^,Tsegay Berihu^5^,Abrham Gebrelibanos^6^, Tadele Tesfean^6^, Ashenafi Asmelash^7^ , Afework
12 Mulugeta Bezabh^1^

13

14 ^1^ School of Public Health, Mekelle University, College of Health Sciences, Mekelle, Ethiopia.
15 ^2^ Department of Obstetrics and Gynecology, School of Medicine, Mekelle University, College of
16 Health Sciences, Mekelle, Ethiopia.
17 ^3^ Mekelle Hamlin Fistula Center, Mekelle, Tigray Ethiopia
18 ^4^ Tigray Health Research Institute, Mekelle, Tigray Ethiopia
19 ^5^ Tigray Health Bureau, Mekelle, Tigray Ethiopia
20 ^6^ MARCH research center, College health Sciences, Mekelle, Tigray, Ethiopia
21 ^7^ Mums for Mums, Mekelle, Tigray, Ethiopia

22

23 *Corresponding author
24 Email: [aayalew120@gmail.com](mailto:aayalew120@gmail.com) (AA)

25

26

27

28

# 29 Abstract

30 **Introduction**
31 Antenatal care (ANC) is crucial for maternal health, yet its provision has been severely disrupted
32 in conflict-affected Tigray, Ethiopia. This study analyzes the spatial patterns and predictors of
33 ANC interruption using a community-based approach.

# 34 Materials &Methods

35 A multistage cross-sectional design was employed to select 2,444 eligible women from 13,915
36 women of reproductive age across from six zones and 57 clusters. Data were collected through
37 structured questionnaires, and geographic coordinates were obtained for spatial geo-points. A
38 spatial logistic regression model assessed the effects of geographic and socio-demographic factors
39 on ANC interruptions, accounting for spatial dependencies.

# 40 Results

41 In this study, the prevalence of ANC interruption was 24.8% (n=605, 95% CI:23.1%, 26.5%). Each
42 additional minutes of travel increased the log-odds of interruption by 0.000353 (SE = 0.000103, p
43 < 0.001), highlighting accessibility challenges due to security checkpoints and conflict-related
44 dangers. Geographic zone significantly affected ANC interruptions, with women in the southeast
45 and eastern zones experiencing lower log-odds compared to the northwest zone. Additionally,
46 having more children under five raised the log-odds of interruption by 0.083134 (SE = 0.017295,
47 p < 0.001). Households without a radio had a 0.08939 lower log-odds of interruption (SE =
48 0.033425, p = 0.007489), indicating the importance of access to information.

# 49 Conclusions

50 The findings reveal significant ANC interruptions in Tigray, influenced by geographic disparities,
51 household dynamics, and information access. Improving infrastructure and security, implementing
52 targeted interventions, enhancing mass media health information dissemination, and further
53 research on geographic disparities are essential.
54 **Key words**
55 ANC, interruption, spatial logistic, war, Tigray

# 56 Introduction

57 Antenatal care (ANC) is essential for the health of pregnant women and their fetuses, aiding in
58 labor preparation and the recognition of pregnancy-related warning signs (1). ANC interruption is
59 defined as not receiving at least 8 visits, according to new guidelines (2). In conflict-affected
60 populations, women and children face disproportionate morbidity (3).
61 From 2000 to 2017, global maternal mortality ratios (MMR) decreased by 38%, from 342 to 211
62 deaths per 100,000 live births (5). However, African nations in conflict or post-conflict situations
63 still report high maternal mortality rates. For example, in Tigray, the MMR rose drastically from
64 186 per 100,000 live births in 2019 to 840 in 2023 due to the war (6, 7, 8, 9, 10).
65 While the global average for ANC coverage exceeds 75%, many conflict-affected regions fall
66 below this standard. In Somalia, for instance, ANC coverage is only 9.4%, and Afghanistan reports
67 a skilled birth attendant (SBA) coverage of 17.8% (11, 12, 13, 14). In Tigray, maternal ANC
68 service utilisation plummeted from 90% before the conflict to 50% during the war (15, 16).
69 Understanding the spatial patterns of ANC interruption—identifying specific areas within Tigray
70 with varying interruption rates—and the predictors of these interruptions, such as conflict impact,
71 and socioeconomic factors remain limited. Therefore, assessing the magnitude
72 percentage of ANC interruptions and their barriers is crucial for developing effective maternal health
73 interventions in Tigray post-conflict.

# 74 Materials and methods

75 **Study Setting, design, data source**
76 This cross-sectional study utilized survey data from integrated surveys aimed at evaluating cost-
77 effectiveness in post-conflict settings in Tigray, Ethiopia. Conducted by Mekelle University, the
78 Tigray Health Research Institute, and the Tigray Regional Health Bureau, the research involved
79 13,915 female participants, of whom 10,654 were of reproductive age (15-49 years), and 3,933
80 had been pregnant since the onset of the conflict. The analysis focused on 2,444 women who were
81 pregnant women during the conflict had received antenatal care (ANC). Data collection occurred
82 a month, from July 1 to July 30, 2023[17]. This systematic approach allowed for a comprehensive
83 examination of factors affecting interruption in ANC within this vulnerable population
84 **Statistical Method and Analysis**
85 Univariate descriptive, bivariate chi-square and multivariate spatial logistic regression approaches
86 were employed to analyze the ANC data from targeted zones of the Tigray Region. The univariate
87 analysis were made use of to summarize & describe the demographic variables (e.g., age, gender, etc),
88 using frequencies, percentages, means, and standard deviations provided a detailed
89 overview. Associations between categorical variables (e.g., gender, zone, etc) and ANC
90 interruptions were assessed using bivariate Chi-square or Fisher’s exact tests. To model the
91 relationship between risk factors and cholera occurrence across zones, we used ANC interruption.
92 Spatial logistic regression is an advanced modeling approach that incorporates zones, representing
93 administrative or geographic divisions, to analyze binary outcomes while accounting for spatial
94 dependencies. The model extends ordinary logistic regression by including spatial random effects
95 (∅𝐳) to capture unobserved spatial variation between zones, improving the accuracy of estimates
96 and inference. The model is expressed as:

𝑷_𝒊_

97	𝐥𝐨𝐠𝐢𝐭(𝑷 |)) = 𝐥𝐧 {	} = 𝛃

𝒑

+ ∑ 𝛃 𝑿

+ ∅ ,	𝐳 = 𝐒, 𝐒𝐄, 𝐌, 𝐄, 𝐂, 𝐍𝐖

^𝒊^	𝟏 − 𝑷_𝒊_)

𝐨

𝒌=𝟏

𝐤 𝒊𝒌	𝐳

98 where 𝑷_𝒊_ represents the probability of the outcome for observation 𝒊, 𝛃_𝐨_ is the intercept, 𝑿𝒊𝒌 are
99 predictors, 𝛃𝐤 are their coefficients, and ∅𝐳 captures the spatial effect for zone 𝐳 . This approach
100 allows the model to account for spatial clustering and patterns, addressing biases and inaccuracies
101 that arise from spatial autocorrelation in ordinary logistic regression. Spatial autocorrelation in the
102 dependent variables were assessed using Moran’s I test. The spatial logistic regression model
103 incorporated structured spatial random effects, is modelled through a Conditional Autoregressive
104 (CAR) framework, to account for spatial dependencies between zones. This makes it valuable
105 tools for policymakers and researchers aiming to address spatial inequalities in various fields,
106 , including public health, urban development, and environmental management
107 Spatial logistic regression provides several advantages in analyzing binary outcomes, particularly
108 by incorporating spatial dependencies that traditional models often overlook. This approach
109 enhances model fitness and predictive accuracy, facilitating the identification of high-risk areas
110 through spatial clustering. It also uncovers spatial patterns and clusters, which can inform targeted
111 public health interventions [18,19,20]. The analysis was conducted using R version 4.4.1, with
112 Weighing applied to adjust for non-proportional sampling. Both exploratory and confirmatory
113 analyses were carried out, including descriptive statistics, visualisations, and inferential statistics.
114 Predictors with p < 0.05 in the bivariate and multivariate analysis were incorporated into the final
115 spatial logistic regression model.

# 116 Results

117 **Sociodemographic characteristics of participants**
118 The study participants are divided into two groups: hosts 2,069 (84.7%, 95% CI: [83.5%, 85.9%])
119 and internally displaced people 375 (15.3%, 95% CI: [14.1%, 16.5%]). The mean age of
120 participants is 28 years, with a standard deviation of 0.05.
121 A significant portion of the participants have not completed formal education. Specifically, 835
122 individuals (34.2%, 95% CI: [32.5%, 36.0%]) are illiterate, while 754 (30.9%, 95% CI: [29.2%,
123 32.6%] completed elementary school. Additionally, 540 participants (22.1%, 95% CI: [20.7%,
124 23.5%]) completed secondary school, and only 201 individuals (8.2%, 95% CI: [7.3%, 9.1%])
125 hold a college degree or higher.
126 Geographically, most participants reside in rural areas, with 1,530 (62.6%, 95% CI: [60.9%,
127 64.3%]) living in such regions. In contrast, 190 participants (7.8%, 95% CI: [6.5%, 9.1%]) live in
128 semi-urban areas, while 724 (29.6%, 95% CI: [27.9%, 31.3%]) inhabit urban locations. A large
129 majority, 2,239 participants (91.6%, 95% CI: [90.5%, 92.7%]), are married.
130 Access to resources is limited among the participants. For instance, 1,615 individuals (66.1%, 95%
131 CI: [64.4%, 67.8%]) lack electricity, and 1,373 (56.2%, 95% CI: [54.5%, 57.9%]) do not have
132 access to public transportation. Media ownership is also low, with only 308 participants (12.6%,
133 95% CI: [11.3%, 13.9%]) owning a radio. In contrast, mobile phone ownership is more common,
134 with 1,138 individuals (46.6%, 95% CI: [44.9%, 48.3%]) possessing a mobile phone. Television
135 ownership is relatively low, as just 400 participants (16.4%, 95% CI: [15.1%, 17.7%]) have a
136 television or detail see “**Table 1**.”

137

138 Table 1: Socio-demographic of pregnant women following ANC during the war **(N 2444)**

| Variables |  | Response | Freq | % |
| --- | --- | --- | --- | --- |
|  |  | South | 346 | 14.2 |
|  |  | South-east | 233 | 9.5 |
| Zone |  | Mekelle  Eastern | 308  520 | 12.6  21.3 |
|  |  | Central | 624 | 25.5 |
|  |  | Northwest | 413 | 16.9 |
| Participant |  | Host  IDP* | 2069  375 | 84.7  15.3 |
| Religion |  | Orthodox  Muslim | 2369  75 | 96.9  3.1 |
|  |  | Rural | 1530 | 62.6 |
| Residence |  | Semi-urban | 190 | 7.8 |
|  |  | Urban | 724 | 29.6 |
| Highest educational | level | Unable to read and | 835 | 34.2 |
| attained |  | write |  |  |

|  | Able to read and write | 114 | 4.7 |
| --- | --- | --- | --- |
|  | Elementary school | 754 | 30.9 |
|  | Secondary school | 540 | 22.1 |
|  | College and above | 201 | 8.2 |
|  | Single | 41 | 1.7 |
|  | Married | 2239 | 91.6 |
|  | Divorced | 127 | 5.2 |
|  | Widowed | 11 | 0.5 |
| Marital status | Separated | 26 | 1.1 |
| Access to electricity | Yes  No | 829  1615 | 33.9  66.1 |
| Radio ownership | Yes  No | 308  2136 | 12.6  87.4 |
| Television ownership | Yes  No | 400  2044 | 16.4  83.6 |
|  | Yes | 1138 | 46.6 |
| Mobile phone ownership | No | 1306 | 53.4 |
| Have been displaced | Yes  No | 1386  1058 | 56.7  43.3 |
|  | Yes | 1421 | 58.1 |
| Does the war impact your life | No | 1023 | 41.9 |
| Time taken for the double trip to | < 1 hour | 1583 | 64.8 |
| health post | >=1 hour | 861 | 35.2 |

139 ***IDP-internally displaced people**

140

141 The prevalence of antenatal care (ANC) interruption was found to be 24.8% (95% CI: [23.1%,
142 26.5%]), indicating that nearly one in four women encountered difficulties in maintaining
143 consistent ANC. The primary reasons for these interruptions included insecurity (45.3%, 95% CI:
144 [43.1%, 47.5%]), health facility closures (39.8%, 95% CI: [37.6%, 42.0%]), and a lack of supplies
145 and medicine for ANC (33.1%, 95% CI: [31.0%, 35.2%]) for additional details **(Fig 1.)**

146

147 **Fig 1. Special distribution of ANC interruption across the zone of Tigray Region (n=2444)**
148 **(Fig2.) Reasons for interruption of ANC care during the war from Pretoria November 2020 to August**
149 **2022 agreement of the Tigray region, Northern Ethiopia**
150 **Bivariate Analysis: Impact of War on Antenatal Care (ANC) Visits**
151 The study found that 75.2% (1,839, 95% CI: [73.9%, 76.5%]) of participants did not postpone or
152 interrupted their antenatal care (ANC) visits due to the ongoing war, while 24.8% (605, 95% CI:
153 [23.5%, 26.1%]) reported interruptions. Among those with a sick family member in the last two
154 weeks, 14.3% (349, 95% CI: [12.9%, 15.7%]) did not postpone their ANC visit, and 7.0% (171,
155 95% CI: [5.9%, 8.1%]) did.
156 The war's impact was significant, with 96.5% (2,358, 95% CI: [95.4%, 97.6%]) reporting a family
157 Member were disabled; of these, 72.7% (1,778, 95% CI: [71.0%, 74.4%]) did not postpone their ANC
158 visit, while 23.7% (580, 95% CI: [22.2%, 25.2%]) did. Additionally, 97.0% (2,370, 95% CI:
159 [96.0%, 98.0%]) experienced the loss of a family member, with 73.2% (1,790, 95% CI: [71.5%,
160 74.9%]) continuing their ANC visits. A small fraction, 1.4% (34, 95% CI: [0.8%, 2.0%]), reported
161 a woman in their household experiencing rape due to the war, with only 1.1% (27, 95% CI: [0.5%,
162 1.7%]) not postponing ANC visits.
163 Property damage was reported by 53.3% (757, 95% CI: [51.0%, 55.6%]) of participants, with
164 38.2% (543, 95% CI: [35.9%, 40.5%]) not postponing their ANC visits. Looting affected 89.7%
165 (1,274, 95% CI: [88.5%, 90.9%]), with 66.1% (939, 95% CI: [64.5%, 67.7%]) continuing them
166 visits. Destruction of property was noted by 27.3% (388, 95% CI: [25.6%, 29.0%]) of participants,
167 where 20.3% (288, 95% CI: [18.7%, 21.9%]) did not postpone. Displacement impacted 56.7%
168 (1,386, 95% CI: [54.9%, 58.5%]), with 42.0% (1,027, 95% CI: [40.3%, 43.7%]) not postponing
169 their ANC visits.
170 In terms of distance to health posts, 64.8% (1,583, 95% CI: [63.0%, 66.6%]) reported a round trip
171 of less than 1 hour, while 35.2% (861, 95% CI: [33.4%, 37.0%]) reported longer trips. Those who
172 did not postpone their ANC visits were more likely to live closer to health facilities (49.7% vs.
173 15.1%).
174 Functionality of nearby health facilities was high, with 75.8% (1,303, 95% CI: [74.1%, 77.5%])
175 reporting nearby health posts as functional and 97.0% (2,371, 95% CI: [96.0%, 98.0%]) for health
176 centers/hospitals. Access to public transport was reported by 43.8% (1,071, 95% CI: [41.9%,
177 45.7%]), with those who had not postponed ANC visits slightly more likely to have access (32.8%
178 vs. 11.0%).
179 Regarding electricity supply, 33.9% (829, 95% CI: [32.1%, 35.7%]) reported having it, and those
180 who did not postpone their ANC visits were somewhat more likely to have electricity (26.5% vs.
181 9.0%). Media ownership was limited, with 12.6% (308, 95% CI: [11.3%, 13.9%]) owning a radio
182 and 16.4% (400, 95% CI: [15.1%, 17.7%]) owning a television. Those who did not postpone their
183 ANC visits were slightly more likely to own a radio (9.6% vs. 3.5%) or a TV (12.6% vs. 4.5%).
184 Mobile phone ownership was similar between both groups, with 46.6% (1,138, 95% CI: [44.9%,
185 48.3%]) owning a phone

186

187

188 **Table 2: War and ANC interruption of ANC care during the war to Pretoria November 2020 to August 2022 agreement of the Tigray region**
189 **Northen Ethiopia(N=2444)**

**Predictors	Categories**

**ANC Interruption due to**

**P-**

**war	χ^2^**

**Predictors	Categories**

**ANC Interruption due to**

**P-**

**war	χ^2^**

**value	val**

**No(*n=1839*)	Yes(*n=605*)	No(*n=1839*)	Yes(*n=605*)**

**Sick	Last	2**

| **Weeks** | No | 1490  (61.0%) | 434  (17.8%) | **23.44** | **<0.001** | **HP Distance** | >= 1 hour | 625(25.6%) | 236(9.7%) | **4.3** | **4.3** |
| --- | --- | --- | --- | --- | --- | --- | --- | --- | --- | --- | --- |
|  | Yes | 61(2.5%) | 25(1.0%) |  |  |  | Yes | 994(57.8%) | 309(18.0%) |  |  |
| **Disabled War** |  |  |  | **11.38** | **11.38** | **Functional HP** |  |  |  | **11.38** | **11.** |
|  | No | 1778(72.7%) | 580(23.7%) |  |  |  | No | 249(14.5%) | 93(5.4%) |  |  |
|  |  |  |  |  |  |  | Didnt |  |  |  |  |
| **Lost Family** | Yes | 49(2.0%) | 25(1.0%) | **11.38** | **11.38** | **Functional HC** | know | 56(3.3%) | 19(1.1%) | **11.38** | **11.** |
| **member in War** |  |  |  |  |  |  |  |  |  |  |  |
|  | No | 1790(73.2%) | 580(23.7%) |  |  |  | No | 18 (24.0%) | 57 (76.0%) |  |  |
|  | Yes | 27(1.1%) | 7(0.3%) | **11.38** | **11.38** |  | Yes | 19 (17.4%) | 90 (82.6%) | **11.38** | **11.** |

Yes	349 (14.3%)	171 (7%)

<= 1 hour	1214(49.7%)	369(15.1%)

**Rape	victim**

**Access	to**

326

| **HHs** | No | **transport** | | | | | **to** | No | 153 (31.9%) | | | |
| --- | --- | --- | --- | --- | --- | --- | --- | --- | --- | --- | --- | --- |
|  |  | 1812(74.1%) | 598(24.5%) |  |  | **HP/HC** | |  |  | (68.1%) |  |  |
| **War Damage** | Yes  No | 490(34.5%)  543(38.2%) | 174(12.2%)  214(15.1%) | 11.38 | 11.38 | **Electricity**  **Supply** | | Yes  No | 24 (23.5%)  18 (24.0%) | 78 (76.5%)  57 (76.0%) | 11.38 | 11. |
| **War Looted** | Yes  No | 94(6.6%)  939(66.1%) | 53(3.7%)  335(23.6%) | 11.38 | 11.38 | **Radio in the HH** | | Yes  No | 24 (23.5%)  18 (24.0%) | 78 (76.5%)  57 (76.0%) | 11.38 | 11. |
| **Displaced due to**  **War** | Yes  No | 1027(42.0%)  812(33.2%) | 359(14.7%)  246(10.1%) | 11.38 | 11.38 | **TV in HH** | | Yes  No | 24 (23.5%)  18 (24.0%) | 78 (76.5%)  57 (76.0%) | 11.38 | 11. |
| **War Impact in**  **HH** | Yes  No | 1033(42.3%)  806(33.0%) | 388(15.9%)  217(8.9%) | 11.38 | 11.38 | **Mobile**  **Ownership** | | Yes  No | 24 (23.5%)  18 (24.0%) | 78 (76.5%)  57 (76.0%) | 11.38 | 11. |
| 190 |  |  |  |  |  |  | |  |  |  |  |  |

191 **Sexual and reproductive health characteristics of participants**
192 The study participants reported varying experiences regarding pregnancies and births. The
193 majority had 2 pregnancies (21.7%), followed by 1 pregnancy (20.3%). The number of pregnancies
194 decreases with higher counts: 3 pregnancies (17.2%), 5 (10.4%), 6 (6.5%), 7 (4.7%), and 8 (5.2%).
195 In terms of births, most participants reported 1 birth (22.7%) or 2 births (22.6%), with subsequent
196 numbers decreasing: 3 births (16.6%), 4 (11.9%), 5 (9.0%), 6 (5.7%), 7 (3.8%), and 8 (3.7%).
197 Additionally, 4.1% have not given birth.
198 Regarding live births, the distribution is as follows: 1 live birth (23.2%), 2 (22.2%), 3 (16.6%), 4
199 (11.9%), 5 (8.5%), 6 (5.6%), 7 (3.5%), and 8 or more (3.2%). Notably, 5.3% of participants
200 reported having no live births.
201 The majority, 2,328 (95.0%), reported 0 stillbirths, while 95 (3.9%) had 1 stillbirth, 18 (0.7%) had
202 2, 2 (0.08%) had 3, and 1 (0.04%) had 4 stillbirths.

203 Regarding abortions, 1,622 (66.4%) reported having 0 abortions in their lifetime. However, a
204 notable portion has experienced abortions: 698 (28.6%) reported 1 abortion, 100 (4.1%) reported
205 2, 18 (0.7%) reported 3, 5 (0.2%) reported 4, and 1 (0.04%) reported 5 **(Fig 3)**.
206 **Fig 3. The line graph shows the reproductive history of study participants of the interruption of ANC**
207 **during the war in Tigray 2023.**
208 Pregnancy intentions among participants
209 The study revealed varied pregnancy intentions among participants. A majority, 1,455 (59.5%),
210 reported actively trying to become pregnant at the time of conception. However, a significant
211 portion, 738 (30.2%), expressed a desire to wait until later to have children. Additionally, 251
212 (10.3%) stated that they did not wish to have any more children when they became pregnant. The
213 Findings of this study indicate a diverse range of pregnancy intentions within the study population, highlighting
214 that while many participants were intentionally seeking pregnancy, a notable number preferred to
215 delay or avoid further pregnancies. For more details, see “Table 2”.

216

217 **Multivariate Analysis: Parameter Estimates from SLR**
218 The Moran’s I test indicated significant spatial autocorrelation in ANC interruption (Moran’s I =
219 0.546, p-value < 0.001), indicating the presence of spatial autocorrelation in the dependent
220 variable, validating the need for spatial logistic regression. Incorporating spatial dependencies is
221 crucial, as neglecting them in ordinary logistic regression may result in biased parameter estimates
222 and inaccurate inferences.
223 The spatial logistic regression model identified several key factors influencing the interruption of
224 antenatal care (ANC) during the war. For every unit increase in distance from the health center,
225 the log-odds of interrupting ANC increased by 0.000353 (SE = 0.000103, p < 0.001). Geographic
226 differences also played a significant role; women living in the south-east zone had a 0.11876 lower
227 log-odds of interrupting ANC compared to those in the north-west zone (SE = 0.050501, p =
228 0.018685). Additionally, women in the eastern zone exhibited a 0.16452 lower log-odds of
229 interrupting ANC compared to the north-west zone (SE = 0.048012, p = 0.000611).
230 The number of children under five in the household was another important factor, with each
231 additional child increasing the log-odds of interrupting ANC by 0.083134 (SE = 0.017295, p <
232 0.001). Furthermore, households without a radio had a 0.08939 lower log-odds of interrupting
233 ANC compared to those with a radio (SE = 0.033425, p = 0.007489). These results suggest that
234 longer distances to health centers, residing in the north-west zone, having more young children,
235 and not owning a radio were all associated with a greater likelihood of ANC interruption in the
236 war-affected Tigray region of Ethiopia. In contrast, the analysis for Mekelle indicated an estimate
237 of -0.04, a standard error of 0.07, and a z-value of -0.55, resulting in a p-value greater than 0.05,
238 which shows no significant effect on ANC interruption compared to the north-west zone.
239 Conversely, the eastern zone had an estimate of -0.16, a standard error of 0.05, and a z-value of -
240 3.43, with a p-value less than 0.05, indicating significantly lower odds of ANC interruption and
241 better adherence in this zone for detail see “Table 3”
242 Table 3: Spatial logistic regression model analysis, shows the parameter estimates, standard errors,
243 z-values, and p-values for the predictors of interruption of ANC during the war in Tigray 2023.

| Predictors | Estimate | Std. Error | Z-value | Pr(>\|z\|) |
| --- | --- | --- | --- | --- |
| (Intercept) | 0.164124 | 0.152572 | 1.0757 | 0.282055 |
| South-east zone | -0.11876 | 0.050501 | -2.3518 | 0.018685 |
| Mekelle zone | -0.0377 | 0.068806 | -0.5479 | 0.583761 |
| Eastern zone | -0.16452 | 0.048012 | -3.4266 | 0.000611 |
| Central zone | 0.038805 | 0.041975 | 0.9245 | 0.35524 |
| Northwest zone | 0.035293 | 0.046268 | 0.7628 | 0.445589 |
| Age | 0.000458 | 0.002425 | 0.189 | 0.85008 |
| Number under five children | 0.083134 | 0.017295 | 4.8068 | 1.53E-06 |
| Number of Pregnancies | 0.002781 | 0.008414 | 0.3305 | 0.741027 |
| Family size | 0.002114 | 0.007472 | 0.2829 | 0.77725 |
| Perceived wealth medium | 0.08881 | 0.128912 | 0.6889 | 0.490878 |
| Perceived wealth poor | 0.060797 | 0.130133 | 0.4672 | 0.640364 |
| Radio ownership | -0.08939 | 0.033425 | -2.6743 | 0.007489 |
| Television ownership | -0.02766 | 0.051554 | -0.5365 | 0.591633 |
| Mobile phone ownership | -0.04237 | 0.023887 | -1.7739 | 0.076077 |

|  | Perceived food Security moderately |  | | | |
| --- | --- | --- | --- | --- | --- |
|  | secured |  |  |  |  |
|  |  | 0.03115936 | 0.047596 | 0.6547 | 0.512689 |
|  | Perceived food Security insecure | 0.035233 | 0.051334 | 0.6863 | 0.492493 |
|  | War impacted me | -0.04285 | 0.022561 | -1.8993 | 0.05752 |
|  | Distance health post in minutes | -0.00044 | 0.000266 | -1.6706 | 0.094804 |
|  | Distance of health center in minutes | 0.000353 | 0.000103 | 3.415 | 0.000638 |
|  | Access to transport health Center | -0.00869 | 0.029711 | -0.2925 | 0.769874 |
| 244 |  |  |  |  |  |

# 245 Discussion

246 The prevalence of antenatal care (ANC) interruption was found to be 605 (24.8%), indicating that
247 nearly one in four women faced challenges in maintaining consistent ANC. This is acritical
248 concern since ANC is a well-established and cost-effective intervention that provides essential
249 care for pregnant women from early pregnancy to delivery [21]. The interruption was notably more
250 severe among mothers residing in rural areas (370, 61.2%) compared to semi-urban (51, 8.4%)
251 and urban (184, 30.4%) settings. A significant majority of those affected were host residents (519,
252 85.8%) rather than internally displaced mothers (86, 14.2%). Additionally, a large portion of
253 participants were married (563, 91.1%).
254 Similar studies conducted in Syria have found that violent events significantly reduce the
255 likelihood of pregnant women seeking and accessing essential healthcare services related to
256 maternal health. The fear and risks associated with ongoing conflicts deter pregnant women from
257 seeking necessary care, increasing the vulnerability of both mothers and their unborn children to
258 potential health complications [22]. Research in Yemen highlighted those pregnant women in
259 remote, rural areas encountered substantial barriers to reaching ANC clinics due to infrastructure
260 damage, lack of public transportation, and financial constraints [23]. Furthermore, studies in
261 Afghanistan have shown that the Taliban's restrictions on women's movement and access to
262 healthcare during conflicts severely limited pregnant women's ability to attend ANC appointments
263 [24,25]. These challenges were particularly pronounced in rural areas compared to urban settings
264 [26].
265 This situation contrasts with the internal resolution of the World Health Organisation (WHO
266 2012), which emphasizes the need for leadership in documenting evidence of attacks against health
267 workers, facilities, and patients in areas affected by armed conflict [27].
268 The results of the spatial logistic regression analysis in the war-affected Tigray region of Ethiopia
269 revealed several key factors influencing ANC interruptions. For instance, the distance to health
270 centers was a significant factor; for every unit increase in distance (in minutes), the log-odds of
271 discontinuing ANC increased by 0.000353 (SE = 0.000103, p < 0.001). This finding aligns with
272 studies conducted in Syria, where ongoing conflict and insecurity significantly disrupted access to
273 ANC services. Women reported facing barriers such as transportation difficulties, fear of
274 checkpoints, and financial constraints when trying to reach health facilities [28,29]. Additionally,
275 a study in Syria found that women living in besieged or hard-to-reach areas experienced much
276 greater difficulty accessing ANC services due to transportation challenges, security concerns, and
277 a lack of functioning health facilities [30].
278 Living in the north-west zone of the war-affected Tigray region was associated with a higher
279 likelihood of discontinuing antenatal care (ANC). Additionally, households with a greater number
280 of young children were more prone to discontinuation, as were those that did not own a radio.
281 These findings underscore the unique challenges faced by pregnant women in war-affected
282 regions, highlighting the need for improved access to health facilities, addressing transportation
283 and security concerns, and implementing targeted interventions to support households with

multiple young children.

284 Research from Yemen and the Democratic Republic of the Congo further illustrates the severe
285 Impact of armed conflicts and civil wars on maternal healthcare access in sub-Saharan Africa. In
286 Yemen, the escalation of the civil war led to the closure of many health facilities, significantly
287 hindering pregnant women’s ability to receive regular ANC [31,32]. Similarly, findings from the
288 Democratic Republic of the Congo indicate that armed conflicts and displacement forced many
289 pregnant women to miss critical ANC visits, resulting in poorer maternal and child health
290 outcomes [33,34,35]. Pregnant women displaced to rural areas in the DRC exhibited much lower
291 ANC attendance rates compared to those who remained in or fled to urban centers [36]. Overall,
292 the effects of civil wars on sub-Saharan Africa have severely compromised maternal and child
293 health services [37].
294 The spatial logistic regression analysis highlighted distance to health centers as a significant factor;
295 for every unit increase in distance (in minutes), the log-odds of discontinuing ANC increased by
296 0.000353 (SE = 0.000103, p < 0.001). This finding aligns with a study in post-war Liberia, where
297 women in rural areas had low odds of attending at least four ANC visits and utilizing formal
298 institutional delivery services [38]. Additionally, studies from Yemen reported substantial barriers
299 for pregnant women in remote, rural areas trying to access ANC clinics, primarily due to
300 infrastructure damage, lack of public transportation, and financial constraints [39,40].
301 Geographic zones also played a critical role; women living in the south-east zone had a 0.11876
302 lower log-odds of discontinuing ANC compared to those in the north-west zone (SE = 0.050501,
303 p = 0.018685). Similarly, women in the eastern zone exhibited a 0.16452 lower log-odds of
304 discontinuing ANC compared to the north-west zone (SE = 0.048012, p = 0.000611). Thus, living
305 in the north-west zone was associated with a higher likelihood of ANC discontinuation in the
306 Tigray region. This finding mirrors those from the Eastern Mediterranean region, where conflicts
307 have disrupted health service utilization, especially in rural areas [41]. A study in the Democratic
308 Republic of the Congo noted that pregnant women displaced to rural areas had significantly lower
309 ANC attendance rates than those remaining in or fleeing to urban centers [42,43]. Furthermore,
310 research in Afghanistan showed that the Taliban's restrictions on women's movement
311 disproportionately affected rural women, leading to higher rates of missed ANC appointments
312 compared to their urban counterparts [44,45,46,47].
313 These findings underscore the critical importance of addressing the unique challenges faced by
314 pregnant women in war-affected regions, particularly those living in remote, rural areas with
315 limited access to health facilities. Enhancing transportation, infrastructure, and security in these
316 areas is essential for improving maternal healthcare utilization and outcomes.
317 Broader patterns in conflict-affected settings reveal that the uneven distribution of conflict
318 intensity and damage to health infrastructure across different zones has led to varying impacts on
319 pregnant women's ability to receive antenatal care (ANC). The findings from this study in Tigray
320 align with another analysis indicating that, as of June 2021, only 27.5% of hospitals, 17.5% of
321 health centers, 11% of ambulances, and none of the 712 health posts were functional [48].
322 While the specific reasons for the higher ANC interruption in the north-west zone of Tigray were
323 not detailed, further contextual analysis is needed to understand the underlying drivers of this
324 geographic disparity. Additionally, the number of children under five in the household
325 significantly influenced ANC continuity; for each additional child, the log-odds of discontinuing
326 ANC increased by 0.083134 (SE = 0.017295, p < 0.001). This trend may reflect the additional
327 caregiving responsibilities that pregnant mothers with multiple young children face, which can
328 hinder their ability to prioritize their own ANC visits.
329 Moreover, household radio ownership emerged as an important factor; households without a radio
330 had a 0.08939 lower log-odds of discontinuing ANC compared to those with a radio (SE =
331 0.033425, p = 0.007489). This suggests that access to media and information sources, such as
332 radio, can positively influence ANC utilization, as mothers who receive ANC-related information
333 are more likely to act on it.
334 The Tigray study offers a more granular, zone-level analysis that complements zonal-level findings
335 from other conflict-affected contexts, highlighting the importance of understanding subnational
336 variations in healthcare access during conflicts. Further research to unpack the underlying drivers
337 of geographic disparities in ANC interruption in Tigray would be invaluable for informing more
338 targeted interventions.
339 **Strengths and limitations of the study**

# 340 Conclusion and recommendations

341 The study identified a high prevalence of antenatal care (ANC) interruption, with nearly one in
342 four women (24.8%) facing challenges in maintaining consistent attendance. Key factors
343 associated with the likelihood of discontinuing ANC included greater distance from health centers,
344 residing in the north-west zone compared to the south-east and eastern zones, having a higher
345 number of children under five in the household, and household ownership of a radio.
346 To address these findings, several strategies are recommended. First, improving access to health
347 centers could be achieved by establishing more community-based ANC services or implementing
348 transportation assistance programs. Second, targeted interventions are needed for high-risk
349 north-west zone to specifically address the elevated rates of ANC interruption in this region. Third,
350 providing additional resources and support, such as childcare assistance or conditional cash
351 transfers could benefit households with multiple young children, helping to alleviate caregiving
352 burdens.
353 Promoting the use of radio and other mass media channels is also crucial for disseminating
354 information and encouraging ANC attendance, particularly in households with a radio.
355 Additionally, further research is essential to unpack the underlying drivers of geographic
356 disparities in ANC interruption in Tigray, which would inform more targeted and effective
357 interventions.

# 358 Ethical considerations

359 Ethical clearance was approved by the Tigray Health Research Institute review board with reference
360 number (THRI 4031/1099/15). A permission letter was obtained from the Tigray regional health bureau.
361 Oral consent and volunteer participation were obtained from study participants after fully
362 communicated the benefits and risks of participation in the study with a right to discontinue
363 participation at any stage of the interview. Data confidentiality was maintained throughout the
364 research process.

# 365 Acknowledgements

366 We extend our heartfelt gratitude to the Tigray Health Bureau (THB) for leading this integrated
367 research and mobilising technical staff for data collection. Lastly, we thank the participants, as
368 well as the data collectors and supervisors, particularly those from the Mekelle University College of
369 Health Sciences and Tigray Health Research Institute (THRI), for their commitment and integrity
370 in generating quality data.

# 371 Conflict of Interest

373 **Authors’ Contributions:**
374 A. A., M. A., H. G., and B. A., conceived and designed the experiments and analyzed and write
375 up the report H. G., F. H., T. W., A. A., A. A. M., Z. H. K., L. M., M. K. C., R. S., M. A., H. K.,
376 T. H., F. G., A. A., K. D., M. T., M. W., A. G., A. A. D., G. G., A. H., R. E., T. B., A. G., T. T.,
377 A. A., A. M. B. performed the experiments and approved and revised the final manuscript

# 378 Reference

379 1. WHO, UNICEF, UNFPA and TheWorld Bank. Trends in MaternalMortality: 2000 to

379	2017WHO. Geneva:WHO (2019).

380 2. Tunçalp Ö, Pena-Rosas JP, Lawrie T, et al. WHO Recommendations on antenatal care for a
381 positive pregnancy experience-going beyond survival. Bjog. 2017;124(6):860-862.
382 3. O’hare BAM, David PS. First do no harm: the impact of recent armed conflict on maternal and
383 child	health	in	Sub-Saharan	Africa.	J	R	Soc	Med.	(2007)	100:564–70.	doi:
384 10.1258/jrsm.100.12.564
385 4. DeJong J, Ghattas H, Bashour H, Mourtada R, Akiki C, Reese_MastersonA. Reproductive,
386 maternal, neonatal and child health in conflict: a case
387 study on Syria using countdown indicators. BMJ Global Health. (2017) 2:e000302. doi:
388 10.1136/bmjgh-2017-000302
389 5. Akseer N, Wright J, Tasic H, Everett K, Scudder E, Amsalu R, et al. Women, children and
390 adolescents in conflict countries: an assessment of
391 inequalities in intervention coverage and survival BMJ Global Health. (2020) 5:e002214. doi:
392 10.1136/bmjgh-2019-002214
393 6. Mullany LC, Lee CI, Yone L, Paw P, Oo EKS, Maung C, et al. Access to essential maternal
394 health interventions and human rights violations among vulnerable communities in Eastern
395 Burma. PLoS Med. (2008)5: e242. doi: 10.1371/journal.pmed.0050242
396 7. ETHIOPIA Demographic and Health Survey Central Statistical Agency Addis Ababa,
397 Ethiopia The DHS Program ICF Rockville, Maryland, USA, 2016
398 8.	Abay, S.T., Gebre-egziabher, A.G. Status and associated factors of birth registration in
399 selected districts of Tigray region, Ethiopia. *BMC Int Health Hum Rights* **20**, 20 (2020).
400 https://doi.org/10.1186/s12914-020-00235-x
401 9. Godefay H, Byass P, Graham WJ, Kinsman J, Mulugeta A. Risk factors for maternal mortality
402 in	rural	Tigray,	northern	Ethiopia:	a	case-control	study.	PloS	one.	2015	Dec
403 17;10(12):e0144975.
404 10. Legesse AY, Teka Tseghay H, Abraha HE, Fisseha G, Ebrahim MM, Tsadik M, Berhe B,
405 Gebrekurstos G, Ayele B, Gebremeskel T, Gebremariam T. Maternal mortality during war
406 time in Tigray, Ethiopia: A community‐based study. BJOG: An International Journal of
407 Obstetrics & Gynaecology. 2024 May;131(6):786-94.
408 11. Namasivayam, Amrita, González, Pedro Arcos, Delgado, Rafael Castro, Chi,Primus Che. The
409 effect of armed conflict on the utilization of maternal health services in Uganda: a population-
410 based	study.	PLoS	Curr	Disast.	(2017)	9:1689–98.	doi:

| 411 | 10.1371/currents.dis.557b987d6519d8c7c96f2006ed3c271a |
| --- | --- |
| 412 12. | Kabakian-Khasholian, Tamar, Shayboub, Rawan, El-Kak, Faysal. Seeking maternal care at |
| 413 | times of conflict: the case of Lebanon. Health Care Women Int. (2013) 34:352–62. doi: |
| 414 | 10.1080/07399332.2012.736570 |
| 415 13. | Kabakian-Khasholian, Tamar, Shayboub, Rawan, El-Kak, Faysal. Seeking maternal care at |
| 416 | times of conflict: the case of Lebanon. Health Care Women Int. (2013) 34:352–62. doi: |
| 417 | 10.1080/07399332.2012.736570 |
| 418 14. | Chi PC, Bulage P, Urdal H, Sundby J. Perceptions of the effects of armed conflict on maternal |
| 419 | and reproductive health services and outcomes inBurundi and Northern Uganda: a qualitative |
| 420 | study. BMC Int Health Hum Rights. (2015) 15:7. doi: 10.1186/s12914-015-0045-z |
| 421 15. | Indicators K. Mini demographic and health survey. EPHI and ICF. 2019 Jul. |
| 422 16. | : Gesesew H, Berhane K, Siraj ES, et al. The impact of war on the health system of the Tigray |
| 423 | region in Ethiopia: an assessment. BMJ Global Health 2021;6: e007328. doi:10.1136/ bmjgh- |
| 424 | 2021-007328 |
| 425 17. | Mussie Alemayehu etal, does integration of surveys bring cost effectiveness into evidence |
| 426 | generation in post conflict settings of Tigray, Ethiopia? East African journal of Health sciences |
| 427 | ORCID: 0000-0002-0035-631X <https://journal.mu.edu.et/index.php/eajhs/issue/view/56> |
| 428 18. | Paciorek CJ. Computational techniques for spatial logistic regression with large data sets. |
| 429 | Comput Stat Data Anal. 2007;51(8):3631-53. doi:10.1016/j.csda.2006.11.008. |

430 19. Baddeley A, Berman M, Fisher N, et al. Spatial logistic regression and change-of-support in
431 Poisson point processes. Electron J Stat. 2010;4:1151-201. doi:10.1214/10-ejs581.
432 20. Bihrmann K, Toft N, Nielsen SS, et al. Spatial correlation in Bayesian logistic regression with
433 misclassification.	Spatial	Spatio-temporal	Epidemiol.	2014;9:1-12.
434 doi:10.1016/j.sste.2014.02.002
435 21. World Health Organization. WHO recommendations on antenatal care for a positive pregnancy
436 experience: summary: highlights and key messages from the World Health Organization's
437 2016 global recommendations for routine antenatal care. World Health Organization; 2018.
438 22. Ekzayez A, Alhaj Ahmad Y, Alhaleb H, Checchi F. The impact of armed conflict on utilisation
439 of health services in north-west Syria: an observational study. Conflict and health. 2021 Dec;
440 15:1-0.
441 23. Stanikzai MH, Tawfiq E, Jafari M, Wasiq AW, Seddiq MK, Currie S, Sayam H, Baray AH,
442 Saeedzai SA. Contents of antenatal care services in Afghanistan: findings from the national
443 health survey 2018. BMC Public Health. 2023 Dec 11;23(1):2469.
444 24. Glass N, Jalalzai R, Spiegel P, Rubenstein L. The crisis of maternal and child health in
445 Afghanistan. Conflict and health. 2023 Jun 12;17(1):28.
446 25. Saleem SM, Shoib S, Dazhamyar AR, Chandradasa M. Afghanistan: decades of collective
447 trauma, ongoing humanitarian crises, Taliban rulers, and mental health of the displaced
448 population. Asian journal of psychiatry. 2021 Nov 1; 65:102854.
449 26. Zhang T, He Q, Richardson S, Tang K. Does armed conflict lead to lower prevalence of
450 maternal health-seeking behaviours: theoretical and empirical research based on 55 683
451 women in armed conflict settings. BMJ Global Health. 2023 Aug 1;8(8): e012023.
452 27. World Health Organization. WHO’s response, and role as the health cluster lead, in meeting
453 the growing demands of health in humanitarian emergencies [Internet]. Sixty-Fifth World
454 Health Assembly. 2012 May:21-6.
455 28. Mourtada R, Bashour H, Houben F. A qualitative study exploring barriers to adequate uptake
456 of antenatal care in pre-conflict Syria: low-cost interventions are needed to address disparities
457 in antenatal care. Contraception and Reproductive Medicine. 2021 Jun 1;6(1):17.
458 29. Basha S, Socarras A, Akhter MW, Hamze M, Albaik A, Hussein I, Tarakji A, Hamadeh M,
459 Loutfi R, Kewara M, Alahdab F. Protracted armed conflict and maternal health: a scoping
460 review of literature and a retrospective analysis of primary data from northwest Syria. BMJ
461 Global Health. 2022 Aug 1;7(8): e008001.
462 30. Nabulsi D, Abou Saad M, Ismail H, Doumit MA, El-Jamil F, Kobeissi L, Fouad FM. Minimum
463 initial service package (MISP) for sexual and reproductive health for women in a displacement
464 setting: a narrative review on the Syrian refugee crisis in Lebanon. Reproductive Health. 2021
465 Dec; 18:1-3.
466 31. Qirbi N, Ismail SA. Health system functionality in a low-income country in the midst of
467 conflict: the case of Yemen. Health policy and planning. 2017 Jul 1;32(6):911-22.
468 32. Hyzam D, Zou M, Boah M, Saeed A, Li C, Pan S, Zhai J, Wu LJ. Health information and
469 health-seeking behaviour in Yemen: perspectives of health leaders, midwives and mothers in
470 two rural areas of Yemen. BMC Pregnancy and Childbirth. 2020 Dec; 20:1-2.
471 33. Stamidis K, Bologna L, Losey L. CORE Group Polio Project (CGPP) Final Evaluation Report

472	2017.

473	34. Zhang T, Qi X, He Q, Hee J, Takesue R, Yan Y, Tang K. The effects of conflicts and self-

474	reported insecurity on maternal healthcare utilisation and children health outcomes in the

475	democratic republic of congo (Drc). InHealthcare 2021 Jul 3 (Vol. 9, No. 7, p. 842). MDPI.

| 476 | 35. Bwirire D, Roosen I, De Vries N, Letschert R, Ntabe Namegabe E, Crutzen R. Maternal Health |
| --- | --- |
| 477 | Care Service Utilization in the Post-Conflict Democratic Republic of Congo: An Analysis of |
| 478 | Health Inequalities over Time. InHealthcare 2023 Oct 31 (Vol. 11, No. 21, p. 2871). MDPI. |
| 479 | 36. Ormhaug CM. Effects of civil war on maternal and child health care in sub-Saharan Africa |
| 480 | 2014 |
| 481 | 37. Ziegler BR. *Pregnancy in Peril: the impact of conflict on antenatal care and skilled birth* |
| 482 | *attendant utilization in the Democratic Republic of the Congo and Burundi* (Doctoral |
| 483 | dissertation, The University of Western Ontario (Canada))2020. |
| 484 | 38. Yaya S, Uthman OA, Bishwajit G, Ekholuenetale M. Maternal health care service utilization |
| 485 | in post-war Liberia: analysis of nationally representative cross-sectional household surveys. |
| 486 | BMC Public Health. 2019 Dec;19:1-2 |
| 487 | 39. Grainger, C.G., Gorter, A.C., Al-Kobati, E. *et al.* Providing safe motherhood services to |
| 488 | underserved and neglected populations in Yemen: the case for vouchers. *Int J Humanitarian* |
| 489 | *Action* **2**, 6 (2017). <https://doi.org/10.1186/s41018-017-0021-4> |
| 490 | 40. Sheline A. The Yemen War in Numbers: Saudi Escalation and US Complicity. Quincy Institute |
| 491 | of US Complicity. 2022 Mar 21 |
| 492 | 41. GOVERNMENT ACCOUNTABILITY OFFICE WASHINGTON DC. Uncertain Political |
| 493 | and Security Situation Challenges US Efforts to Implement a Comprehensive Strategy in |
| 494 | Yemen. 2012 |
| 495 | 42. Karakır İA. Ongoing conflict in Yemen: A proxy war Tesam Akademi Dergisi. 2018;5(2):121- |
| 496 | 49. |
| 497 |  |

| 498 | 43. Krug EG, Mercy JA, Dahlberg LL, Zwi AB. The world report on violence and health. The |
| --- | --- |
| 499 | lancet. 2002 Oct 5;360(9339):1083-8. |
| 500 | 44. Alibhai KM, Ziegler BR, Meddings L, Batung E, Luginaah I. Factors impacting antenatal care |
| 501 | utilization: a systematic review of 37 fragile and conflict-affected situations. Confl Health. |
| 502 | 2022 Jun 11;16(1):33. doi: 10.1186/s13031-022-00459-9. PMID: 35690840; PMCID: |
| 503 | PMC9188725 |
| 504 | 45. Wake SK, Botore A, Mohammed A, Gemede K, Bariso M, Gerema U. Disparities in Antenatal |
| 505 | Care Visits between Urban and Rural Ethiopian Women. J Pregnancy. 2023 Sep 27; |
| 506 | 2023:9031344. doi: 10.1155/2023/9031344. PMID: 37799709; PMCID: PMC10550413. |
| 507 | 46. Ahmadi A. Assessing Participation in Women's Development Projects in Afghanistan 2017 |
| 508 | 47. Saqib M. *Afganistan’da Devlete Yapılanan Kusurlar: Afganistan’ın 2004 Anayasasına Göre* |
| 509 | *Yerel Seçimlerinin Kritik Bir Analizi* (Doctoral dissertation, Marmara Universitesi (Turkey)). |
| 510 | 2022 |
| 511 | 48. Gesesew H, Berhane K, Siraj ES, et al. The impact of war on the health system of the Tigray |
| 512 | region in Ethiopia: an assessment. BMJ Global Health 2021;6: e007328. doi:10.1136/ bmjgh- |
| 513 | 2021-007328 |

<https://www2.cloud.editorialmanager.com/pone/download.aspx?id=39405926&guid=244106ee-6122-4866-83af-1f214273eeaf&scheme=1>Figure 1	[Click here to access/download;Figure;Fig 1.tif](https://www2.cloud.editorialmanager.com/pone/download.aspx?id=39405926&guid=244106ee-6122-4866-83af-1f214273eeaf&scheme=1)

<https://www2.cloud.editorialmanager.com/pone/download.aspx?id=39405928&guid=1dff0b3c-3add-4a67-a1ed-194596502873&scheme=1>Figure 2	[Click here to access/download;Figure;Fig 2.tif](https://www2.cloud.editorialmanager.com/pone/download.aspx?id=39405928&guid=1dff0b3c-3add-4a67-a1ed-194596502873&scheme=1)

<https://www2.cloud.editorialmanager.com/pone/download.aspx?id=39405929&guid=e374e14f-1767-4964-b79f-606e93f15d6d&scheme=1>Figure 3	[Click here to access/download;Figure;Fig 3.tif](https://www2.cloud.editorialmanager.com/pone/download.aspx?id=39405929&guid=e374e14f-1767-4964-b79f-606e93f15d6d&scheme=1)
