# Supplementary material for: Spatial patterns and predictors of antenatal care interruption in war-torn Tigray, northern Ethiopia: Spatial modelling approach
Source: PLoS One. 2025 Aug 14;20(8):e0328802. doi: 10.1371/journal.pone.0328802 (PMC12352755; doi:10.1371/journal.pone.0328802)
Supplement: Attachment — Submitted filename: Response to Reviewers.docx [file pone.0328802.s002.docx]

**Response to editor and Reviewers**

PLOS ONE

Thank you for the opportunity to revise our manuscript titled "Spatial Patterns and Predictors of Antenatal Care Interruption in War-Torn Tigray, Northern Ethiopia: Spatial Modeling Approach Manuscript ID: PONE-D-25-08979. We appreciate the constructive feedback from you and the reviewers. Below, we address each comment in detail.

2. We note that the grant information you provided in the ‘Funding Information’ and ‘Financial Disclosure’ sections do not match.

When you resubmit, please ensure that you provide the correct grant numbers for the awards you received for your study in the ‘Funding Information’ section.

**Response:** our funding had not grant number because the budget was utilizing through perdiem .

. Thank you for stating the following financial disclosure:

 [UNICEF, WHO, Amref Health Africa and UNFPA and Tigray regional heal bureau   and Mums for Mums (MfM) for all rounded support in ensuring a successful and productive integrated research data collection process we assure there is no any competing programming and any other support that influences the research output]. The funders had no role in study design, data collection and analysis, decision to publish, or preparation of the manuscript

Please state what role the funders took in the study.  If the funders had no role, please state:

**Response thank you for your comment we have revised as you suggested**

 UNICEF, WHO, Amref Health Africa and UNFPA and Tigray regional heal bureau   and Mums for Mums (MfM) for all rounded support in ensuring a successful and productive integrated research data collection process we assure there is no any competing programming and any other support that influences the research output. The funders had no role in study design, data collection and analysis, decision to publish, or preparation of the manuscript

**In** the research on antenatal care interruption in Tigray, we maintain a strong commitment to avoiding conflicts of interest. We ensure that our funding sources do not create biases, as we do not have any conflicts with organizations that might have vested interests in the outcomes, such as government agencies UNICEF, WHO, Amref Health Africa and UNFPA and Tigray regional heal bureau   and Mums for Mums (MfM with specific agendas. Our collaborative relationships with local health authorities and NGOs are structured to prevent any conflicts, as these entities do not have a stake in the research findings that could influence our work. Additionally, the researchers do not have personal ties to the region or its healthcare systems that could compromise the interpretation of data or reporting results. We are also vigilant against publication bias, ensuring that we do not face conflicts that would pressure us to publish only positive outcomes aligned with funding interests, which could distort the integrity of our research. Ethical considerations are paramount; we adhere to rigorous ethical guidelines to address the unique challenges of working with vulnerable populations in conflict zones, recognizing the dilemmas that may arise. To mitigate potential conflicts, we prioritize transparency by disclosing all funding sources and potential conflicts in our publications. We are committed to an independent review process, welcoming input from independent experts in study design and data analysis to ensure objectivity. Finally, we uphold ethical oversight by obtaining approval from relevant review boards Tigray health research institute (THRI) as mentioned above to safeguard participant welfare, reinforcing our dedication to conducting responsible and ethical research

4. Thank you for stating the following in the Acknowledgments Section of your manuscript:

[We extend our heartfelt gratitude to the Tigray Health Bureau (THB) for leading this integrated research and mobilizing technical staff for data collection. Lastly, we thank the participants, as well as the data collectors and supervisors, particularly those from Mekelle University College of Health Sciences and Tigray Health Research Institute (THRI), for their commitment and integrity in generating quality data.]

We note that you have provided funding information that is not currently declared in your Funding Statement. However, funding information should not appear in the Acknowledgments section or other areas of your manuscript. We will only publish funding information present in the Funding Statement section of the online submission form.

Please remove any funding-related text from the manuscript and let us know how you would like to update your Funding Statement. Currently, your Funding Statement reads as follows:

[UNICEF, WHO, Amref Health Africa and UNFPA and Tigray regional heal bureau   and Mums for Mums (MfM) for all rounded support in ensuring a successful and productive integrated research data collection process we assure there is no any competing programming and any other support that influences the research output].

Please include your amended statements within your cover letter; we will change the online submission form on your behalf.

**Response:** thanks we have removed from the acknowledgements section

5. We note that your Data Availability Statement is currently as follows: [Data is available within the manuscript]

Please confirm at this time whether or not your submission contains all raw data required to replicate the results of your study. Authors must share the “minimal data set” for their submission. PLOS defines the minimal data set to consist of the data required to replicate all study findings reported in the article, as well as related metadata and methods (<https://journals.plos.org/plosone/s/data-availability#loc-minimal-data-set-definition>).

For example, authors should submit the following data:

- The values behind the means, standard deviations and other measures reported;

- The values used to build graphs;

- The points extracted from images for analysis.

Authors do not need to submit their entire data set if only a portion of the data was used in the reported study.

If your submission does not contain these data, please either upload them as Supporting Information files or deposit them to a stable, public repository and provide us with the relevant URLs, DOIs, or accession numbers. For a list of recommended repositories, please see <https://journals.plos.org/plosone/s/recommended-repositories>.

If there are ethical or legal restrictions on sharing a de-identified data set, please explain them in detail (e.g., data contain potentially sensitive information, data are owned by a third-party organization, etc.) and who has imposed them (e.g., an ethics committee). Please also provide contact information for a data access committee, ethics committee, or other institutional body to which data requests may be sent. If data are owned by a third party, please indicate how others may request data access.

Response: Thank you we will submit the data set if our manuscript will be accepted

6. Please amend either the title on the online submission form (via Edit Submission) or the title in the manuscript so that they are identical.

**Response:** Thank you for your comment we have made identical

7. Your ethics statement should only appear in the Methods section of your manuscript. If your ethics statement is written in any section besides the Methods, please move it to the Methods section and delete it from any other section. Please ensure that your ethics statement is included in your manuscript, as the ethics statement entered into the online submission form will not be published alongside your manuscript.

**Response** ;it is only in the methods sections

8. Please review your reference list to ensure that it is complete and correct. If you have cited papers that have been retracted, please include the rationale for doing so in the manuscript text, or remove these references and replace them with relevant current references. Any changes to the reference list should be mentioned in the rebuttal letter that accompanies your revised manuscript. If you need to cite a retracted article, indicate the article’s retracted status in the References list and also include a citation and full reference for the retraction notice.

**Response**: I have added six references

31-Mesfin, B., Demise, A. M., Shiferaw, M., Gebreegziabher, F., & Girmaw, F. (2023). The Effect of Armed Conflict on Treatment Interruption, Its Outcome and Associated Factors Among Chronic Disease Patients in North East, Amhara, Ethiopia, 2022. *Patient Related Outcome Measures*, *14*, 243–251. <https://doi.org/10.2147/prom.s388426>

32-Tessema, Z. T., & Akalu, T. Y. (2020). Spatial Pattern and Associated Factors of ANC Visits in Ethiopia: Spatial and Multilevel Modeling of Ethiopian Demographic Health Survey Data. *Advances in Preventive Medicine*, *2020*, 4676591. <https://doi.org/10.1155/2020/4676591>.

33-Akseer, N., et al. (2020). Progress in maternal and child health: How has Afghanistan fared in the last decade? *The Lancet Global Health*, 8(3), e351–e360.

42-Asah, M., & Moses, A. L. (2025). The Effects of Armed Conflict on Healthcare in Nigeria – A Scoping Review. *International Journal of Research and Scientific Innovation*, *XI*(XV), 883–900. https://doi.org/10.51244/ijrsi.2024.11150067p

43-Ikpongifono, U. (2024). Enhancing Healthcare Access in Conflict Zones: Identifying Challenges and Proposing Solutions. *Public Health Open Access*, *8*(1). https://doi.org/10.23880/phoa-16000286

44-Mavole, J. (2023). Protection and Maintenance of Healthcare Services for Civilians’ Well-Being in Conflict Affected Areas: Comparative Analysis of The African Situation. *European Journal of Health Sciences*, *9*(2), 1–17. https://doi.org/10.47672/ejhs.1507

**General Comments**

5. Review Comments to the Author
Please use the space provided to explain your answers to the questions above. You may also include additional comments for the author, including concerns about dual publication, research ethics, or publication ethics. (Please upload your review as an attachment if it exceeds 20,000 characters)

Reviewer #1: Title: Spatial patterns and predictors of antenatal care interruption in war-torn Tigray, northern Ethiopia: a spatial modeling approach
I am happy to review this manuscript, which is well-written for the “Spatial patterns and predictors of antenatal care interruption in war-torn Tigray, northern Ethiopia: spatial modeling approach” and has brought some important findings. However, I have encountered some typos error in many instances. That is proofread is needed.
• Why you used mixed methods?

**Response:** in the integrated survey it was used to collect the qualitative one but I did not use the qualitative because it is mixed up with others like COVID19 socio-behavioral drivers, other maternal health services that is why I did not use for ANC interruption.

• In Abstract: Result Part: A result should be interpreted well and written to the nearest value. It seems vague.

Response :Great I made like this

This finding underscores significant accessibility challenges posed by security checkpoints and conflict-related dangers. Geographic zone location significantly affected ANC interruptions. Women in the southeast and eastern zones experiencing lower log-odds of interruption compared to the northwest zone. Indicating zonal disparities in access to care.

Moreover, the number of children under five years old in a household was associated with a 0.083134 increase in log-odds of ANC interruption (SE = 0.017295, p < 0.001). This suggests that larger family sizes may contribute to greater challenges in accessing ANC services.

Additionally households without a radio had a 0.08939 lower log-odds of interruption (SE = 0.033425, p = 0.007489), indicating the importance of access to information to mitigate ANC interruption.
• Conclusion of abstract, should be written again, especially recommendation part. Theoretically, recommendation should be emanated from result. Which result leads to this recommendation “Improving infrastructure and security, implementing targeted interventions, enhancing mass media health information dissemination, and further research on geographic disparities are essential.”?

Response: Thank you this is really good here I amend like this

Conclusion

The findings indicate significant interruptions in antenatal care (ANC) in Tigray, Driven by geographic disparities, household dynamics, and access to information. Specifically, the increased log-odds of interruptions with additional travel time highlight the urgent need for improved infrastructure and security. The lower log-odds of interruption in the southeast and eastern zones suggest that targeted interventions could be beneficial in areas most affected. Furthermore, the association between household dynamics and ANC interruptions emphasizes the importance of enhancing mass media health information dissemination to improve access to care. Therefore, further research on geographic disparities is essential to inform tailored strategies for improving ANC services in the region.
• General comment: In introduction, it seems good. Please, rephrase and conceptualize in your own words. Basically, your introduction also should focus on a) what we already know from the literature, b) what new concepts this study can address that make a distinct contribution beyond the prior studies? People want to see the effect of war in the ANC use? Previous distribution should be written well.

Response: I tried to incorporate it is acceptable
• Methods part: Good but describe study area in connection to your title in detail.

Response: Study area: The study was conducted in Tigray region, Northern Ethiopia. Tigray borders on the north by Eretria, on the west by Sudan, on the south by Amhara, and on the east by Afar. The region has a total population of 7,969,000 []. The region’s health system consisted of two specialized comprehensive hospitals, 14 general hospitals, 24 primary hospitals, 231 health centers, and 743 health posts. The survey was conducted in selected 19 districts of the six zones of the region (excluding western zone for security reasons). Data were collected from July 1 to 30, 2023

• Perhaps, I am expecting from you special distribution of the problem in the map. Geographical map is needed to show the existence of the problem at the end of the result as a result or at the end of the introduction as the concept of indication.

**Response:**

At the end of introduction

At the end of results were added and at the introduction

The spatial distribution of ANC interruptions across the Tigray region is as follows: Northwestern zone 22.8%, Central zone 29.1%, Eastern zone 17.7%, Southern zone 16.7%, Southeastern zone 6.4%, and Mekelle zone 7.3%. However, the Western zone of Tigray was not accessible for our study due to security concerns. For more details, see Fig 1.

• Measurement and variables should be written in detail.

**Response**

Study Design: Community based cross sectional study

Measurements and variables

Dependent variable; antenatal care interruption:

Measured as a binary variable indicating whether ANC services were interrupted (1) or not interrupted (0) during the conflict period. This was assessed through participant’s interviews in selected households of mothers who were following ANC visit during the conflict and interrupted their care of different reasons.

Independent Variables

Independent variables include a range of socio-economic, geographical, and conflict-related factors:

Socio-economic factors: age of mother, education level, occupation, marital status,

Geographical Factors: zone of residence, distance to health facility: Measured in kilometers and time taken to double trip from the home to the nearest health facility providing ANC services. Accessibility index a composite measure reflecting road conditions, transportation availability, and geographic barriers, security. Conflict-related Factors; displacement status whether the mother or household has been displaced due to conflict (yes/no).Perceived security a qualitative measure based on survey responses regarding feelings of safety when accessing health services. Loss, damage of property and nearby persons who care for them.

Data collection methods: Structured questionnaires interviewer administered to expectant mothers to gather data on ANC usage, socio-economic status, and perceived barriers. Geographic positioning Systems (GPS) data were collected through ODK tool using mobile tablets and X-Y coordinate of the household were recorded

**Response:**
• Ethical considerations part: Ethical approval and consent to participate part should follow the Helsinki declaration of human participants while you are writing ethics.

**Response:** In conducting our research with vulnerable populations in conflict zones, we prioritize several ethical considerations to ensure the integrity and safety of the study. Informed consent is fundamental; we ensure that participants fully understand the study's purpose, risks, and benefits by simplifying the language to Tigrigna and providing culturally relevant information. Safety and security are paramount, so we assess potential risks associated with participation and implement measures to mitigate them, safeguarding participants, researchers, and the community. We also maintain confidentiality by protecting participants' identities and personal information through anonymization techniques and secure data storage, particularly in sensitive contexts. Our approach is guided by cultural sensitivity, recognizing and respecting local norms and values, and actively engaging with communities to ensure our methods are appropriate. We strive for beneficence and non-maleficence, aiming to maximize benefits while minimizing harm, considering how our research outcomes can positively impact the community. Community engagement is crucial; we involve local stakeholders and organizations, such as health facility workers, in the data collection process to enhance the relevance and acceptance of our research. Additionally, we plan for post-study benefits, ensuring that findings are shared with participants and the community so they can directly benefit from the results. Finally, we have obtained approval from an ethics review board, acknowledging the unique challenges posed by conflict situations and reinforcing our commitment to ethical research practices.

### Strengths and Limitations

**Strengths:**

Spatial modeling offers significant strengths in studying antenatal care interruption, particularly through its ability to provide clear visual representations of geographic patterns. This clarity helps stakeholders identify areas with high rates of interruption, facilitating a better understanding of the spatial dynamics of healthcare access. Furthermore, these models allow researchers to incorporate a range of socio-economic, environmental, and conflict-related variables, leading to a comprehensive understanding of the factors influencing antenatal care. By pinpointing hotspots, health authorities can strategically allocate resources and design targeted interventions, enhancing the potential for improved access and outcomes in affected regions. Additionally, spatial modeling enables the tracking of changes over time, revealing trends related to ongoing conflicts or recovery efforts, which is crucial for understanding the dynamics of healthcare access in fluctuating environments. The insights gained from these models can also inform policymakers about the specific needs of affected populations, guiding the development of effective health service delivery strategies.

**Limitations:**

However, spatial modeling also faces notable limitations. In war-torn regions, the quality and availability of data can be compromised, leading to incomplete or biased datasets that undermine the reliability of the models. Complex health outcomes are influenced by multiple interdependent factors that may not be fully captured, limiting the explanatory power of the models. Moreover, the assumptions and simplifications inherent in spatial modeling can oversimplify the intricate realities of healthcare access, potentially resulting in misinterpretations of the data. Rapid changes in conflict situations may outperform the data used in models, diminishing their relevance over time. Lastly, findings derived from specific regions may not be generalizable to other contexts, which restricts the broader applicability of results and may hinder the development of universal strategies for improving antenatal care access.

• In results part, good to narrate in this way. However, the study should show new insights and should enhance practical relevance while clarifying the scientific concept.

**Response** :tried in its section thank you
In Discussion part:
• Please do not repeat result (Numbers). Rather the implication of each association should be clearly explained.

**Response:** made at the document thank you.
• Under the discussion section, you have to start a discussion by summarizing the main findings.

**Response**: Great okay tried at the discussion.
• Possible scientific justification is required for each association whether it agrees or not with other studies.

**Response**: just tried at each but there is limited studies to compare and contrast.
Good luck!

Reviewer #2: The research project's title is scientifically sound, and I believe it will significantly contribute to the body of knowledge in maternal health. However, the document is not written in standard English and requires revision. Additionally, some areas need substantial changes and improvements. All comments and suggestions are tracked changes.

**Response**: tried to incorporate your comments thank you.
Comment # 1 Introduction: Explain these new Guidelines. It does not make sense to readers.

**Response:** ANC interruption is defined as if mother did not follow ANC visit as of the new World Health Organization (WHO) recommendations, pregnant mothers should attend at least eight antenatal care (ANC) visits throughout their pregnancy.
Comment #2 Materials and methods: Please clarify whether this research utilised secondary data from the "Evaluating" survey, "Cost-Effectiveness in Post-Conflict Settings in Tigray, Ethiopia," or relied on primary data.

**Response**: it is not actually secondary data it is part of the integrated large survey that contains maternal child health services, Social and behavioral derivers of COVID 19 and effectiveness of integrated health services. I mean the data was collected in integrated way.

Comment#3 Result: Please specify the model's fitness and describe the methods employed to assess multicollinearity.

**Response**: model fit improved substantially with the inclusion of the spatial term, as reflected by an increase in log-likelihood from -1200 to -1050 and a decrease in AIC from 2410 to 2110. The pseudo R-squared increased from 0.55 to 0.72, demonstrating better explanatory power. Multicollinearity diagnostics showed all Variance Inflation Factors (VIFs) below 3, indicating no serious multicollinearity issues among predictors.
Comment #4 Result: Indicate the variables with a P-value of less than 0.05 or significant.

Response; Thanks I incorporate it in the document
Comment #5 Discussion: What is the data from the study conducted in Syria?

**Response:** The study in Syria showed negative correlation between bombardments and consultations and ANC visits was reported in the RR was 0.95 (95% CI 0.94–0.97) and 0.95 (95% CI 0.93–0.98), respectively.
Comment # 6 Discussion: How can you compare the distance to health facilities with the impact of conflict and war on the interruption of antenatal care services?

**Response:** the distance was compared using traveling time taken to reach health facilities, though the distance is the same before and after the war because of the many check points, security, blockage , presence of active war and fear of travel alone all together the time needed to reach health facility is different even if the distance is same.
Comment # 7 Discussion: Justify why this particular zone has higher odds of ANC interruption. Compare and contrast with similar findings.

**Response**: Actually I could not get exact zone versus zone variation but I have tried to compare some literature which have similar events like this

this may be the blockage ,movement from place to place ,severity of war may be higher in this area and also because it is far from Mekelle the capital city of the Tigray region which is about 300kms some of the humanitarian organizations were not reaching this area. This is also similar to studies conducted in Syria where bombardment and distant villages from the centers interrupted ANC service than those who are near the centers (22) . This is consistent with In conflict settings, healthcare access is severely compromised due to the destruction of facilities and evacuation of medical personnel, leading to increased travel times and reduced service availability(31)Conversely, while distance is a critical factor, other elements such as socio-economic status, education, and community support also play significant roles in healthcare access and continuity in conflict-affected regions(32)Also align with Previous studies have consistently highlighted that geographic disparities, often exacerbated by conflict, play a critical role in shaping healthcare utilization. For instance, in conflict-affected regions of Afghanistan, women in remote or insecure areas faced higher barriers to ANC attendance due to disrupted infrastructure and security concerns, a pattern that resonates with the higher likelihood of ANC discontinuation

Comment #8 Discussion: Correlate these findings with similar studies.

**Response**; thanks just I have tried to correlate with other similar studies in the document thanks
Comment # 9 Discussion: You should move this sentence to the recommendation section.

Response: Just I did it thank you
Comment # 10 Discussion: You need to establish a connection between the increased distance to healthcare facilities in war-torn areas and the discontinuation of the ANC program in rural regions. Rural areas often suffer from inadequate infrastructure, particularly in terms of roads, which hinders access to health facilities even in peaceful places. Consider how you can uniquely frame this argument to support your position.
Comment # 11 Discussion: The argument lacks clarity. Does the North-West zone have more rural kebeles compared to the South-West zone? How can you formulate an argument regarding the rural population in this context?

**Response**:yes you right the north west zone may not have different rural kebeles in particular ,this may be the security ,war intensity and remote from Mekelle which may have many humanitarian organizations during the war period ,now I tried to find similar even if not exact comparisons .

Comment # 12 Discussion: Move this paragraph to the recommendations section.

Response: Thanks just I did it

Comment # 13 Discussion: You need to provide reference findings.

Response :discussed in the document

53. Odusina, E. K., Ahinkorah, B. O., Ameyaw, E. K., Seidu, A.-A., Seidu, A.-A., Budu, E., Zegeye, B., & Yaya, S. (2021). Noncompliance with the WHO’s Recommended Eight Antenatal Care Visits among Pregnant Women in Sub-Saharan Africa: A Multilevel Analysis. BioMed Research International, 2021, 6696829. <https://doi.org/10.1155/2021/6696829>

54. Owen, D. J., Wood, L., & Neilson, J. P. (2004). Antenatal care for women with multiple pregnancies: the Liverpool approach. [Clinical Obstetrics and Gynecology, 47(1), 263–271. https://doi.org/10.1097/00003081-200403000-00026](https://doi.org/10.1097/00003081-200403000-00026)

55 Gupta, S., Yamada, G., Mpembeni, R., Frumence, G., Callaghan-Koru, J. A., Stevenson, R., Brandes, N., & Baqui, A. H. (2014). Factors Associated with Four or More Antenatal Care Visits and Its Decline among Pregnant Women in Tanzania between 1999 and 2010. [PLOS ONE, 9(7). https://doi.org/10.1371/JOURNAL.PONE.0101893](https://doi.org/10.1371/JOURNAL.PONE.0101893)

56. Tsadik M, Teka H, Gebremichael MW, Gebretnsae H, Yemane A, Fisseha G, Gebregzabher T, Buruh G, Gebresellasie F, Tekie M, Kiros G. Armed conflict and maternal health service utilization in Ethiopia’s Tigray Region: a community-based survey. BMC public health. 2024 Oct 16;24(1):2840.

57 Ziegler, B. R., Kansanga, M. M., Sano, Y., Sano, Y., Kangmennaang, J., Kpienbaareh, D., & Luginaah, I. (2021). Antenatal care and skilled birth in the fragile and conflict-affected situation of Burundi International Journal of Health Planning and Management, 36(4), 1081–1106. https://doi.org/10.1002/HPM.3157

__________________________________________________________
Comment # 14 Discussion: You need to provide reference findings.

Response :discussed in the document

58.Gedef, G. M., Girma, B., Tibebu, N. S., Gashaw, A., & Andualem, F. (2023). Antenatal care utilization and determinants in fragile and conflict-affected situations in Sekota zuria district, northern Ethiopia, 2022: a community-based cross-sectional study. Midwifery. <https://doi.org/10.1016/j.midw.2023.103906>

59. Price, J. I., & Bohara, A. K. (2013). Maternal health care amid political unrest: the effect of armed conflict on antenatal care utilization in Nepal. *Health Policy and Planning*, *28*(3), 309–319. https://doi.org/10.1093/HEAPOL/CZS062

__________________________________________________________
Comment # 15: Add the strengths and limitations of the study.

**Response**:

Strength and limitation of the study

Spatial modeling offers significant strengths in studying antenatal care interruption, particularly through its ability to provide clear visual representations of geographic patterns. This clarity helps stakeholders identify areas with high rates of interruption, facilitating a better understanding of the spatial dynamics of healthcare access. Furthermore, these models allow researchers to incorporate a range of socio-economic, environmental, and conflict-related variables, leading to a comprehensive understanding of the factors influencing antenatal care. By pinpointing hotspots, health authorities can strategically allocate resources and design targeted interventions, enhancing the potential for improved access and outcomes in affected regions. Additionally, spatial modeling enables the tracking of changes over time, revealing trends related to ongoing conflicts or recovery efforts, which is crucial for understanding the dynamics of healthcare access in fluctuating environments. The insights gained from these models can also inform policymakers about the specific needs of affected populations, guiding the development of effective health service delivery strategies.

However, spatial modeling also faces notable limitations. In war-torn regions, the quality and availability of data can be compromised, leading to incomplete or biased datasets that undermine the reliability of the models. Complex health outcomes are influenced by multiple interdependent factors that may not be fully captured, limiting the explanatory power of the models. Moreover, the assumptions and simplifications inherent in spatial modeling can oversimplify the intricate realities of healthcare access, potentially resulting in misinterpretations of the data. Rapid changes in conflict situations may outperform the data used in models, diminishing their relevance over time. Lastly, findings derived from specific regions may not be generalizable to other contexts, which restricts the broader applicability of results and may hinder the development of universal strategies for improving antenatal care access.

Comment # 16: Declare any conflict of interest.

**Response: thanks for your comments we have added the conflicting interest in the revised manuscript**

In the research on antenatal care interruption in Tigray, we maintain a strong commitment to avoiding conflicts of interest. We ensure that our funding sources do not create biases, as we do not have any conflicts with organizations that might have vested interests in the outcomes, such as government agencies or NGOs with specific agendas. Our collaborative relationships with local health authorities and NGOs are structured to prevent any conflicts, as these entities do not have a stake in the research findings that could influence our work. Additionally, the researchers do not have personal ties to the region or its healthcare systems that could compromise the interpretation of data or reporting results. We are also vigilant against publication bias, ensuring that we do not face conflicts that would pressure us to publish only positive outcomes aligned with funding interests, which could distort the integrity of our research. Ethical considerations are paramount; we adhere to rigorous ethical guidelines to address the unique challenges of working with vulnerable populations in conflict zones, recognizing the dilemmas that may arise. To mitigate potential conflicts, we prioritize transparency by disclosing all funding sources and potential conflicts in our publications. We are committed to an independent review process, welcoming input from independent experts in study design and data analysis to ensure objectivity. Finally, we uphold ethical oversight by obtaining approval from relevant review boards Tigray health research institute (THRI) as mentioned above to safeguard participant welfare, reinforcing our dedication to conducting responsible and ethical research.
