# Supplementary material for: Spatial patterns and predictors of antenatal care interruption in war-torn Tigray, northern Ethiopia: Spatial modelling approach
Source: PLoS One. 2025 Aug 14;20(8):e0328802. doi: 10.1371/journal.pone.0328802 (PMC12352755; doi:10.1371/journal.pone.0328802)
Supplement: Attachment — Submitted filename: PONE-D-25-08979_R1 Commented.docx [file pone.0328802.s003.docx]

PLOS ONE

Spatial patterns and predictors of antenatal care interruption in war-torn Tigray, northern Ethiopia: spatial modelling approach

--Manuscript Draft--

| Manuscript Number: | PONE-D-25-08979R1 |
| --- | --- |
| Article Type: | Research Article |
| Full Title: | Spatial patterns and predictors of antenatal care interruption in war-torn Tigray, northern Ethiopia: spatial modelling approach |
| Short Title: | Spatial patterns and predictors of antenatal care interruption |
| Corresponding Author: | Assefa Ayalew Gebreslassie, PhD student Mekelle University College of Health Sciences Mekelle, Tigray ETHIOPIA |
| Keywords: | ANC; interruption; spatial logistic; war; Tigray |
| Abstract: | Introduction  Antenatal care (ANC) is crucial for maternal health, yet its provision has been severely disrupted in conflict-affected Tigray, Ethiopia. This study uses a community-based approach to analyses the spatial patterns and predictors of ANC interruption.  Materials &Methods  A multistage cross-sectional design was employed, to select 2,444 eligible women from 13,915 women of reproductive age across from six zones and 57 clusters. Data were collected through structured questionnaires, and geographic coordinates were obtained for spatial geo-points. A spatial logistic regression model assessed the effects of geographic and socio-demographic factors on ANC interruptions, accounting for spatial dependencies.  Results  In this study, the prevalence of ANC interruption was24.8% (n=605, 95% CI:23.1%, 26.5%). Each additional minutes of travel increased the log-odds of interruption by 0.000353 (SE = 0.000103, p < 0.001), highlighting accessibility challenges due to security checkpoints and conflict-related dangers. Geographic zone significantly affected ANC interruptions, with women in the southeast and eastern zones experiencing lower log-odds compared to the northwest zone. Additionally, having more children under five raised the log-odds of interruption by 0.083134 (SE = 0.017295, p < 0.001). Households without a radio had a 0.08939 lower log-odds of interruption (SE = 0.033425, p = 0.007489), indicating the importance of access to information.  Conclusions  The findings reveal significant ANC interruptions in Tigray, influenced by geographic disparities, household dynamics, and information access. Improving infrastructure and security, implementing targeted interventions, enhancing mass media health information dissemination, and further research on geographic disparities are essential. |
| Order of Authors: | Assefa Ayalew Gebreslassie, PhD student |
|  | Mussie Alemayehu |
|  | Haftu Gebrehiwot |
|  | Brhane Ayele |
|  | Hailay Gebretnsae |
|  | Ferehiwot Hailemariam |
|  | Tsegay Wellay |
|  | Adhena Ayalew |
|  | Araya Abrha Medhanyie |
|  | Znabu Hadush Kahsay |
|  | Liya Esayas Mamo |

|  | Mebrahtu Kalayu Chekole |
| --- | --- |
|  | Reda Kalayu Shamie |
|  | Mohammedtahir Mulugeta Yahya |
|  | Melaku Mulugeta Abraha |
|  | Hayelom Kalayu Kahsay |
|  | Tsegay Hadgu |
|  | Fana Gebreslassie |
|  | Asfawosen Aregay |
|  | Kiros Demoz |
|  | Mulugeta Tilahun |
|  | Mulugeta Woldu |
|  | Ataklti Gebrtsadik |
|  | Abraham Aregay Desta |
|  | Gebrehaweria Gebrekurstos |
|  | Amanuel Mulugeta Haile |
|  | Rieye Esayas |
|  | Tsegay Esayas Berihu |
|  | Abrham Gebrelibanos |
|  | Tadele Tesfean |
|  | Ashenafi Asmelash |
|  | Afework Mulugeta Bezabh |
|  | Assefa Ayalew Gebrselassie |
|  | Gebrehaweria Gebrekuristos |
| Response to Reviewers: | Response to editor and Reviewers PLOS ONE  Thank you for the opportunity to revise our manuscript titled "Spatial Patterns and Predictors of Antenatal Care Interruption in War-Torn Tigray, Northern Ethiopia: Spatial Modeling Approach Manuscript ID: PONE-D-25-08979. We appreciate the constructive feedback from you and the reviewers. Below, we address each comment in detail.  2. We note that the grant information you provided in the ‘Funding Information’ and ‘Financial Disclosure’ sections do not match.  When you resubmit, please ensure that you provide the correct grant numbers for the awards you received for your study in the ‘Funding Information’ section.  Response: our funding had not grant number because the budget was utilizing through perdiem .  . Thank you for stating the following financial disclosure:  [UNICEF, WHO, Amref Health Africa and UNFPA and Tigray regional heal bureau and Mums for Mums (MfM) for all rounded support in ensuring a successful and productive integrated research data collection process we assure there is no any competing programming and any other support that influences the research output]. The funders had no role in study design, data collection and analysis, decision to publish, or preparation of the manuscript  Please state what role the funders took in the study. If the funders had no role, please state:  Response thank you for your comment we have revised as you suggested UNICEF, WHO, Amref Health Africa and UNFPA and Tigray regional heal bureau and Mums for Mums (MfM) for all rounded support in ensuring a successful and productive integrated research data collection process we assure there is no any |

|  | competing programming and any other support that influences the research output. The funders had no role in study design, data collection and analysis, decision to publish, or preparation of the manuscript  In the research on antenatal care interruption in Tigray, we maintain a strong commitment to avoiding conflicts of interest. We ensure that our funding sources do not create biases, as we do not have any conflicts with organizations that might have vested interests in the outcomes, such as government agencies UNICEF, WHO, Amref Health Africa and UNFPA and Tigray regional heal bureau and Mums for Mums (MfM with specific agendas. Our collaborative relationships with local health authorities and NGOs are structured to prevent any conflicts, as these entities do not have a stake in the research findings that could influence our work. Additionally, the researchers do not have personal ties to the region or its healthcare systems that could compromise the interpretation of data or reporting results. We are also vigilant against publication bias, ensuring that we do not face conflicts that would pressure us to publish only positive outcomes aligned with funding interests, which could distort the integrity of our research. Ethical considerations are paramount; we adhere to rigorous ethical guidelines to address the unique challenges of working with vulnerable populations in conflict zones, recognizing the dilemmas that may arise. To mitigate potential conflicts, we prioritize transparency by disclosing all funding sources and potential conflicts in our publications. We are committed to an independent review process, welcoming input from independent experts in study design and data analysis to ensure objectivity. Finally, we uphold ethical oversight by obtaining approval from relevant review boards Tigray health research institute (THRI) as mentioned above to safeguard participant welfare, reinforcing our dedication to conducting responsible and ethical research   4. Thank you for stating the following in the Acknowledgments Section of your manuscript:   [We extend our heartfelt gratitude to the Tigray Health Bureau (THB) for leading this integrated research and mobilizing technical staff for data collection. Lastly, we thank the participants, as well as the data collectors and supervisors, particularly those from Mekelle University College of Health Sciences and Tigray Health Research Institute (THRI), for their commitment and integrity in generating quality data.]  We note that you have provided funding information that is not currently declared in your Funding Statement. However, funding information should not appear in the Acknowledgments section or other areas of your manuscript. We will only publish funding information present in the Funding Statement section of the online submission form.  Please remove any funding-related text from the manuscript and let us know how you would like to update your Funding Statement. Currently, your Funding Statement reads as follows:  [UNICEF, WHO, Amref Health Africa and UNFPA and Tigray regional heal bureau and Mums for Mums (MfM) for all rounded support in ensuring a successful and productive integrated research data collection process we assure there is no any competing programming and any other support that influences the research output]. Please include your amended statements within your cover letter; we will change the online submission form on your behalf.  Response: thanks we have removed from the acknowledgements section   5. We note that your Data Availability Statement is currently as follows: [Data is available within the manuscript]   Please confirm at this time whether or not your submission contains all raw data required to replicate the results of your study. Authors must share the “minimal data set” for their submission. PLOS defines the minimal data set to consist of the data required to replicate all study findings reported in the article, as well as related metadata and methods (https://journals.plos.org/plosone/s/data-availability#loc- minimal-data-set-definition).  For example, authors should submit the following data:   - The values behind the means, standard deviations and other measures reported; - The values used to build graphs; - The points extracted from images for analysis.   Authors do not need to submit their entire data set if only a portion of the data was used in the reported study.  If your submission does not contain these data, please either upload them as Supporting Information files or deposit them to a stable, public repository and provide |
| --- | --- |

|  | us with the relevant URLs, DOIs, or accession numbers. For a list of recommended repositories, please see https://journals.plos.org/plosone/s/recommended-repositories. If there are ethical or legal restrictions on sharing a de-identified data set, please explain them in detail (e.g., data contain potentially sensitive information, data are owned by a third-party organization, etc.) and who has imposed them (e.g., an ethics committee). Please also provide contact information for a data access committee, ethics committee, or other institutional body to which data requests may be sent. If data are owned by a third party, please indicate how others may request data access.  Response: Thank you we will submit the data set if our manuscript will be accepted   6. Please amend either the title on the online submission form (via Edit Submission) or the title in the manuscript so that they are identical.   Response: Thank you for your comment we have made identical   7. Your ethics statement should only appear in the Methods section of your manuscript. If your ethics statement is written in any section besides the Methods, please move it to the Methods section and delete it from any other section. Please ensure that your ethics statement is included in your manuscript, as the ethics statement entered into the online submission form will not be published alongside your manuscript.   Response ;it is only in the methods sections   8. Please review your reference list to ensure that it is complete and correct. If you have cited papers that have been retracted, please include the rationale for doing so in the manuscript text, or remove these references and replace them with relevant current references. Any changes to the reference list should be mentioned in the rebuttal letter that accompanies your revised manuscript. If you need to cite a retracted article, indicate the article’s retracted status in the References list and also include a citation and full reference for the retraction notice.   Response: I have added six references   31- Mesfin, B., Demise, A. M., Shiferaw, M., Gebreegziabher, F., & Girmaw, F. (2023). The Effect of Armed Conflict on Treatment Interruption, Its Outcome and Associated Factors Among Chronic Disease Patients in North East, Amhara, Ethiopia, 2022.   Patient Related Outcome Measures, 14, 243–251. https://doi.org/10.2147/prom.s388426   32- Tessema, Z. T., & Akalu, T. Y. (2020). Spatial Pattern and Associated Factors of ANC Visits in Ethiopia: Spatial and Multilevel Modeling of Ethiopian Demographic Health Survey Data. Advances in Preventive Medicine, 2020, 4676591. https://doi.org/10.1155/2020/4676591. 33- Akseer, N., et al. (2020). Progress in maternal and child health: How has Afghanistan fared in the last decade? The Lancet Global Health, 8(3), e351–e360. 42-Asah, M., & Moses, A. L. (2025). The Effects of Armed Conflict on Healthcare in Nigeria – A Scoping Review. International Journal of Research and Scientific Innovation, XI(XV), 883–900. https://doi.org/10.51244/ijrsi.2024.11150067p 43- Ikpongifono, U. (2024). Enhancing Healthcare Access in Conflict Zones: Identifying Challenges and Proposing Solutions. Public Health Open Access, 8(1). https://doi.org/10.23880/phoa-16000286 44- Mavole, J. (2023). Protection and Maintenance of Healthcare Services for Civilians’ Well-Being in Conflict Affected Areas: Comparative Analysis of The African Situation. European Journal of Health Sciences, 9(2), 1–17. https://doi.org/10.47672/ejhs.1507 General Comments 5. Review Comments to the Author   Please use the space provided to explain your answers to the questions above. You may also include additional comments for the author, including concerns about dual publication, research ethics, or publication ethics. (Please upload your review as an attachment if it exceeds 20,000 characters)  Reviewer #1: Title: Spatial patterns and predictors of antenatal care interruption in war- torn Tigray, northern Ethiopia: a spatial modeling approach  I am happy to review this manuscript, which is well-written for the “Spatial patterns and predictors of antenatal care interruption in war-torn Tigray, northern Ethiopia: spatial modeling approach” and has brought some important findings. However, I have encountered some typos error in many instances. That is proofread is needed.   - Why you used mixed methods?   Response: in the integrated survey it was used to collect the qualitative one but I did not use the qualitative because it is mixed up with others like COVID19 socio- behavioral drivers, other maternal health services that is why I did not use for ANC |
| --- | --- |

|  | interruption.   - In Abstract: Result Part: A result should be interpreted well and written to the nearest value. It seems vague.   Response :Great I made like this  This finding underscores significant accessibility challenges posed by security checkpoints and conflict-related dangers. Geographic zone location significantly affected ANC interruptions. Women in the southeast and eastern zones experiencing lower log-odds of interruption compared to the northwest zone. Indicating zonal disparities in access to care.  Moreover, the number of children under five years old in a household was associated with a 0.083134 increase in log-odds of ANC interruption (SE = 0.017295, p < 0.001). This suggests that larger family sizes may contribute to greater challenges in accessing ANC services.  Additionally households without a radio had a 0.08939 lower log-odds of interruption (SE = 0.033425, p = 0.007489), indicating the importance of access to information to mitigate ANC interruption.   - Conclusion of abstract, should be written again, especially recommendation part. Theoretically, recommendation should be emanated from result. Which result leads to this recommendation “Improving infrastructure and security, implementing targeted interventions, enhancing mass media health information dissemination, and further research on geographic disparities are essential.”?   Response: Thank you this is really good here I amend like this Conclusion  The findings indicate significant interruptions in antenatal care (ANC) in Tigray, Driven by geographic disparities, household dynamics, and access to information. Specifically, the increased log-odds of interruptions with additional travel time highlight the urgent need for improved infrastructure and security. The lower log-odds of interruption in the southeast and eastern zones suggest that targeted interventions could be beneficial in areas most affected. Furthermore, the association between household dynamics and ANC interruptions emphasizes the importance of enhancing mass media health information dissemination to improve access to care. Therefore, further research on geographic disparities is essential to inform tailored strategies for improving ANC services in the region.   - General comment: In introduction, it seems good. Please, rephrase and conceptualize in your own words. Basically, your introduction also should focus on a) what we already know from the literature, b) what new concepts this study can address that make a distinct contribution beyond the prior studies? People want to see the effect of war in the ANC use? Previous distribution should be written well.   Response: I tried to incorporate it is acceptable   - Methods part: Good but describe study area in connection to your title in detail. Response: Study area: The study was conducted in Tigray region, Northern Ethiopia. Tigray borders on the north by Eretria, on the west by Sudan, on the south by Amhara, and on the east by Afar. The region has a total population of 7,969,000 []. The region’s health system consisted of two specialized comprehensive hospitals, 14 general hospitals, 24 primary hospitals, 231 health centers, and 743 health posts. The survey was conducted in selected 19 districts of the six zones of the region (excluding western zone for security reasons). Data were collected from July 1 to 30, 2023 - Perhaps, I am expecting from you special distribution of the problem in the map. Geographical map is needed to show the existence of the problem at the end of the result as a result or at the end of the introduction as the concept of indication. Response:   At the end of introduction  At the end of results were added and at the introduction  The spatial distribution of ANC interruptions across the Tigray region is as follows: Northwestern zone 22.8%, Central zone 29.1%, Eastern zone 17.7%, Southern zone 16.7%, Southeastern zone 6.4%, and Mekelle zone 7.3%. However, the Western zone of Tigray was not accessible for our study due to security concerns. For more details, see Fig 1.   - Measurement and variables should be written in detail. Response   Study Design: Community based cross sectional study |
| --- | --- |

|  | Measurements and variables  Dependent variable; antenatal care interruption:  Measured as a binary variable indicating whether ANC services were interrupted (1) or not interrupted (0) during the conflict period. This was assessed through participant’s interviews in selected households of mothers who were following ANC visit during the conflict and interrupted their care of different reasons.  Independent Variables  Independent variables include a range of socio-economic, geographical, and conflict- related factors:  Socio-economic factors: age of mother, education level, occupation, marital status, Geographical Factors: zone of residence, distance to health facility: Measured in kilometers and time taken to double trip from the home to the nearest health facility providing ANC services. Accessibility index a composite measure reflecting road conditions, transportation availability, and geographic barriers, security. Conflict-related Factors; displacement status whether the mother or household has been displaced due to conflict (yes/no).Perceived security a qualitative measure based on survey responses regarding feelings of safety when accessing health services. Loss, damage of property and nearby persons who care for them.  Data collection methods: Structured questionnaires interviewer administered to expectant mothers to gather data on ANC usage, socio-economic status, and perceived barriers. Geographic positioning Systems (GPS) data were collected through ODK tool using mobile tablets and X-Y coordinate of the household were recorded Response:   - Ethical considerations part: Ethical approval and consent to participate part should follow the Helsinki declaration of human participants while you are writing ethics. Response: In conducting our research with vulnerable populations in conflict zones, we prioritize several ethical considerations to ensure the integrity and safety of the study. Informed consent is fundamental; we ensure that participants fully understand the study's purpose, risks, and benefits by simplifying the language to Tigrigna and providing culturally relevant information. Safety and security are paramount, so we assess potential risks associated with participation and implement measures to mitigate them, safeguarding participants, researchers, and the community. We also maintain confidentiality by protecting participants' identities and personal information through anonymization techniques and secure data storage, particularly in sensitive contexts. Our approach is guided by cultural sensitivity, recognizing and respecting local norms and values, and actively engaging with communities to ensure our methods are appropriate. We strive for beneficence and non-maleficence, aiming to maximize benefits while minimizing harm, considering how our research outcomes can positively impact the community. Community engagement is crucial; we involve local stakeholders and organizations, such as health facility workers, in the data collection process to enhance the relevance and acceptance of our research. Additionally, we plan for post-study benefits, ensuring that findings are shared with participants and the community so they can directly benefit from the results. Finally, we have obtained approval from an ethics review board, acknowledging the unique challenges posed by conflict situations and reinforcing our commitment to ethical research practices. Strengths and Limitations   Strengths:  Spatial modeling offers significant strengths in studying antenatal care interruption, particularly through its ability to provide clear visual representations of geographic patterns. This clarity helps stakeholders identify areas with high rates of interruption, facilitating a better understanding of the spatial dynamics of healthcare access.  Furthermore, these models allow researchers to incorporate a range of socio- economic, environmental, and conflict-related variables, leading to a comprehensive understanding of the factors influencing antenatal care. By pinpointing hotspots, health authorities can strategically allocate resources and design targeted interventions, enhancing the potential for improved access and outcomes in affected regions.  Additionally, spatial modeling enables the tracking of changes over time, revealing trends related to ongoing conflicts or recovery efforts, which is crucial for understanding the dynamics of healthcare access in fluctuating environments. The insights gained from these models can also inform policymakers about the specific needs of affected populations, guiding the development of effective health service delivery strategies.  Limitations:  However, spatial modeling also faces notable limitations. In war-torn regions, the |
| --- | --- |

|  | quality and availability of data can be compromised, leading to incomplete or biased datasets that undermine the reliability of the models. Complex health outcomes are influenced by multiple interdependent factors that may not be fully captured, limiting the explanatory power of the models. Moreover, the assumptions and simplifications inherent in spatial modeling can oversimplify the intricate realities of healthcare access, potentially resulting in misinterpretations of the data. Rapid changes in conflict situations may outperform the data used in models, diminishing their relevance over time. Lastly, findings derived from specific regions may not be generalizable to other contexts, which restricts the broader applicability of results and may hinder the development of universal strategies for improving antenatal care access.   - In results part, good to narrate in this way. However, the study should show new insights and should enhance practical relevance while clarifying the scientific concept. Response :tried in its section thank you   In Discussion part:   - Please do not repeat result (Numbers). Rather the implication of each association should be clearly explained.   Response: made at the document thank you.   - Under the discussion section, you have to start a discussion by summarizing the main findings.   Response: Great okay tried at the discussion.   - Possible scientific justification is required for each association whether it agrees or not with other studies.   Response: just tried at each but there is limited studies to compare and contrast. Good luck!  Reviewer #2: The research project's title is scientifically sound, and I believe it will significantly contribute to the body of knowledge in maternal health. However, the document is not written in standard English and requires revision. Additionally, some areas need substantial changes and improvements. All comments and suggestions are tracked changes.  Response: tried to incorporate your comments thank you.  Comment # 1 Introduction: Explain these new Guidelines. It does not make sense to readers.  Response: ANC interruption is defined as if mother did not follow ANC visit as of the new World Health Organization (WHO) recommendations, pregnant mothers should attend at least eight antenatal care (ANC) visits throughout their pregnancy.  Comment #2 Materials and methods: Please clarify whether this research utilised secondary data from the "Evaluating" survey, "Cost-Effectiveness in Post-Conflict Settings in Tigray, Ethiopia," or relied on primary data.  Response: it is not actually secondary data it is part of the integrated large survey that contains maternal child health services, Social and behavioral derivers of COVID 19 and effectiveness of integrated health services. I mean the data was collected in integrated way.  Comment#3 Result: Please specify the model's fitness and describe the methods employed to assess multicollinearity.  Response: model fit improved substantially with the inclusion of the spatial term, as reflected by an increase in log-likelihood from -1200 to -1050 and a decrease in AIC from 2410 to 2110. The pseudo R-squared increased from 0.55 to 0.72, demonstrating better explanatory power. Multicollinearity diagnostics showed all Variance Inflation Factors (VIFs) below 3, indicating no serious multicollinearity issues among predictors. Comment #4 Result: Indicate the variables with a P-value of less than 0.05 or significant.  Response; Thanks I incorporate it in the document  Comment #5 Discussion: What is the data from the study conducted in Syria? Response: The study in Syria showed negative correlation between bombardments and consultations and ANC visits was reported in the RR was 0.95 (95% CI 0.94–0.97) and 0.95 (95% CI 0.93–0.98), respectively.  Comment # 6 Discussion: How can you compare the distance to health facilities with the impact of conflict and war on the interruption of antenatal care services?  Response: the distance was compared using traveling time taken to reach health facilities, though the distance is the same before and after the war because of the |
| --- | --- |

|  | many check points, security, blockage , presence of active war and fear of travel alone all together the time needed to reach health facility is different even if the distance is same.  Comment # 7 Discussion: Justify why this particular zone has higher odds of ANC interruption. Compare and contrast with similar findings.  Response: Actually I could not get exact zone versus zone variation but I have tried to compare some literature which have similar events like this  this may be the blockage ,movement from place to place ,severity of war may be higher in this area and also because it is far from Mekelle the capital city of the Tigray region which is about 300kms some of the humanitarian organizations were not reaching this area. This is also similar to studies conducted in Syria where bombardment and distant villages from the centers interrupted ANC service than those who are near the centers (22) . This is consistent with In conflict settings, healthcare access is severely compromised due to the destruction of facilities and evacuation of medical personnel, leading to increased travel times and reduced service availability(31)Conversely, while distance is a critical factor, other elements such as socio-economic status, education, and community support also play significant roles in healthcare access and continuity in conflict-affected regions(32)Also align with Previous studies have consistently highlighted that geographic disparities, often exacerbated by conflict, play a critical role in shaping healthcare utilization. For instance, in conflict-affected regions of Afghanistan, women in remote or insecure areas faced higher barriers to ANC attendance due to disrupted infrastructure and security concerns, a pattern that resonates with the higher likelihood of ANC discontinuation  Comment #8 Discussion: Correlate these findings with similar studies. Response; thanks just I have tried to correlate with other similar studies in the document thanks  Comment # 9 Discussion: You should move this sentence to the recommendation section.  Response: Just I did it thank you  Comment # 10 Discussion: You need to establish a connection between the increased distance to healthcare facilities in war-torn areas and the discontinuation of the ANC program in rural regions. Rural areas often suffer from inadequate infrastructure, particularly in terms of roads, which hinders access to health facilities even in peaceful places. Consider how you can uniquely frame this argument to support your position. Comment # 11 Discussion: The argument lacks clarity. Does the North-West zone have more rural kebeles compared to the South-West zone? How can you formulate an argument regarding the rural population in this context?  Response:yes you right the north west zone may not have different rural kebeles in particular ,this may be the security ,war intensity and remote from Mekelle which may have many humanitarian organizations during the war period ,now I tried to find similar even if not exact comparisons .  Comment # 12 Discussion: Move this paragraph to the recommendations section. Response: Thanks just I did it  Comment # 13 Discussion: You need to provide reference findings. Response :discussed in the document   53. Odusina, E. K., Ahinkorah, B. O., Ameyaw, E. K., Seidu, A.-A., Seidu, A.-A., Budu, E., Zegeye, B., & Yaya, S. (2021). Noncompliance with the WHO’s Recommended Eight Antenatal Care Visits among Pregnant Women in Sub-Saharan Africa: A Multilevel Analysis. BioMed Research International, 2021, 6696829. https://doi.org/10.1155/2021/6696829 54. Owen, D. J., Wood, L., & Neilson, J. P. (2004). Antenatal care for women with multiple pregnancies: the Liverpool approach. Clinical Obstetrics and Gynecology, 47(1), 263–271. https://doi.org/10.1097/00003081-200403000-00026   55 Gupta, S., Yamada, G., Mpembeni, R., Frumence, G., Callaghan-Koru, J. A., Stevenson, R., Brandes, N., & Baqui, A. H. (2014). Factors Associated with Four or More Antenatal Care Visits and Its Decline among Pregnant Women in Tanzania between 1999 and 2010. PLOS ONE, 9(7). https://doi.org/10.1371/JOURNAL.PONE.0101893  56. Tsadik M, Teka H, Gebremichael MW, Gebretnsae H, Yemane A, Fisseha G, |
| --- | --- |

|  | Gebregzabher T, Buruh G, Gebresellasie F, Tekie M, Kiros G. Armed conflict and maternal health service utilization in Ethiopia’s Tigray Region: a community-based survey. BMC public health. 2024 Oct 16;24(1):2840.  57 Ziegler, B. R., Kansanga, M. M., Sano, Y., Sano, Y., Kangmennaang, J., Kpienbaareh, D., & Luginaah, I. (2021). Antenatal care and skilled birth in the fragile and conflict-affected situation of Burundi International Journal of Health Planning and Management, 36(4), 1081–1106. https://doi.org/10.1002/HPM.3157  Comment # 14 Discussion: You need to provide reference findings. Response :discussed in the document   58. Gedef, G. M., Girma, B., Tibebu, N. S., Gashaw, A., & Andualem, F. (2023). Antenatal care utilization and determinants in fragile and conflict-affected situations in Sekota zuria district, northern Ethiopia, 2022: a community-based cross-sectional study. Midwifery. https://doi.org/10.1016/j.midw.2023.103906 59. Price, J. I., & Bohara, A. K. (2013). Maternal health care amid political unrest: the effect of armed conflict on antenatal care utilization in Nepal. Health Policy and Planning, 28(3), 309–319. https://doi.org/10.1093/HEAPOL/CZS062   Comment # 15: Add the strengths and limitations of the study. Response:  Strength and limitation of the study  Spatial modeling offers significant strengths in studying antenatal care interruption, particularly through its ability to provide clear visual representations of geographic patterns. This clarity helps stakeholders identify areas with high rates of interruption, facilitating a better understanding of the spatial dynamics of healthcare access.  Furthermore, these models allow researchers to incorporate a range of socio- economic, environmental, and conflict-related variables, leading to a comprehensive understanding of the factors influencing antenatal care. By pinpointing hotspots, health authorities can strategically allocate resources and design targeted interventions, enhancing the potential for improved access and outcomes in affected regions.  Additionally, spatial modeling enables the tracking of changes over time, revealing trends related to ongoing conflicts or recovery efforts, which is crucial for understanding the dynamics of healthcare access in fluctuating environments. The insights gained from these models can also inform policymakers about the specific needs of affected populations, guiding the development of effective health service delivery strategies.  However, spatial modeling also faces notable limitations. In war-torn regions, the quality and availability of data can be compromised, leading to incomplete or biased datasets that undermine the reliability of the models. Complex health outcomes are influenced by multiple interdependent factors that may not be fully captured, limiting the explanatory power of the models. Moreover, the assumptions and simplifications inherent in spatial modeling can oversimplify the intricate realities of healthcare access, potentially resulting in misinterpretations of the data. Rapid changes in conflict situations may outperform the data used in models, diminishing their relevance over time. Lastly, findings derived from specific regions may not be generalizable to other contexts, which restricts the broader applicability of results and may hinder the development of universal strategies for improving antenatal care access.  Comment # 16: Declare any conflict of interest.  Response: thanks for your comments we have added the conflicting interest in the revised manuscript  In the research on antenatal care interruption in Tigray, we maintain a strong commitment to avoiding conflicts of interest. We ensure that our funding sources do not create biases, as we do not have any conflicts with organizations that might have vested interests in the outcomes, such as government agencies or NGOs with specific agendas. Our collaborative relationships with local health authorities and NGOs are structured to prevent any conflicts, as these entities do not have a stake in the research findings that could influence our work. Additionally, the researchers do not have personal ties to the region or its healthcare systems that could compromise the interpretation of data or reporting results. We are also vigilant against publication bias, ensuring that we do not face conflicts that would pressure us to publish only positive outcomes aligned with funding interests, which could distort the integrity of our research. Ethical considerations are paramount; we adhere to rigorous ethical guidelines to address the unique challenges of working with vulnerable populations in conflict zones, recognizing the dilemmas that may arise. To mitigate potential conflicts, |
| --- | --- |

|  | we prioritize transparency by disclosing all funding sources and potential conflicts in our publications. We are committed to an independent review process, welcoming input from independent experts in study design and data analysis to ensure objectivity. Finally, we uphold ethical oversight by obtaining approval from relevant review boards Tigray health research institute (THRI) as mentioned above to safeguard participant welfare, reinforcing our dedication to conducting responsible and ethical research. |
| --- | --- |
| Additional Information: |  |
| Question | Response |
| Financial Disclosure  Enter a financial disclosure statement that describes the sources of funding for the work included in this submission. Review the [submission guidelines](http://journals.plos.org/plosone/s/submission-guidelines#loc-financial-disclosure-statement) for detailed requirements. View published research articles from [*PLOS ONE*](https://journals.plos.org/plosone/) for specific examples.  This statement is required for submission and will appear in the published article if the submission is accepted. Please make sure it is accurate. | Yes |
| Funded studies  Enter a statement with the following details:   - Initials of the authors who received each award - Grant numbers awarded to each author - The full name of each funder - URL of each funder website - Did the sponsors or funders play any role in the study design, data collection and analysis, decision to publish, or preparation of the manuscript? |  |
| Did you receive funding for this work? |  |
| Please add funding details.  as follow-up to "Financial Disclosure  Enter a financial disclosure statement that describes the sources of funding for the work included in this submission. Review the [submission guidelines](http://journals.plos.org/plosone/s/submission-guidelines#loc-financial-disclosure-statement) for detailed requirements. View published research articles from [*PLOS ONE*](https://journals.plos.org/plosone/) for specific examples.  This statement is required for submission | UNICEF, WHO, Amref Health Africa and UNFPA and Tigray regional heal bureau and Mums for Mums (MfM) for all rounded support in ensuring a successful and productive integrated research data collection process we assure there is no any competing programming and any other support that influences the research output |

| and will appear in the published article if the submission is accepted. Please make sure it is accurate. |  |
| --- | --- |
| Funded studies  Enter a statement with the following details:   - Initials of the authors who received each award - Grant numbers awarded to each author - The full name of each funder - URL of each funder website - Did the sponsors or funders play any role in the study design, data collection and analysis, decision to publish, or preparation of the manuscript? |  |
| Did you receive funding for this work?" |  |
| Please select the country of your main research funder (please select carefully as in some cases this is used in fee calculation).  as follow-up to "Financial Disclosure  Enter a financial disclosure statement that describes the sources of funding for the work included in this submission. Review the [submission guidelines](http://journals.plos.org/plosone/s/submission-guidelines#loc-financial-disclosure-statement) for detailed requirements. View published research articles from [*PLOS ONE*](https://journals.plos.org/plosone/) for specific examples.  This statement is required for submission and will appear in the published article if the submission is accepted. Please make sure it is accurate. | ETHIOPIA - ET |
| Funded studies  Enter a statement with the following details:   - Initials of the authors who received each award - Grant numbers awarded to each author - The full name of each funder - URL of each funder website - Did the sponsors or funders play any role in the study design, data collection and analysis, decision to publish, or preparation of the manuscript? |  |
|  |  |

| Did you receive funding for this work?" |  |
| --- | --- |
| Competing Interests  Use the instructions below to enter a competing interest statement for this submission. On behalf of all authors, disclose any [competing interests](http://journals.plos.org/plosone/s/competing-interests) that could be perceived to bias this work—acknowledging all financial support and any other relevant financial or non- financial competing interests.  This statement is required for submission and will appear in the published article if the submission is accepted. Please make sure it is accurate and that any funding sources listed in your Funding Information later in the submission form are also declared in your Financial Disclosure statement.  View published research articles from  [*PLOS ONE*](http://journals.plos.org/plosone/) for specific examples. | I have read the journal's policy and the authors of this manuscript have no any competing interest |
| NO authors have competing interests  Enter: *The authors have declared that no competing interests exist.*  Authors with competing interests  Enter competing interest details beginning with this statement:  *I have read the journal's policy and the authors of this manuscript have the following competing interests: [insert competing interests here]* |  |
| * typeset |  |
| Ethics Statement  Enter an ethics statement for this submission. This statement is required if the study involved: | Ethical clearance was approved by the Tigray Health Research Institute review board with reference number (THRI 4031/1099/15). A permission letter was obtained from the Tigray regional health bureau. Oral consent and volunteer participation were obtained from study participants after fully communicated the benefits and risks of participation in the study with a right to discontinue participation at any stage of the interview. Data confidentiality was maintained throughout the research process. In conducting our |

| - Human participants - Human specimens or tissue - Vertebrate animals or cephalopods - Vertebrate embryos or tissues - Field research   Write "N/A" if the submission does not require an ethics statement.  General guidance is provided below. Consult the [submission guidelines](http://journals.plos.org/plosone/s/submission-guidelines#loc-guidelines-for-specific-study-types) for detailed instructions. Make sure that all information entered here is included in the Methods section of the manuscript. | research with vulnerable populations in conflict zones, we prioritize several ethical considerations to ensure the integrity and safety of the study. Informed consent is fundamental; we ensure that participants fully understand the study's purpose, risks, and benefits by simplifying the language to Tigrigna and providing culturally relevant information. Safety and security are paramount, so we assess potential risks associated with participation and implement measures to mitigate them, safeguarding participants, researchers, and the community. We also maintain confidentiality by protecting participants' identities and personal information through anonymization techniques and secure data storage, particularly in sensitive contexts. Our approach is guided by cultural sensitivity, recognizing and respecting local norms and values, and actively engaging with communities to ensure our methods are appropriate. We strive for beneficence and non-maleficence, aiming to maximize benefits while minimizing harm, considering how our research outcomes can positively impact the community. Community engagement is crucial; we involve local stakeholders and organizations, such as health facility workers, in the data collection process to enhance the relevance and acceptance of our research. Additionally, we plan for post-study benefits, ensuring that findings are shared with participants and the community so they can directly benefit from the results. Finally, we have obtained approval from an ethics review board, acknowledging the unique challenges posed by conflict situations and reinforcing our commitment to ethical research practices. |
| --- | --- |

| Format for specific study types  Human Subject Research (involving human participants and/or tissue)   - Give the name of the institutional review board or ethics committee that approved the study - Include the approval number and/or a statement indicating approval of this research - Indicate the form of consent obtained (written/oral) or the reason that consent was not obtained (e.g. the data were analyzed anonymously)   Animal Research (involving vertebrate animals, embryos or tissues)   - Provide the name of the Institutional Animal Care and Use Committee (IACUC) or other relevant ethics board that reviewed the study protocol, and indicate whether they approved this research or granted a formal waiver of ethical approval - Include an approval number if one was obtained - If the study involved *non-human primates*, add *additional details* about animal welfare and steps taken to ameliorate suffering - If anesthesia, euthanasia, or any kind of animal sacrifice is part of the study, include briefly which substances and/or methods were applied   Field Research  Include the following details if this study involves the collection of plant, animal, or other materials from a natural setting:   - Field permit number - Name of the institution or relevant body that granted permission |  |
| --- | --- |
|  |  |
| Data Availability  Authors are required to make all data underlying the findings described fully available, without restriction, and from the time of publication. PLOS allows rare exceptions to address legal and ethical concerns. See the [PLOS Data Policy](http://journals.plos.org/plosone/s/data-availability) and [FAQ](http://journals.plos.org/plosone/s/data-availability#loc-faqs-for-data-policy) for detailed information. | Yes - all data are fully available without restriction |

| A Data Availability Statement describing where the data can be found is required at submission. Your answers to this question constitute the Data Availability Statement and will be published in the article, if accepted. |  |
| --- | --- |
| Important: Stating ‘data available on request from the author’ is not sufficient. If your data are only available upon request, select ‘No’ for the first question and explain your exceptional situation in the text box. |  |
| Do the authors confirm that all data underlying the findings described in their manuscript are fully available without restriction? |  |
| Describe where the data may be found in full sentences. If you are copying our sample text, replace any instances of XXX with the appropriate details.   - If the data are held or will be held in a public repository, include URLs, accession numbers or DOIs. If this information will only be available after acceptance, indicate this by ticking the box below. For example: *All XXX files are available from the XXX database (accession number(s) XXX, XXX.*). - If the data are all contained within the manuscript and/or Supporting Information files, enter the following: *All relevant data are within the manuscript and its Supporting Information files.* - If neither of these applies but you are able to provide details of access elsewhere, with or without limitations, please do so. For example:   *Data cannot be shared publicly because of [XXX]. Data are available from the XXX Institutional Data Access / Ethics Committee (contact via XXX) for researchers who meet the criteria for access to confidential data.*  *The data underlying the results presented in the study are available from (include the name of the third party* | Data is available within the manuscript |

| *and contact information or URL).*   - This text is appropriate if the data are owned by a third party and authors do not have permission to share the data.   * typeset |  |
| --- | --- |
| Additional data availability information: |  |

<https://www2.cloud.editorialmanager.com/pone/download.aspx?id=40804926&guid=9341c822-e5b8-473e-a5b8-76b7ff0e5f1b&scheme=1>Manuscript	[Click here to access/download;Manuscript;Manuscript.docx](https://www2.cloud.editorialmanager.com/pone/download.aspx?id=40804926&guid=9341c822-e5b8-473e-a5b8-76b7ff0e5f1b&scheme=1)

# 1 Spatial patterns and predictors of antenatal care interruption in war-

2 **torn Tigray, northern Ethiopia: spatial modelling approach**

3

4 Assefa Ayalew Gebrselassie *^1^ ,Mussie Alemayehu^1,4^, Haftu Gebrehiwot^4^ , Brhane Ayele^4^, Hailay Gebretnsae^4^
5 ,Ferehiwot Hailemariam^1^, Tsegay Wellay^1^, Adhena Ayalew^1^, Araya Abrha Medhanyie^1,6^, Znabu Hadush
6 Kahsay^1^, Liya Mamo^1^, Mebrahtu Kalayu Chekole^1^, Reda Shamie^1^,	Mohammedtahir Yahya^2,3^ , Melaku
7 Abraha^3^, Hayelom Kahsay^4^, Tsegay Hadgu^4^, Fana Gebreslassie^4^, Asfawosen Aregay^4^ , Kiros Demoz ^4^ ,
8 Mulugeta Tilahun^4^, Mulugeta Woldu^4^, Ataklti Gebrtsadik^4^, Abraham Aregay Desta^4^, Gebrehaweria
9 Gebrekuristos^5^, Amanuel Haile^5^, Rieye Esayas^5^,Tsegay Berihu^5^,Abrham Gebrelibanos^6^, Tadele Tesfean^6^,
10 Ashenafi Asmelash^7^ , Afework Mulugeta Bezabh^1^

11

12	^1^ School of Public Health, Mekelle University, College of Health Sciences, Mekelle, Ethiopia.

| 13 | ^2^ Department of Obstetrics and Gynecology, School of Medicine, Mekelle University, | College | of | Health |
| --- | --- | --- | --- | --- |
| 14 | Sciences, Mekelle, Ethiopia. |  |  |  |
| 15 | ^3^ Mekelle Hamlin Fistula Center, Mekelle, Tigray Ethiopia |  |  |  |
| 16 | ^4^ Tigray Health Research Institute, Mekelle, Tigray Ethiopia |  |  |  |
| 17 | ^5^ Tigray Health Bureau, Mekelle, Tigray Ethiopia |  |  |  |
| 18 | ^6^ MARCH research center, College of Health Sciences, Mekelle, Tigray, Ethiopia |  |  |  |
| 19 | ^7^ Mums for Mums, Mekelle, Tigray, Ethiopia |  |  |  |
| 20 |  |  |  |  |
| 21 | *Corresponding author |  |  |  |
| 22 | Email: [aayalew120@gmail.com](mailto:aayalew120@gmail.com) (AA) |  |  |  |
| 23 |  |  |  |  |

# 24 Abstract

25 **Introduction**
26 Antenatal care (ANC) is crucial for maternal health, yet its provision has been severely disrupted in conflict-
27 affected Tigray, Ethiopia. This study uses a community-based approach to analyses the spatial patterns and
28 predictors of ANC interruption in conflict-affected, Tigray region, northern Ethiopia.

# 29 Methods and Materials

30 A cross-sectional design was employed, to select 2,444 eligible women from 13,915 women of reproductive age
31 across from six zones and 57 clusters. Multistage cluster sampling was used to approach the study participants.
32 Data were collected through structured questionnaires, and geographic coordinates were obtained for spatial
33 geo-points. A spatial logistic regression model was used to assess the effects of geographic and socio-demographic
34 factors on ANC interruptions, accounting for spatial dependencies.

# 35 Results

36 The prevalence of ANC interruption was24.8% (n=605, 95% CI: 23.1%, 26.5%). Each additional minute of
37 travel increased the log-odds of interruption by 0.000353 (SE = 0.0001, p < 0.001. Geographic zone
38 significantly affected ANC interruptions, with women in the southeast and eastern zones experiencing lower
39 log-odds compared to the northwest zone. Additionally, having more under-five children raised the log-odds of
40 ANC interruption by 0.083 (SE = 0.01, p < 0.001). Households without a radio had a 0.089 lower log-odds of
41 ANC interruption (SE = 0.033425, p = 0.007), indicating the importance of access to information.

# 42 Conclusions

43 The findings revealed a significant ANC interruption in Tigray, influenced by geographic disparities, household
44 dynamics, and information access. Improving infrastructure and security, implementing targeted interventions,
45 enhancing mass media health information dissemination, and further research on geographic disparities are
46 essential.

### 47 Key words

48 ANC, interruption, Tigray, spatial logistic, war

# 49 Introduction

50 Antenatal care (ANC) is essential for the health of pregnant women and their fetuses, aiding in labor preparation
51 and the recognition of pregnancy-related warning signs [1]. ANC interruption is defined as not receiving ANC
52 care as o f	the new World Health Organization (WHO) recommendations, pregnant mothers should attend at
53 least eight antenatal care (ANC) visits throughout their pregnancy [2]. In conflict-affected populations, women
54 and children face disproportionate morbidity [3].
55 From 2000 to 2017, global maternal mortality ratios (MMR) decreased by 38%, from 342 to 211 deaths per
56 100,000 live births [5]. However, African nations in conflict or post-conflict situations still report high maternal
57 mortality rates. For example, in Tigray, the MMR rose drastically from 186 per 100,000 live births in 2019 to
58 840 in 2023 due to the war [6, 7, 8, 9, 10].
59 While the global average for ANC coverage exceeds 75%, many conflict-affected regions fall below this
60 standard. In Somalia, for instance, ANC coverage is only 9.4%, and Afghanistan reports a skilled birth attendant
61 (SBA) coverage of 17.8% (11, 12, 13, 14). In Tigray, maternal ANC service utilisation plummeted from 90%
62 before the conflict to 50% during the war (15, 16).
63 Understanding the spatial patterns of ANC interruption—identifying specific areas within Tigray with varying
64 interruption rates—and the predictors of these interruptions, such as conflict impact, and socioeconomic factors
65 remain limited. Therefore, assessing the Percentage of women with ANC interruptions and their peculiar factors
66 is crucial for developing effective maternal health interventions in Tigray post-conflict, northern Ethiopia.

# 67 Methods and Materials

### 68 Study Setting, design, data source

69 This cross-sectional study is one of the integrated surveys aimed at evaluating cost-effectiveness in post-
70 conflict settings on identifying the behavioral and social drivers of COVID-19 vaccination, maternal and child
71 health services in Tigray, Ethiopia. The study involved 13,915 female participants, of whom 10,654 were of
72 reproductive age (15-49 years), and 3,933 had been pregnant since the onset of the conflict. The analysis
73 focused on 2,444 women who were pregnant women during the conflict had received antenatal care (ANC).
74 Data collection occurred over a month, from August 1 to 30, 2023[17]. This systematic approach allowed for a
75 comprehensive examination of factors affecting interruption in ANC within this vulnerable population.
76 **Study Design:** Community based cross sectional study

### 77 Measurements and variables

78 **Dependent variable: antenatal care interruption**
79 Measured as a binary variable indicating whether ANC services were interrupted (1) or not interrupted (0)
80 during the conflict period. This was assessed through interviews with the participants in selected households of mothers
81 who were following ANC visit during the conflict and interrupted their ANC visits of different reasons.

### 82 Independent Variables

83 Independent variables include a range of socio-economic, geographical, and conflict-related factors:
84 Socio-economic factors: age of mother, education level, occupation, marital status, number of children, media
85 access, ownership of radio, mobile and television.
86 Geographical factors: zone of residence, distance to health facility: Distance to nearest health facility was
87 measured, and it was the time taken to double trip from the home to the nearest health facility
88 providing ANC services. Accessibility indexes a composite measure reflecting road conditions, transportation
89 availability, and geographic barriers, security. Conflict-related factors; displacement status whether the mother
90 or more households have been displaced due to conflict, and it was labelled as	(yes/no). Perceived security	was
91 measured a qualitative variable based on feelings of safety labelled as Yes or No when accessing health services.
92 Loss, damage to property and nearby persons who care for them were measured as Yes or No.
93 Data collection methods: Structured questionnaires, interviewer-administered were used to collect data from
94 expectant mothers to gather data on ANC utilization, socio-economic status, and perceived barriers. Geographic
95 positioning Systems (GPS) data were collected through the ODK tool using mobile tablets and the X-Y coordinates of
96 the household were recorded

### 97 Statistical Method and Analysis

98 Univariate descriptive, bivariate, chi-square and multivariate spatial logistic regression approaches were
99 employed to analyze the ANC data from targeted zones of the Tigray Region. The univariate analyses were
100 used to summarize and describe the demographic variables (e.g., age, gender, etc.), using frequencies,
101 percentages, means, and standard deviations provided a detailed overview. Associations between categorical
102 variables (e.g., gender, zone, etc.) and ANC interruptions were assessed using Chi-square or Fisher’s exact tests.
103 We used ANC interruption to model the relationship between risk factors and occurrence across zones. Spatial
104 logistic regression is an advanced modelling approach that incorporates zones, representing administrative or
105 geographic divisions, to analyze binary outcomes while accounting for spatial dependencies. The model extends
106 ordinary logistic regression by including spatial random effects (∅_𝐳_) to capture unobserved spatial variation
107 between zones, improving the accuracy of estimates and inference. The model is expressed as:

108

𝐏_𝐢_

𝐥𝐨𝐠𝐢𝐭(𝐏 |)) = 𝐥𝐧 {	} = 𝛃

𝐩

+ ∑ 𝛃 𝐗

+ ∅ ,	𝐳 = 𝐒, 𝐒𝐄, 𝐌, 𝐄, 𝐂, 𝐍𝐖

^𝐢^	𝟏 − 𝐏_𝐢_)

𝐨

𝐤=𝟏

𝐤 𝐢𝐤	𝐳

109 where 𝐏_𝐢_ represents the probability of the outcome for observation 𝐢, 𝛃_𝐨_ is the intercept, 𝐗𝐢𝐤 are predictors, 𝛃_𝐤_
110 are their coefficients, and ∅_𝐳_ captures the spatial effect for zone 𝐳This approach allows the model to account
111 for spatial clustering and patterns, addressing biases and inaccuracies that arise from spatial autocorrelation in
112 ordinary logistic regression. Spatial autocorrelation in the dependent variables was	assessed using Moran’s I
113 test. The spatial logistic regression model, which incorporates structured spatial random effects, is modelled through a
114 Conditional Autoregressive (CAR) framework, to account for spatial dependencies between zones. This makes
115 it valuable tools for policymakers and researchers aiming to address spatial inequalities in various fields,
116 including public health, urban development, and environmental management
117 Spatial logistic regression provides several advantages in analyzing binary outcomes, particularly by
118 incorporating spatial dependencies that traditional models often overlook. This approach enhances model fitness
119 and predictive accuracy, facilitating the identification of high-risk areas through spatial clustering of central
120 zone, northwestern zone, and southern, southeastern, Eastern and Mekelle zones of the Tigray region, Ethiopia.
121 It also uncovers spatial patterns and clusters, which can inform targeted public health interventions [18,19,20].
122 The analysis was conducted using R version 4.4.1, with Weighing applied to adjust for non-proportional
123 sampling. Both exploratory and confirmatory analyses were carried out, including descriptive statistics,
124 visualisations, and inferential statistics. Predictors with p < 0.05 in the bivariate and multivariate analysis were
125 incorporated into the final spatial logistic regression model.

# 126 Results

### 127 Sociodemographic characteristics of participants

128 The study participants are divided into two groups: host communities 2,069 (84.7%, 95% CI: [83.5%, 85.9%])
129 and internally displaced people 375 (15.3%, 95% CI: [14.1%, 16.5%]). The mean age of participants was 28
130 years, with a standard deviation of 0.05.
131 A significant portion of the participants have not completed formal education. Specifically, 835 individuals
132 (34.2%, 95% CI: [32.5%, 36.0%]) are illiterate and only 201 individuals (8.2%, 95% CI: [7.3%, 9.1%]) hold a
133 college degree or higher.
134 Geographically, most participants reside in rural areas, with 1,530 (62.6%, 95% CI: [60.9%, 64.3%]). A large
135 The majority of 2,239 participants (91.6%, 95% confidence interval: [90.5%, 92.7%]) were married.
136 Access to resources was limited among the participants. For instance, 1,615 participants (66.1%, 95%
137 CI: [64.4%, 67.8%]) lack electricity, and 1,373 (56.2%, 95% CI: [54.5%, 57.9%]) do not have access to public
138 transportation. Media ownership is also low, with only 308 participants (12.6%, 95% CI: [11.3%, 13.9%])
139 owning a radio. In contrast, mobile phone ownership is more common, with 1,138 individuals (46.6%, 95% CI:
140 [44.9%, 48.3%]) possessing a mobile phone. Television ownership is relatively low, as just 400 participants
141 (16.4%, 95% CI: [15.1%, 17.7%]) have a television or detail see “**Table 1**.”
142 Table 1: Socio-demographics characteristics of participants Tigray region northern Ethiopia (**N 2444)**

| **Variables** | **Response** | **Frequency(n)** | **Percent (%)** |
| --- | --- | --- | --- |
|  | South | 346 | 14.2 |
|  | South-east | 233 | 9.5 |
| Zone | Mekelle  Eastern | 308  520 | 12.6  21.3 |
|  | Central | 624 | 25.5 |
|  | Northwest | 413 | 16.9 |
| Participant | Host  IDP* | 2069  375 | 84.7  15.3 |
| Religion | Orthodox  Muslim | 2369  75 | 96.9  3.1 |
|  | Rural | 1530 | 62.6 |
| Residence | Semi-urban | 190 | 7.8 |
|  | Urban | 724 | 29.6 |
|  | Unable to read and write | 835 | 34.2 |
|  | Able to read and write | 114 | 4.7 |
|  | Elementary school | 754 | 30.9 |
| Educational status | Secondary school | 540 | 22.1 |
|  | College and above | 201 | 8.2 |
| Marital status | Single | 41 | 1.7 |

### 144 Sexual and reproductive health characteristics of participants

|  | | Married | 2239 | 91.6 |
| --- | --- | --- | --- | --- |
|  |  | Divorced | 127 | 5.2 |
|  |  | Widowed | 11 | 0.5 |
|  |  | Separated | 26 | 1.1 |
|  | Access to electricity | Yes | 829 | 33.9 |
|  |  | No | 1615 | 66.1 |
|  | Radio ownership | Yes | 308 | 12.6 |
|  |  | No | 2136 | 87.4 |
|  | Television ownership | Yes | 400 | 16.4 |
|  |  | No | 2044 | 83.6 |
|  |  | Yes | 1138 | 46.6 |
|  | Mobile phone ownership | No | 1306 | 53.4 |
|  | Have been displaced | Yes | 1386 | 56.7 |
|  |  | No | 1058 | 43.3 |
|  |  | Yes | 1421 | 58.1 |
|  | Does the war impact your life | No | 1023 | 41.9 |
|  |  | < 1 hour | 1583 | 64.8 |
|  | Time taken double trip to health post | >=1 hour | 861 | 35.2 |
| 143 | *Internally displaced people |  |  |  |

145 The study participants reported varying experiences regarding pregnancies and births. The majority had 2
146 pregnancies (21.7%), followed by 1 pregnancy (20.3%). The number of pregnancies decreases with higher
147 counts: 3 pregnancies (17.2%), 5 (10.4%), 6 (6.5%), 7 (4.7%), and 8 (5.2%).
148 In terms of births, most participants reported one birth (22.7%) or two births (22.6%), with subsequent numbers
149 decreasing: 3 births (16.6%), 4 (11.9%), 5 (9.0%), 6 (5.7%), 7 (3.8%), and 8 (3.7%). Additionally, 4.1% have
150 not given birth.
151 Regarding live births, the distribution is as follows: 1 live birth (23.2%), 2 (22.2%), 3 (16.6%), 4 (11.9%), 5
152 (8.5%), 6 (5.6%), 7 (3.5%), and eight or more (3.2%). Notably, 5.3% of participants reported having no live births.
153 The majority, 2,328 (95.0%), reported zero stillbirths, while 95 (3.9%) had one stillbirth, 18 (0.7%) had 2, and 2 (0.08%)
154 had 3, and 1 (0.04%) had 4 stillbirths.
155 Regarding abortions, 1,622 (66.4%) reported having zero abortions in their lifetime. However, a notable portion
156 has experienced abortions: 698 (28.6%) reported 1 abortion, 100 (4.1%) reported 2, 18 (0.7%) reported 3, 5
157 (0.2%) reported 4, and 1 (0.04%) reported 5 **(Fig 1)**.
158 **(Fig1.) Reasons for interruption of ANC care during the war from Pretoria November 2020 to August**
159 **2022 agreement of the Tigray region, Northern Ethiopia**
160 **Fig 3. The line graph shows the reproductive history of study participants of the interruption of ANC during**
161 **the war in Tigray 2023.**
162 Pregnancy intentions among participants
163 The study revealed varied pregnancy intentions among participants. A majority, 1,455 (59.5%), reported
164 were actively trying to become pregnant at the time of conception. However, a significant portion, 738 (30.2%),
165 expressed a desire to wait until later to have children. Additionally, 251 (10.3%) stated that they did not wish to
166 have any more children when they become pregnant. The findings of this study	indicate a diverse range of
167 pregnancy intentions within the study population, highlighting that while many participants were intentionally
168 seeking pregnancy, a notable number preferred to delay or avoid further pregnancies. For more details, see
169 “Table 2”.
170 Antenatal care (ANC) interruption: The prevalence of antenatal care (ANC) interruption was found to be 24.8% (95% CI: [23.1%, 26.5%]),
171 indicating that nearly one in four women encountered difficulties in maintaining consistent ANC. The primary reasons for these interruptions
172 included insecurity (45.3%, 95% CI: [43.1%, 47.5%]), health facility closures (39.8%, 95% CI: [37.6%, 42.0%]), and a lack of supplies and medicine
173 for ANC (33.1%, 95% CI: [31.0%, 35.2%]).
174 The spatial distribution of ANC interruptions across the Tigray region is as follows: Northwestern zone 22.8%, Central zone 29.1%, Eastern zone
175 17.7%, Southern zone 16.7%, Southeastern zone 6.4%, and Mekelle zone 7.3%. However, the Western zone of Tigray was not accessible for our
176 study due to security concerns. For more details, see **Fig 2**.

### 177 Fig 2. Special distribution of ANC interruption across the zone of Tigray Region (n=2444)

178 **Bivariate Analysis: Impact of War on Antenatal Care (ANC) Visits**
179 The study found that 75.2% (1,839, 95% CI: [73.9%, 76.5%]) of participants did not postpone or interrupt their antenatal care (ANC) visits due to
180 the ongoing war, while 24.8% (605, 95% CI: [23.5%, 26.1%]) reported interruptions. Among those with a sick family member in the last two weeks,
181 14.3% (349, 95% CI: [12.9%, 15.7%]) did not postpone their ANC visit, and 7.0% (171, 95% CI: [5.9%, 8.1%]) did.
182 The war's impact was significant, with 96.5% (2,358, 95% CI: [95.4%, 97.6%]) reporting a family member was disabled; of these, 72.7% (1,778,
183 95% CI: [71.0%, 74.4%]) did not postpone their ANC visit, while 23.7% (580, 95% CI: [22.2%, 25.2%]) did. Additionally, 97.0% (2,370, 95% CI:
184 [96.0%, 98.0%]) experienced the loss of a family member, with 73.2% (1,790, 95% CI: [71.5%, 74.9%]) continuing their ANC visits. A small
185 fraction, 1.4% (34, 95% CI: [0.8%, 2.0%]), reported a woman in their household experiencing rape due to the war, with only 1.1% (27, 95% CI:
186 [0.5%, 1.7%]) not postponing ANC visits.
187 Property damage was reported by 53.3% (757, 95% CI: [51.0%, 55.6%]) of participants, with 38.2% (543, 95% CI: [35.9%, 40.5%]) not postponing
188 their ANC visits. Looting affected 89.7% (1,274, 95% CI: [88.5%, 90.9%]), with 66.1% (939, 95% CI: [64.5%, 67.7%]) continuing their visits.
189 Destruction of property was noted by 27.3% (388, 95% CI: [25.6%, 29.0%]) of participants, where 20.3% (288, 95% CI: [18.7%, 21.9%]) did not
190 postpone. Displacement impacted 56.7% (1,386, 95% CI: [54.9%, 58.5%]), with 42.0% (1,027, 95% CI: [40.3%, 43.7%]) not postponing their ANC
191 visits.
192 In terms of distance to health posts, 64.8% (1,583, 95% CI: [63.0%, 66.6%]) reported a round trip of less than 1 hour, while 35.2% (861, 95% CI:
193 [33.4%, 37.0%]) reported longer trips. Those who did not postpone their ANC visits were more likely to live closer to health facilities (49.7% vs.
194 15.1%).
195 Functionality of nearby health facilities was high, with 75.8% (1,303, 95% CI: [74.1%, 77.5%]) reporting nearby health posts as functional and
196 97.0% (2,371, 95% CI: [96.0%, 98.0%]) for health centers/hospitals. Access to public transport was reported by 43.8% (1,071, 95% CI: [41.9%,
197 45.7%]), with those who had not postponed ANC visits slightly more likely to have access (32.8% vs. 11.0%).
198 Regarding electricity supply, 33.9% (829, 95% CI: [32.1%, 35.7%]) reported having it, and those who did not postpone their ANC visits were
199 somewhat more likely to have electricity (26.5% vs. 9.0%). Media ownership was limited, with 12.6% (308, 95% CI: [11.3%, 13.9%]) owning a
200 radio and 16.4% (400, 95% CI: [15.1%, 17.7%]) owning a television. Those who did not postpone their ANC visits were slightly more likely to own
201 a radio (9.6% vs. 3.5%) or a TV (12.6% vs. 4.5%). Mobile phone ownership was similar between both groups, with 46.6% (95% CI: [44.9%,
202 48.3%]) owning a phone.

### 203 Table 2: War and ANC interruption of ANC care during the war to Pretoria November 2020 to August 2022 agreement of the Tigray region

204 **Northern Ethiopia (N=2444)**

**ANC Interruption due to war	ANC Interruption due to war**

**Predictors	Categories	χ^2^**	**P-value	Predictors	Categories	χ^2^**	**P-value**

|  | | **No(n=1839)** | **Yes(n=605)** |  |  |  |  | **No(n=1839)** | **Yes(n=605)** |  | |
| --- | --- | --- | --- | --- | --- | --- | --- | --- | --- | --- | --- |
| **Sick Last 2 Weeks** | Yes | 349 (14.3%) | 171 (7%) | **23.44** | **<0.001** | **HP Distance** | <= 1 hour | 1214(49.7%) | 369(15.1%) | **5.03** | **0.025** |

|  | | No | 1490 (61.0%) | 434 (17.8%) |  |  |  | >= 1 hour | 625(25.6%) | 236(9.7%) |  | |
| --- | --- | --- | --- | --- | --- | --- | --- | --- | --- | --- | --- | --- |
|  |  | Yes | 61(2.5%) | 25(1.0%) |  |  |  | Yes | 994(57.8%) | 309(18.0%) |  |  |
|  | **Disabled War** |  |  |  | **0.89** | **0.345** | **Functional HP** |  |  |  | **1.78** | **0.183** |
|  |  | No | 177(72.7%) | 580(23.7%) |  |  |  | No | 249(14.5%) | 93(5.4%) |  |  |
|  |  | Yes | 49(2.0%) | 25(1.0%) |  |  |  | Yes | 56(37.3%) | 19(12.7%) |  |  |
|  | **Lost Family member in War** |  |  |  | **3.34** | **0.068** | **Functional HC** |  |  |  | **38.51** | **<0.001** |
|  |  | No | 1790(73.2%) | 580(23.7%) |  |  |  | No | 18 (12.0%) | 57 (38.0%) |  |  |
|  | **Rape victim HHs** | Yes  No | 27(1.1%)  1812(74.1%) | 7(0.3%)  598(24.5%) | **0.32** | **0.571** | **Access to transport to HP/HC** | Yes  No | 19 (3.2%)  153 (26.0%) | 90 (15.3%)  326 (55.4%) | **9.03** | **0.003** |
|  | **War Damage** | Yes  No | 490(34.5%)  543(38.2%) | 174(12.2%)  214(15.1%) | 0.76 | 0.383 | **Electricity Supply** | Yes  No | 24 (23.5%)  18 (24.0%) | 78 (76.5%)  57 (76.0%) | 0.942 | 0.005 |
|  |  | Yes | 94(6.6%) | 53(3.7%) |  |  |  | Yes | 24 (23.5%) | 78 (76.5%) |  |  |
|  | **War Looted** | No | 939(66.1%) | 335(23.6%) | 11.38 | 11.38 | **Radio in the HH** | No | 18 (24.0%) | 57 (76.0%) | 0.942 | 0.005 |
|  | **Displaced due to War** | Yes | 1027(42.0%) | 359(14.7%) | 2.26 | 0.138 | **TV in HH** | Yes | 24 (23.5%) | 78 (76.5%) | 0.942 | 0.005 |
|  |  | No | 812(33.2%) | 246(10.1%) |  |  |  | No | 18 (24.0%) | 57 (76.0%) |  |  |
|  |  | Yes | 1033(42.3%) | 388(15.9%) |  |  |  | Yes | 24 (23.5%) | 78 (76.5%) |  |  |
|  | **War Impact in HH** | No | 806(33.0%) | 217(8.9%) | 11.85 | **<0.001** | **Mobile Ownership** | No | 18 (24.0%) | 57 (76.0%) | 0.942 | 0.005 |
| 205 |  |  |  |  |  |  |  |  |  |  |  |  |

206

207

208 **Multivariate Analysis: Parameter Estimates from SLR**
209 The Moran I test indicated significant spatial autocorrelation in ANC interruption (Moran’s I = 0.546, p-value <
210 0.001), indicating the presence of spatial autocorrelation in the dependent variable, validating the need for
211 spatial logistic regression. Incorporating spatial dependencies is crucial, as neglecting them in ordinary logistic
212 regression may result in biased parameter estimates and inaccurate inferences. Model fitness improved
213 substantially with the inclusion of the spatial term, as reflected by an increase in log-likelihood from -1200 to -
214 1050 and a decrease in AIC from 2410 to 2110. The pseudo-R-squared increased from 0.55 to 0.72,
215 demonstrating better explanatory power. Multicollinearity diagnostics showed all Variance Inflation Factors
216 (VIFs) below 3, indicating no serious multicollinearity issues among predictors.
217 The spatial logistic regression model identified several key factors influencing the interruption of antenatal care
218 (ANC) during the war. For every unit increase in distance from the health center, the log-odds of interrupting
219 ANC increased by 0.001 (SE = 0.001, p < 0.001). Geographic differences also played a significant role; women
220 living in the south-east zone had a 0.112 lower log-odds of interrupting ANC compared to those in the north-
221 west zone (SE = 0.05, p = 0.02). Additionally, women in the eastern zone exhibited a 0.16 lower log-odds of
222 interrupting ANC compared to the north-west zone (SE = 0.04, p = 0.001).
223 The number of children under five in the household was another essential factor, with each additional child
224 increasing the log-odds of interrupting ANC by 0.08 (SE = 0.02, p < 0.001). Furthermore, households without a
225 radio had a 0.08939 lower log-odds of interrupting ANC compared to those with a radio (SE = 0.03, p = 0.001).
226 These results suggest that longer distances to health centers, residing in the north-west zone, having more young
227 children, and not owning a radio, were all associated with a greater likelihood of ANC interruption in the war-
228 affected the Tigray region of Ethiopia. In contrast, the analysis for Mekelle indicated an estimate of -0.04, a
229 standard error of 0.07, and a z-value of -0.55, resulting in a p-value greater than 0.05, which shows no
230 significant effect on ANC interruption compared to the north-west zone.
231 In contrast, the estimate for women in Mekelle was -0.038 (SE = 0.069, p > 0.05), indicating no statistically
232 significant difference in ANC interruption compared to the north-west zone. These findings highlight the
233 importance of geographic location, access to information, and household burden in shaping ANC service
234 continuity in war-affected areas of Tigray, Ethiopia. Conversely, the eastern zone showed significantly lower
235 odds of ANC interruption, with an estimate of -0.160 (SE = 0.050, p < 0.05), indicating better adherence to ANC
236 in this zone as seen in Table 3.
237 Table 3: Spatial logistic regression model analysis shows the parameter estimates, standard errors, z-values, and
238 p-values for the predictors of interruption of ANC during the war in Tigray 2023.

| Predictors | Estimate | Std. Error | Z-value | Pr(>\|z\|) |
| --- | --- | --- | --- | --- |
| (Intercept) | 0.164124 | 0.152572 | 1.0757 | 0.28 |
| South-east zone | -0.11876 | 0.050501 | -2.3518 | 0.01** |
| Mekelle zone | -0.0377 | 0.068806 | -0.5479 | 0.58 |
| Eastern zone | -0.16452 | 0.048012 | -3.4266 | 0.001*** |
| Central zone | 0.038805 | 0.041975 | 0.9245 | 0.35 |
| Northwest zone | 0.035293 | 0.046268 | 0.7628 | 0.44 |
| Age | 0.000458 | 0.002425 | 0.189 | 0.85 |
| Number of under-five children | 0.083134 | 0.017295 | 4.8068 | 1.53 |
| Number of Pregnancies | 0.002781 | 0.008414 | 0.3305 | 0.74 |
| Family size | 0.002114 | 0.007472 | 0.2829 | 0.77 |
| Perceived wealth medium | 0.08881 | 0.128912 | 0.6889 | 0.49 |
| Perceived wealth poor | 0.060797 | 0.130133 | 0.4672 | 0.64 |
| Radio ownership | -0.08939 | 0.033425 | -2.6743 | 0.007** |
| Television ownership | -0.02766 | 0.051554 | -0.5365 | 0.59 |
| Mobile phone ownership | -0.04237 | 0.023887 | -1.7739 | 0.07* |

Perceived food Security moderately secured

0.03115936	0.047596	0.6547	0.51

Perceived food Security insecure	0.035233	0.051334	0.6863	0.49

War impacted me	-0.04285	0.022561	-1.8993	0.05*

Distance health post in minutes	-0.00044	0.000266	-1.6706	0.09* Distance of health center in minutes	0.000353	0.000103	3.415	0.001*** Access to transport health Center	-0.00869	0.029711	-0.2925	0.76

239 *p<0.05,	**p<0.01,	***p<0.001

# 240 Discussion

241 The prevalence of antenatal care (ANC) interruption was found to be 605 (24.8%), indicating that nearly one in
242 four women faced challenges in maintaining consistent ANC. The interruption was notably more severe among
243 mothers residing in rural areas (370, 61.2%) compared to semi-urban (51, 8.4%) and urban (184, 30.4%)
244 settings. A significant majority of those affected were host residents (519, 85.8%) rather than internally
245 displaced mothers (86, 14.2%). Additionally, many participants were married (563, 91.1%).
246 Similar studies conducted in Syria in 2014 to 2017	have found that violent events significantly reduce the
247 likelihood of pregnant women seeking and accessing essential healthcare services showed a negative correlation
248 between bombardments and consultations, and ANC visits were reported; the RR was 0.95 (95% CI 0.94–0.97)
249 and 0.95 (95% CI 0.93–0.98), respectively.
250 . The fear and risks associated with ongoing conflicts deter pregnant women from seeking necessary care,
251 increasing the vulnerability of both mothers and their unborn children to potential health complications [22].
252 Research in Yemen highlighted those pregnant women in remote, rural areas encountered substantial barriers to
253 reaching ANC clinics due to infrastructure damage, lack of public transportation, and financial constraints [23].
254 Furthermore, studies in Afghanistan have shown that the Taliban's restrictions on women's movement and
255 access to healthcare during conflicts severely limited pregnant women's ability to attend ANC appointments
256 [24,25]. These challenges were particularly pronounced in rural areas compared to urban settings [26]. This is a
257 critical concern since ANC is a well-established and cost-effective intervention that provides essential care to
258 pregnant women from early pregnancy to delivery [21]
259 The high prevalence of interruptions in antenatal care (ANC) services during conflict situations contradicts the
260 internal resolution of the World Health Organization (WHO, 2012), which stresses the importance of leadership
261 in documenting attacks on health workers, facilities, and patients. [27].
262 The results of the spatial logistic regression analysis in the war-affected Tigray region of Ethiopia revealed
263 several key factors influencing ANC interruptions. For instance, the distance to health centers was a significant
264 factor; for every unit increase in distance (in minutes), the log-odds of discontinuing ANC increased by
265 0.000353 (SE = 0.000103, p < 0.001). This finding aligns with studies conducted in Syria, where ongoing
266 conflict and insecurity significantly disrupted access to ANC services. This was compared by the time taken to
267 reach health facilities both before the war and during the war, even if the distance is constant, the time taken in
268 These two periods were different; this may be due to security reasons, blockage, checkpoints here and there. Women
269 reported facing barriers such as transportation difficulties, fear of checkpoints, and financial constraints when
270 trying to reach health facilities [28, 29]. Additionally, a study in Syria found that women living in besieged or
271 hard-to-reach areas experienced much greater difficulty accessing ANC services due to transportation
272 challenges, security concerns, and a lack of functioning health facilities [30].
273 Living in the north-west zone of the war-affected Tigray region was associated with a higher likelihood of
274 interruption of antenatal care (ANC). This may be the blockage, movement from place to place, or severity of war
275 may be higher in this area and because it is far from Mekelle, the capital city of the Tigray region, which is
276 about 300kms, some of the humanitarian organisations were not reaching this area. This is also similar to studies
277 conducted in Syria, where bombardment and distant villages from the centers interrupted ANC service than
278 those who are near the centers [22]. This is consistent with the fact that in conflict settings, healthcare access is severely
279 were compromised due to the destruction of facilities and the evacuation of medical personnel, leading to increased travel times
280 and reduced service availability [31].
281 Conversely, while distance is a critical factor, other elements such as socio-economic status, education, and
282 Community support also plays a role in healthcare access and continuity in conflict-affected regions
283 [32].
284 Also align with Previous studies, which have consistently highlighted those geographic disparities, often exacerbated by
285 conflict plays a critical role in shaping healthcare utilization. For instance, in conflict-affected regions of
286 Afghanistan, women in remote or insecure areas faced higher barriers to ANC attendance due to disrupted
287 infrastructure and security concerns, a pattern that resonates with the higher likelihood of ANC interruption
288 [33]. Additionally, households with a greater number of young children were more prone to discontinuation,
289 as were those who did not own a radio. These findings underscore the unique challenges faced by pregnant
290 women in war-affected regions, highlighting the need for improved access to health facilities, addressing
291 transportation and security concerns, and implementing targeted interventions to support households with
292 multiple young children.
293 Research from Yemen and the Democratic Republic of the Congo further illustrates the severe Impact of armed
294 conflicts and civil wars on maternal healthcare access in sub-Saharan Africa. In Yemen, the escalation of the
295 civil war led to the closure of many health facilities, significantly hindering pregnant women’s ability to receive
296 regular ANC [34, 35]. Similarly, findings from the Democratic Republic of the Congo indicate that armed
297 conflicts and displacement forced many pregnant women to miss critical ANC visits, resulting in poorer
298 maternal and child health outcomes [36,37,38]. Pregnant women displaced to rural areas in the DRC exhibited
299 much lower ANC attendance rates compared to those who remained in or fled to urban centers [36]. Overall, the
300 effects of civil wars on sub-Saharan Africa have severely compromised maternal and child health services [39].
301 The spatial logistic regression analysis highlighted distance to health centers as a significant factor; for every
302 unit increase in distance (in minutes), the log-odds of discontinuing ANC increased by 0.001 (SE = 0.001, p <
303 0.001). Studies from Yemen reported substantial barriers for pregnant women in remote, rural areas trying to
304 access ANC clinics, primarily due to infrastructure damage, lack of public transportation, and financial
305 constraints [40, 41]. Studies from North East Ethiopia also showed that due to facility damage and medical
306 personnel evacuation, access to healthcare is seriously jeopardized in crises, resulting in more extended travel
307 times and fewer services available[42]. Another study on the quality of health care service in a conflict setting showed
308 that healthcare disruption in armed conflict creates significant obstacles to the provision of healthcare, such as
309 medical supply shortages and access-hindering security threats [43]. On the other hand, although distance is an
310 important considerations, socioeconomic position, education, and community support are also important factors
311 that affect healthcare continuity and access in areas affected by violence(44).
312 Geographic zones also played a critical role; women living in the south-east zone had a 0.11lower log-odds of
313 discontinuing ANC compared to those in the north-west zone (SE = 0.05, p = 0.01). Similarly, women in the
314 eastern zone exhibited a 0.16 lower log-odds of discontinuing ANC compared to the north-west zone (SE =
315 0.04, p = 0.001). Thus, living in the north-west zone was associated with a higher likelihood of ANC
316 discontinuation in the Tigray region. This finding mirrors those from the Eastern Mediterranean region, where
317 conflicts have disrupted health service utilization, especially in rural areas [45]. A study in the Democratic
318 Republic of the Congo noted that pregnant women displaced to rural areas had significantly lower ANC
319 centres, per cent of the population than those remaining in or fleeing to urban centers [46, 47]. Furthermore, research in
320 Afghanistan showed that the Taliban's restrictions on women's movement disproportionately affected rural
321 women, leading to higher rates of missed ANC appointments compared to their urban counterparts [48, 49, 47,
322 50,51].
323 These findings underscore the critical importance of addressing the unique challenges faced by pregnant women
324 in war-affected regions, particularly those living in remote, rural areas with limited access to health facilities.
325 Enhancing transportation, infrastructure, and security in these areas is essential for improving maternity
326 healthcare utilization and outcomes.
327 Broader patterns in conflict-affected settings reveal that the uneven distribution of conflict intensity and damage
328 to health infrastructure across different zones has led to varying impacts on pregnant women's ability to receive
329 antenatal care (ANC). The findings from this study in Tigray align with another analysis indicating that, as of
330 June 2021, only 27.5% of hospitals, 17.5% of health centers, 11% of ambulances, and none of the 712 health
331 posts were functional [52].
332 While the specific reasons for the higher ANC interruption in the north-west zone of Tigray were not detailed,
333 further contextual analysis is needed to understand the underlying drivers of this geographic disparity.
334 Additionally, the number of children under five in the household significantly influenced ANC continuity; for
335 each additional child, the log-odds of discontinuing ANC increased by 0.083134 (SE = 0.017295, p < 0.001).
336 Parity, and the interruption of ANC visits were positively correlated, according to demographic and health surveys
337 were conducted in eight Sub-Saharan African (SSA) nations, especially for women with five or more children.
338 According to our research, mothers who have more children are also more likely to stop receiving ANC care.
339 This implies that moms' capacity to prioritize their own care may be hampered by the responsibilities of raising
340 several children. Regular ANC visits seem to be overshadowed by the strain of caring duties, which could
341 endanger the health of mothers and children [53]. Another study conducted in Tanzania also found that women with
342 higher parity often relies on their previous pregnancy experiences, leading to a tendency to underutilize ANC services. This
343 reliance on past experiences may create a false sense of security, causing these women to deem ANC less critical [54].
344 However, the practical challenges of managing several young children further complicate their ability to seek necessary
345 prenatal care, reinforcing the findings of the SSA survey and the present studies. Additionally, research from Liverpool
346 studied the unique challenges faced by women experiencing multiple pregnancies. This study emphasized that increased
347 anxiety and practical obligations hinder their capacity to prioritize ANC visits [55]. All those findings underscore as the
348 number of children increased, which increases the interruption of care.This showed that mothers are overwhelmed by the
349 care of children thus our ANC care contacts need to be tailored to support individualized care strategies to address
350 the specific needs of women, ensuring that they receive adequate prenatal care.
351 This trend may reflect the additional caregiving responsibilities that pregnant mothers with multiple young
352 children face, which can hinder their ability to prioritize their own ANC visits.
353 This may be severe in the condition of conflict where health facilities were closed due to war, health personnel
354 displaced as shown by community-based study in armed conflict in Tigray showed that only 36.5%,
355 respondents reported the use of optimal antenatal care (ANC) [56].
356 Moreover, household radio ownership emerged as an important factor; households without a radio had a
357 0.08939 lower log-odds of discontinuing ANC compared to those with a radio (SE = 0.033, p = 0.007). This
358 suggests that access to media and information sources, such as radio, can positively influence ANC utilization,
359 as mothers who receive ANC-related information are more likely to act on it. A study of antenatal care and
360 skilled births in the fragile and conflict-affected situation of Burundi indicated that women who engage with
361 media, including radio, are more likely to utilize healthcare services which is similar to the present study mothers who
362 own radio did not interrupt ANC visits; this may be the information disseminated through the radio may be
363 indicating where the services is available and also information about security issues about war may be
364 disseminated so as mothers know when to visit health facilities and when not to visit health facilities [57].
365 Another study conducted on Antenatal care utilization and determinants in fragile conflict-affected
366 situations in northern Ethiopia showed that radio can foster community discussions around maternal health,
367 increasing awareness and reducing stigma associated with seeking ANC in conflict zones, thus in conflict
368 affecting zones radio may be an appropriate massage or information transmission methods that can support
369 health services utilization of communities in conflict affecting situations [58,59].
370 Another study conducted in the political unrest in Nepal showed that in areas where physical access to healthcare is
371 limited, radio can bridge the gap by informing women about available services and how to access them,
372 potentially increasing ANC visits, this is another evidence that supports the present study, thus owning a radio in
373 such conflict situations may get information that supports where and when to visit health facilities so that you get
374 health services [59, 60, 61]
375 The Tigray study offers a more granular, zone-level analysis that complements zonal-level findings from other
376 conflict-affected contexts, highlighting the importance of understanding subnational variations in healthcare
377 access during conflicts. Further research to unpack the underlying drivers of geographic disparities in ANC
378 interruption in Tigray would be invaluable for informing more targeted interventions.

# 379 Strength and limitation of the study

380 Spatial modeling offers significant strengths in studying antenatal care interruption, particularly through its
381 ability to provide clear visual representations of geographic patterns. This clarity helps stakeholders identify
382 areas with high rates of interruption, facilitating a better understanding of the spatial dynamics of healthcare
383 access. Furthermore, these models allow researchers to incorporate a range of socio-economic, environmental,
384 and conflict-related variables, leading to a comprehensive understanding of the factors influencing antenatal
385 care. By pinpointing hotspots, health authorities can strategically allocate resources and design targeted
386 interventions, enhancing the potential for improved access and outcomes in affected regions. Additionally,
387 spatial modelling enables tracking changes over time, revealing trends related to ongoing conflicts or
388 recovery efforts, which is crucial for understanding the dynamics of healthcare access in fluctuating
389 environments. The insights gained from these models can also inform policymakers about the specific needs of
390 affected populations, guiding the development of effective health service delivery strategies.
391 However, spatial modelling also faces notable limitations. In war-torn regions, the quality and availability of
392 data can be compromised, leading to incomplete data, like the western zone of the Tigray region, not included because of
393 security reasons or biases that undermine the reliability of the models. Complex health outcomes are influenced
394 by multiple interdependent factors that may not be fully captured, limiting the explanatory power of the models.
395 Moreover, the assumptions and simplifications inherent in spatial modelling can oversimplify the intricate
396 realities of healthcare access, potentially resulting in misinterpretations of the data. Rapid changes in conflict
397 situations may outperform the data used in models, diminishing their relevance over time. Lastly, findings
398 derived from specific regions may not be generalizable to other contexts, which restricts the broader
399 applicability of results and may hinder the development of universal strategies for improving antenatal care
400 access

# 401 Conclusion and recommendations

402 The study identified the high prevalence of antenatal care (ANC) interruption, Key factors associated with the
403 likelihood of interruption of ANC included greater distance from health centers, residing in the north-west zone
404 compared to the south-east and eastern zones, having a higher number of children under five in the household,
405 and household ownership of a radio.
406 To address these findings, several strategies are recommended. First, improving access to health centers could
407 be achieved by establishing more community-based ANC services or implementing transportation assistance
408 programs. Second, targeted interventions are needed for high -risk north-west zone to specifically address the
409 elevated rates of ANC interruption in this region. Third, providing additional resources and support, such as
410 childcare assistance or conditional cash transfers could benefit households with multiple young children,
411 helping to alleviate caregiving burdens.
412 Promoting the use of radio and other mass media channels is also crucial for disseminating information and
413 encouraging ANC attendance, particularly in households with a radio. Additionally, further research is essential
414 to unpack the underlying drivers of geographic disparities in ANC interruption in Tigray, which would inform
415 more targeted and effective interventions. By addressing these factors, it may be possible to enhance the
416 continuity of antenatal care and ultimately improve maternal and child health outcomes in the Tigray regio

417

418 The findings indicate significant interruptions in antenatal care (ANC) in Tigray, Driven by geographic
419 disparities, household dynamics, and access to information. Specifically, the increased log-odds of interruptions
420 with additional travel time highlights the urgent need for improved infrastructure and security. The lower log-
421 odds of interruption in the southeast and eastern zones suggest that targeted interventions could be beneficial in
422 areas most affected. Furthermore, the association between household dynamics and ANC interruptions
423 emphasizes the importance of enhancing mass media health information dissemination to improve access to
424 care. Therefore, further research on geographic disparities is essential to inform tailored strategies for improving
425 ANC services in the region.

# 426 Ethical considerations

427 Ethical clearance was approved by the Tigray Health Research Institute review board with reference number
428 (THRI 4031/1099/15). A permission letter was obtained from the Tigray regional health bureau. Oral consent
429 and volunteer participation was obtained from study participants after fully communicating the benefits and
430 risks of participation in the study with a right to discontinue participation at any interview stage. Data
431 confidentiality was maintained throughout the research process. In conducting our research with vulnerable
432 populations in conflict zones, we prioritize several ethical considerations to ensure the integrity and safety of
433 the study. Informed consent is fundamental; we ensure that participants fully understand the study's purpose,
434 risks and benefits by simplifying the language to Tigrigna and providing culturally relevant information. Safety
435 and security are paramount, so we assess potential risks associated with participation and implement measures
436 to mitigate them, safeguarding participants, researchers, and the community. We also maintain confidentiality
437 by protecting participants' identities and personal information through anonymization techniques and secure
438 data storage, particularly in sensitive contexts. Our approach is guided by cultural sensitivity, recognizing and
439 respecting local norms and values, and actively engaging with communities to ensure our methods are
440 appropriate. We strive for beneficence and non-maleficence, aiming to maximize benefits while minimizing
441 harm, considering how our research outcomes can positively impact the community. Community engagement is
442 crucial; we involve local stakeholders and organizations, such as health facility workers, in the data collection
443 process to enhance the relevance and acceptance of our research. Additionally, we plan for post-study benefits,
444 ensuring that findings are shared with participants and the community so they can directly benefit from the
445 results. Finally, we have obtained approval from an ethics review board, acknowledging the unique challenges
446 posed by conflict situations and reinforcing our commitment to ethical research practices.

# 447 Acknowledgements

448 We extend our heartfelt gratitude to the Tigray Health Bureau (THB) for leading this integrated research and
449 mobilizing technical staff for data collection. Lastly, we thank the participants, as well as the data collectors and
450 supervisors, particularly those from the Mekelle University College of Health Sciences and Tigray Health
451 Research Institute (THRI), for their commitment and integrity in generating quality data.

# 452 Conflicts of interests

453 In the research on antenatal care interruption in Tigray, we maintain a strong commitment to avoiding conflicts
454 of interest. We ensure that our funding sources do not create biases, as we do not have any conflicts with
455 organizations that might have vested interests in the outcomes, such as government agencies or NGOs with
456 specific agendas. Our collaborative relationships with local health authorities and NGOs are structured to
457 prevent any conflicts, as these entities do not have a stake in the research findings that could influence our
458 work. Additionally, the researchers do not have personal ties to the region or its healthcare systems that could
459 compromise the interpretation of data or reporting results. We are also vigilant against publication bias,
460 ensuring that we do not face conflicts that would pressure us to publish only positive outcomes aligned with
461 funding interests, which could distort the integrity of our research. Ethical considerations are paramount; we
462 adhere to rigorous ethical guidelines to address the unique challenges of working with vulnerable populations in
463 conflict zones, recognizing the dilemmas that may arise. To mitigate potential conflicts, we prioritize
464 transparency by disclosing all funding sources and potential conflicts in our publications. We are committed to
465 an independent review process, welcoming input from independent experts in study design and data analysis to
466 ensure objectivity. Finally, we uphold ethical oversight by obtaining approval from relevant review boards
467 Tigray health research institute (THRI) as mentioned above to safeguard participant welfare, reinforcing our
468 dedication to conducting responsible and ethical research.

### 469 Authors’ Contributions:

470 A. A., M. A., H. G., and B. A., conceived and designed the experiments and analyzed and write up the report
471 H. G., F. H., T. W., A. A., A. A. M., Z. H. K., L. M., M. K. C., R. S., M. A., H. K., T. H., F. G., A. A., K. D.,
472 M. T., M. W., A. G., A. A. D., G. G., A. H., R. E., T. B., A. G., T. T., A. A., A. M. B. performed the
473 experiments and approved and revised the final manuscript

# 474 Reference

475 1. WHO, UNICEF, UNFPA and TheWorld Bank. Trends inMaternalMortality: 2000 to 2017WHO.
476 Geneva:WHO (2019).
477 2. Tunçalp Ö, Pena-Rosas JP, Lawrie T, et al. WHO Recommendations on antenatal care for a positive
478 pregnancy experience-going beyond survival. Bjog. 2017;124(6):860-862.
479 3. O’hare BAM, David PS. First do no harm: the impact of recent armed conflict on maternal and child health

480	in Sub-Saharan Africa. J R Soc Med. (2007) 100:564–70. doi: 10.1258/jrsm.100.12.564

481 4. DeJong J, Ghattas H, Bashour H, Mourtada R, Akiki C, Reese_MastersonA. Reproductive, maternal,
482 neonatal and child health in conflict: a case study on Syria using countdown indicators. BMJ Global Health.
483 (2017) 2:e000302. doi: 10.1136/bmjgh-2017-000302
484 5. Akseer N, Wright J, Tasic H, Everett K, Scudder E, Amsalu R, et al. Women, children and adolescents in
485 conflict countries: an assessment of inequalities in intervention coverage and survival BMJ Global Health.
486 (2020) 5:e002214. doi: 10.1136/bmjgh-2019-002214
487 6. Mullany LC, Lee CI, Yone L, Paw P, Oo EKS, Maung C, et al. Access to essential maternal health
488 interventions and human rights violations among vulnerable communities in Eastern Burma. PLoS Med.
489 (2008)5: e242. doi: 10.1371/journal.pmed.0050242
490 7. Central Statistical Agency (CSA) [Ethiopia] and ICF. Ethiopia Demographic and Health Survey 2016.
491 Addis Ababa, Ethiopia, and Rockville, Maryland, USA: CSA and ICF. 2016

492

493 8. Abay ST, Gebre-Egziabher AG. Status and associated factors of birth registration in selected districts of Tigray region, Ethiopia.
494 BMC international health and human rights. 2020 Dec;20:1-0.
495 9. Godefay H, Byass P, Graham WJ, Kinsman J, Mulugeta A. Risk factors for maternal mortality in rural
496 Tigray, northern Ethiopia: a case-control study. PloS one. 2015 Dec 17;10(12):e0144975.
497 10. Legesse AY, Teka Tseghay H, Abraha HE, Fisseha G, Ebrahim MM, Tsadik M, Berhe B, Gebrekurstos G,
498 Ayele B, Gebremeskel T, Gebremariam T. Maternal mortality during war time in Tigray, Ethiopia: A
499 community‐based	study.	BJOG:	An	International	Journal	of	Obstetrics	&	Gynaecology.	2024
500 May;131(6):786-94.
501 11. Namasivayam, Amrita, González, Pedro Arcos, Delgado, Rafael Castro, Chi,Primus Che. The effect of
502 armed conflict on the utilization of maternal health services in Uganda: a population-based study. PLoS
503 Curr Disast. (2017) 9:1689–98. doi: 10.1371/currents.dis.557b987d6519d8c7c96f2006ed3c271a
504 12. Kabakian-Khasholian, Tamar, Shayboub, Rawan, El-Kak, Faysal. Seeking maternal care at times of
505 conflict:	the	case	of	Lebanon.	Health	Care	Women	Int.	(2013)	34:352–62.	doi:
506 10.1080/07399332.2012.736570
507 13. Kabakian-Khasholian, Tamar, Shayboub, Rawan, El-Kak, Faysal. Seeking maternal care at times of
508 conflict:	the	case	of	Lebanon.	Health	Care	Women	Int.	(2013)	34:352–62.	doi:
509 10.1080/07399332.2012.736570
510 14. Chi PC, Bulage P, Urdal H, Sundby J. Perceptions of the effects of armed conflict on maternal and
511 reproductive health services and outcomes inBurundi and Northern Uganda: a qualitative study. BMC Int
512 Health Hum Rights. (2015) 15:7. doi: 10.1186/s12914-015-0045-z
513 15. Ethiopian Public Health Institute (EPHI) [Ethiopia] and ICF. Ethiopia Mini Demographic and Health
514 Survey 2019: Final Report. Rockville, Maryland, USA: EPHI and ICF. 2021
515 16. Gesesew H, Berhane K, Siraj ES, et al. The impact of war on the health system of the Tigray region in
516 Ethiopia: an assessment. BMJ Global Health 2021;6: e007328. doi:10.1136/ bmjgh-2021-007328
517 17. Alemayehu M, Demoz K, Yahya M, Teamir A, Asmelash A, Gebrekurstos G, et al. Does integration of
518 health survey bring cost effectiveness into evidence generation in post conflict settings of Tigray, Ethiopia?.
519 East Afr J Health Sci. 2024;6(1):798-808. doi: [10.71624/p8bzch81](https://doi.org/10.71624/p8bzch81)
520 18. Paciorek CJ. Computational techniques for spatial logistic regression with large data sets. Comput Stat Data

521	Anal. 2007;51(8):3631-53. doi:10.1016/j.csda.2006.11.008.

522	19. Baddeley A, Berman M, Fisher N, et al. Spatial logistic regression and change-of-support in Poisson point

523	processes. Electron J Stat. 2010; 4:1151-201. doi:10.1214/10-ejs581.

524 20. Bihrmann K, Toft N, Nielsen SS, et al. Spatial correlation in Bayesian logistic regression with
525 misclassification. Spatial Spatio-temporal Epidemiol. 2014; 9:1-12. doi:10.1016/j.sste.2014.02.002
526 21. World Health Organization. WHO recommendations on antenatal care for a positive pregnancy experience:
527 summary: highlights and key messages from the World Health Organization's 2016 global recommendations
528 for routine antenatal care. World Health Organization; 2018.
529 22. Ekzayez A, Alhaj Ahmad Y, Alhaleb H, Checchi F. The impact of armed conflict on utilisation of health
530 services in north-west Syria: an observational study. Conflict and health. 2021 Dec; 15:1-0.
531 23. Stanikzai MH, Tawfiq E, Jafari M, Wasiq AW, Seddiq MK, Currie S, Sayam H, Baray AH, Saeedzai SA.
532 Contents of antenatal care services in Afghanistan: findings from the national health survey 2018. BMC
533 Public Health. 2023 Dec 11;23(1):2469.
534 24. Glass N, Jalalzai R, Spiegel P, Rubenstein L. The crisis of maternal and child health in Afghanistan.
535 Conflict and health. 2023 Jun 12;17(1):28.
536 25. Saleem SM, Shoib S, Dazhamyar AR, Chandradasa M. Afghanistan: decades of collective trauma, ongoing
537 humanitarian crises, Taliban rulers, and mental health of the displaced population. Asian journal of
538 psychiatry. 2021 Nov 1; 65:102854.
539 26. Zhang T, He Q, Richardson S, Tang K. Does armed conflict lead to lower prevalence of maternal health-
540 seeking behaviours: theoretical and empirical research based on 55 683 women in armed conflict settings.
541 BMJ Global Health. 2023 Aug 1;8(8): e012023.
542 27. World Health Organization. WHO’s response, and role as the health cluster lead, in meeting the growing
543 demands of health in humanitarian emergencies [Internet]. Sixty-Fifth World Health Assembly. 2012
544 May:21-6.
545 28. Mourtada R, Bashour H, Houben F. A qualitative study exploring barriers to adequate uptake of antenatal
546 care in pre-conflict Syria: low-cost interventions are needed to address disparities in antenatal care.
547 Contraception and Reproductive Medicine. 2021 Jun 1;6(1):17.
548 29. Basha S, Socarras A, Akhter MW, Hamze M, Albaik A, Hussein I, Tarakji A, Hamadeh M, Loutfi R,
549 Kewara M, Alahdab F. Protracted armed conflict and maternal health: a scoping review of literature and a
550 retrospective analysis of primary data from northwest Syria. BMJ Global Health. 2022 Aug 1;7(8):
551 e008001.
552 30. Nabulsi D, Abou Saad M, Ismail H, Doumit MA, El-Jamil F, Kobeissi L, Fouad FM. Minimum initial
553 service package (MISP) for sexual and reproductive health for women in a displacement setting: a narrative
554 review on the Syrian refugee crisis in Lebanon. Reproductive Health. 2021 Dec; 18:1-3.
555 31. Mesfin, B., Demise, A. M., Shiferaw, M., Gebreegziabher, F., & Girmaw, F. (2023). The Effect of Armed
556 Conflict on Treatment Interruption, Its Outcome and Associated Factors among Chronic Disease Patients in
557 North	East,	Amhara,	Ethiopia,	2022. Patient	Related	Outcome	Measures, 14,	243–251.
558 <https://doi.org/10.2147/prom.s388426>
559 32. Tessema, Z. T., & Akalu, T. Y. (2020). Spatial Pattern and Associated Factors of ANC Visits in Ethiopia:
560 Spatial and Multilevel Modeling of Ethiopian Demographic Health Survey Data. Advances in Preventive
561 Medicine, 2020, 4676591. <https://doi.org/10.1155/2020/4676591>.
562 33. Akseer, N., et al. (2020). Progress in maternal and child health: How has Afghanistan fared in the last decade? The
563 Lancet Global Health, 8(3), e351–e360.
564 34. Qirbi N, Ismail SA. Health system functionality in a low-income country in the midst of conflict: the case of
565 Yemen. Health policy and planning. 2017 Jul 1;32(6):911-22.
566 35. Hyzam D, Zou M, Boah M, Saeed A, Li C, Pan S, Zhai J, Wu LJ. Health information and health-seeking
567 behaviour in Yemen: perspectives of health leaders, midwives and mothers in two rural areas of Yemen.
568 BMC Pregnancy and Childbirth. 2020 Dec; 20:1-2.
569 36. Stamidis K, Bologna L, Losey L. CORE Group Polio Project (CGPP) Final Evaluation Report 2017.
570 37. Zhang T, Qi X, He Q, Hee J, Takesue R, Yan Y, Tang K. The effects of conflicts and self-reported
571 insecurity on maternal healthcare utilisation and children health outcomes in the democratic republic of
572 congo (Drc). InHealthcare 2021 Jul 3 (Vol. 9, No. 7, p. 842). MDPI.
573 38. Bwirire D, Roosen I, De Vries N, Letschert R, Ntabe Namegabe E, Crutzen R. Maternal Health Care
574 Service Utilization in the Post-Conflict Democratic Republic of Congo: An Analysis of Health Inequalities
575 over Time. InHealthcare 2023 Oct 31 (Vol. 11, No. 21, p. 2871). MDPI.
576 39. Ormhaug CM. Effects of civil war on maternal and child health care in sub-Saharan Africa 2014
577 40. Ziegler BR. Pregnancy in Peril: the impact of conflict on antenatal care and skilled birth attendant utilization
578 in the Democratic Republic of the Congo and Burundi (Doctoral dissertation, The University of Western
579 Ontario (Canada))2020.
580 41. Yaya S, Uthman OA, Bishwajit G, Ekholuenetale M. Maternal health care service utilization in post-war
581 Liberia: analysis of nationally representative cross-sectional household surveys. BMC Public Health. 2019
582 Dec;19:1-2
583 42. Asah, M., & Moses, A. L. (2025). The Effects of Armed Conflict on Healthcare in Nigeria – A Scoping
584 Review. International	Journal	of	Research	and	Scientific	Innovation, XI(XV),	883–900.
585 https://doi.org/10.51244/ijrsi.2024.11150067p
586 43. Ikpongifono, U. (2024). Enhancing Healthcare Access in Conflict Zones: Identifying Challenges and
587 Proposing Solutions. Public Health Open Access, 8(1). https://doi.org/10.23880/phoa-16000286
588 44. Mavole, J. (2023). Protection and Maintenance of Healthcare Services for Civilians’ Well-Being in Conflict
589 Affected Areas: Comparative Analysis of The African Situation. European Journal of Health Sciences, 9(2),
590 1–17. https://doi.org/10.47672/ejhs.1507
591 45. Grainger, C.G., Gorter, A.C., Al-Kobati, E. et al. Providing safe motherhood services to underserved and
592 neglected populations in Yemen: the case for vouchers. Int J Humanitarian Action **2**, 6 (2017).
593 <https://doi.org/10.1186/s41018-017-0021-4>
594 46. Sheline A. The Yemen War in Numbers: Saudi Escalation and US Complicity. Quincy Institute of US
595 Complicity. 2022 Mar 21
596 47. GOVERNMENT ACCOUNTABILITY OFFICE WASHINGTON DC. Uncertain Political and Security
597 Situation Challenges US Efforts to Implement a Comprehensive Strategy in Yemen. 2012
598 48. 48. Karakır İA. Ongoing conflict in Yemen: A proxy war Tesam Akademi Dergisi. 2018;5(2):121-49.
599 49. Krug EG, Mercy JA, Dahlberg LL, Zwi AB. The world report on violence and health. The lancet. 2002 Oct

600	5;360(9339):1083-8.

601 50. Alibhai KM, Ziegler BR, Meddings L, Batung E, Luginaah I. Factors impacting antenatal care utilization: a
602 systematic review of 37 fragile and conflict-affected situations. Confl Health. 2022 Jun 11;16(1):33. doi:
603 10.1186/s13031-022-00459-9. PMID: 35690840; PMCID: PMC9188725
604 51. Wake SK, Botore A, Mohammed A, Gemede K, Bariso M, Gerema U. Disparities in Antenatal Care Visits
605 between Urban and Rural Ethiopian Women. J Pregnancy. 2023 Sep 27; 2023:9031344. doi:
606 10.1155/2023/9031344. PMID: 37799709; PMCID: PMC10550413.
607 52. Ahmadi A. Assessing Participation in Women's Development Projects in Afghanistan 2017
608 53. Saqib M. Afganistan’da Devlete Yapılanan Kusurlar: Afganistan’ın 2004 Anayasasına Göre Yerel
609 Seçimlerinin Kritik Bir Analizi (Doctoral dissertation, Marmara Universitesi (Turkey)). 2022
610 54. Gesesew H, Berhane K, Siraj ES, et al. The impact of war on the health system of the Tigray region in
611 Ethiopia: an assessment. BMJ Global Health 2021;6: e007328. doi:10.1136/ bmjgh-2021-007328
612 55. Odusina, E. K., Ahinkorah, B. O., Ameyaw, E. K., Seidu, A.-A., Seidu, A.-A., Budu, E., Zegeye, B., &
613 Yaya, S. (2021). Noncompliance with the WHO’s Recommended Eight Antenatal Care Visits among
614 Pregnant Women in Sub-Saharan Africa: A Multilevel Analysis. BioMed Research International, 2021,
615 6696829. <https://doi.org/10.1155/2021/6696829>
616 56. Owen, D. J., Wood, L., & Neilson, J. P. (2004). Antenatal care for women with multiple pregnancies: the
617 Liverpool	approach.	[Clinical	Obstetrics	and	Gynecology, 47(1),	263–271.](https://doi.org/10.1097/00003081-200403000-00026)
618 <https://doi.org/10.1097/00003081-200403000-00026>
619 57. Gupta, S., Yamada, G., Mpembeni, R., Frumence, G., Callaghan-Koru, J. A., Stevenson, R., Brandes, N., &
620 Baqui, A. H. (2014). Factors Associated with Four or More Antenatal Care Visits and Its Decline among
621 Pregnant	Women	in	Tanzania	between	1999	and	2010.	[PLOS	ONE, 9(7).](https://doi.org/10.1371/JOURNAL.PONE.0101893)
622 <https://doi.org/10.1371/JOURNAL.PONE.0101893>
623 58. Tsadik M, Teka H, Gebremichael MW, Gebretnsae H, Yemane A, Fisseha G, Gebregzabher T, Buruh G,
624 Gebresellasie F, Tekie M, Kiros G. Armed conflict and maternal health service utilization in Ethiopia’s
625 Tigray Region: a community-based survey. BMC public health. 2024 Oct 16;24(1):2840.
626 59. Ziegler, B. R., Kansanga, M. M., Sano, Y., Sano, Y., Kangmennaang, J., Kpienbaareh, D., & Luginaah, I.
627 (2021). Antenatal care and skilled birth in the fragile and conflict-affected situation of Burundi International
628 Journal of Health Planning and Management, 36(4), 1081–1106. https://doi.org/10.1002/HPM.3157
629 60. Gedef, G. M., Girma, B., Tibebu, N. S., Gashaw, A., & Andualem, F. (2023). Antenatal care utilization and
630 determinants in fragile and conflict-affected situations in Sekota zuria district, northern Ethiopia, 2022: a
631 community-based cross-sectional study. Midwifery. <https://doi.org/10.1016/j.midw.2023.103906>
632 61. Price, J. I., & Bohara, A. K. (2013). Maternal health care amid political unrest: the effect of armed conflict
633 on	antenatal	care	utilization	in	Nepal. Health	Policy	and	Planning, 28(3),	309–319.
634 https://doi.org/10.1093/HEAPOL/CZS062

Revised manuscript with track change

**Formatted:** Section start: Continuous

1	***Spatial patterns and predictors of antenatal care interruption***

2	***in war-torn Tigray, northern Ethiopia: spatial ~~modelling~~***

3	modelling ***approach***

**Formatted:** Font: (Default) Calibri Light, 18 pt, English (United Kingdom), Ligatures: Standard + Contextual

4

**Formatted:** Line spacing: 1.5 lines

5 Assefa Ayalew Gebrselassie *^1^ ,Mussie Alemayehu^1,4^, Haftu Gebrehiwot^4^ , Brhane Ayele^4^,
6 Hailay Gebretnsae^4^ ,Ferehiwot Hailemariam^1^, Tsegay Wellay^1^, Adhena Ayalew^1^, Araya Abrha
7 Medhanyie^1,6^, Znabu Hadush Kahsay^1^, Liya Mamo^1^, Mebrahtu Kalayu Chekole^1^, Reda Shamie^1^,
8 Mohammedtahir Yahya^2,3^ , Melaku Abraha^3^, Hayelom Kahsay^4^, Tsegay Hadgu^4^, Fana
9 Gebreslassie^4^, Asfawosen Aregay^4^ , Kiros Demoz ^4^ , Mulugeta Tilahun^4^, Mulugeta Woldu^4^,
10 Ataklti Gebrtsadik^4^, Abraham Aregay Desta^4^, Gebrehaweria Gebrekuristos^5^, Amanuel Haile^5^,
11 Rieye Esayas^5^,Tsegay Berihu^5^,Abrham Gebrelibanos^6^, Tadele Tesfean^6^, Ashenafi Asmelash^7^ ,
12 Afework Mulugeta Bezabh^1^

13

14 ^1^ School of Public Health, Mekelle University, College of Health Sciences, Mekelle, Ethiopia.
15 ^2^ Department of Obstetrics and Gynecology, School of Medicine, Mekelle University, College of
16 Health Sciences, Mekelle, Ethiopia.
17 ^3^ Mekelle Hamlin Fistula Center, Mekelle, Tigray Ethiopia
18 ^4^ Tigray Health Research Institute, Mekelle, Tigray Ethiopia
19 ^5^ Tigray Health Bureau, Mekelle, Tigray Ethiopia
20 ^6^ MARCH research center, College health Sciences, Mekelle, Tigray, Ethiopia
21 ^7^ Mums for Mums, Mekelle, Tigray, Ethiopia

22

**Formatted:** Line spacing: 1.5 lines

23 *Corresponding author
24 Email: [aayalew120@gmail.com](mailto:aayalew120@gmail.com) (AA)

25

26

27

28

**Formatted:** Line spacing: 1.5 lines

29	**Abstract**

## 30 Introduction

31 Antenatal care (ANC) is crucial for maternal health, yet its provision has been severely disrupted
32 in conflict-affected Tigray, Ethiopia. This study ~~analyzes the spatial patterns and predictors of~~
33 ~~ANC interruption using a community-based approach~~ uses a community-based approach to analyse the
34 spatial patterns and predictors of ANC interruption in conflict affected, Tigray region, northern Ethiopia.

35

36	.

## 37 ~~Materials &~~Methods and Materials

38 A ~~multistage~~ cross-sectional design was employed, to select 2,444 eligible women from 13,915
39 women of reproductive age across from six zones and 57 clusters. Multistage cluster sampling
40 was used to approach the study participants. Data were collected through structured
41 questionnaires, and geographic coordinates were obtained for spatial geo-points. A spatial
42 logistic regression model was used to assess~~ed~~ the effects of geographic and socio-demographic
43 factors on ANC interruptions, accounting for spatial dependencies.

44	**Results**

45 ~~In this study, t~~The prevalence of ANC interruption was24.8% (n=605, 95% CI:23.1%, 26.5%).
46 Each additional ~~minute~~ minutes of travel increased the log-odds of interruption by 0.000353 (SE

47	= 0.000103, p < 0.001), ~~highlighting accessibility challenges due to security checkpoints and~~

48 ~~conflict-related dangers.~~ Geographic zone significantly affected ANC interruptions, with women
49 in the southeast and eastern zones experiencing lower log-odds compared to the northwest zone.
50 Additionally, having more ~~children~~ under five children raised the log-odds of ANC interruption
51 by 0.083134 (SE = 0.017295, p < 0.001). Households without a radio had a 0.08939 lower log-
52 odds of ANC interruption (SE = 0.033425, p = 0.007489), indicating the importance of access to
53 information.

**Formatted:** Line spacing: 1.5 lines

54	**Conclusions**

55 The findings revealed significant ANC interruption ~~significant ANC interruptions~~ in Tigray,
56 influenced by geographic disparities, household dynamics, and information access. Improving
57 infrastructure and security, implementing targeted interventions, enhancing mass media health
58 information dissemination, and further research on geographic disparities are essential.
59 **Key words**
60 ANC, interruption, spatial logistic, war, Tigray

## 61 Introduction

62 Antenatal care (ANC) is essential for the health of pregnant women and their fetuses, aiding in
63 labor preparation and the recognition of pregnancy-related warning signs (1). ANC interruption
64 is defined as not receiving ANC care as o f	the new World Health Organization (WHO)
65 recommendations, pregnant mothers should attend at least eight antenatal care (ANC) visits
66 throughout their pregnancy.
67 ~~at least 8 visits, according to new guidelines~~ (2). In conflict-affected populations, women and
68 children face disproportionate morbidity (3).
69 From 2000 to 2017, global maternal mortality ratios (MMR) decreased by 38%, from 342 to 211
70 deaths per 100,000 live births (5). However, African nations in conflict or post-conflict situations
71 still report high maternal mortality rates. For example, in Tigray, the MMR rose ~~dramatically~~
72 drastically from 186 per 100,000 live births in 2019 to 840 in 2023 due to the war (6, 7, 8, 9, 10).
73 While the global average for ANC coverage exceeds 75%, many conflict-affected regions fall
74 below this standard. In Somalia, for instance, ANC coverage is only 9.4%, and Afghanistan
75 reports a skilled birth attendant (SBA) coverage of 17.8% (11, 12, 13, 14). In Tigray, maternal
76 ANC service ~~utilization~~ utilisation plummeted from 90% before the conflict to 50% during the
77 war (15, 16).
78 Understanding the spatial patterns of ANC interruption—identifying specific areas within Tigray
79 with varying interruption rates—and the predictors of these interruptions, such as conflict
80 impact, ~~healthcare access, and socioeconomic factors, remains~~ and socioeconomic factors
81 remain limited. Therefore, assessing the ~~magnitude~~ Percentage of women with ANC
82 interruptions and their peculiar factors ~~barriers~~ is crucial for developing effective maternal health
83 interventions in Tigray post-conflict northern Ethiopia..

## 84 ~~Materials and m~~Methods and Materails

85 **Study Setting, design, data source**
86 This cross-sectional study ~~utilized survey data from~~ is one of ~~integrated~~the integrated surveys
87 aimed at evaluating cost-effectiveness in post-conflict settings on identifying the behavioral and
88 social drivers of COVID-19 vaccination, maternal and child health services utilisation in Tigray,
89 Ethiopia. ~~Conducted by Mekelle University, the Tigray Health Research Institute, and the Tigray~~
90 ~~Regional Health Bureau~~, the study ~~research~~ involved 13,915 female participants, of whom
91 10,654 were of reproductive age (15-49 years), and 3,933 had been pregnant since the onset of
92 the conflict. The analysis focused on 2,444 women who were pregnant women during the
93 conflict ~~and~~ had received antenatal care (ANC). Data collection occurred over a month, from
94 August ~~July~~ 1 to ~~July~~ 30, 2023[17]. ~~This systematic approach allowed for a comprehensive~~
95 ~~examination of factors affecting interruption in ANC within this vulnerable population~~
96 **Study Design:** Community based cross sectional study
97 **Measurements and variables**
98 **Dependent variable; antenatal care interruption:**
99 **Antenatal care interruption** was measured as a binary variable indicating whether ANC
100 services were interrupted (1) or not interrupted (0) during the conflict period. This was assessed
101 through participant’s interviews in selected households of mothers who were following ANC
102 visit during the conflict and interrupted their ANC visit for different reasons.
103 **Independent Variables**
104 Independent variables include a range of socio-economic, geographical, and conflict-related
105 factors:
106 **Socio-economic factors:** age of mother, education level, occupation, marital status, number of
107 children, media access, ownership of radio, mobile and television

108

109 **Geographical Factors: zone of residence, distance to health facility:** Distance to nearest
110 health facility was measured in kilometers and time taken to double trip from the home to the
111 nearest health facility providing ANC services. Accessibility index a composite measure
112 reflecting road conditions, transportation availability, and geographic barriers, security. **Conflict-**
113 **related Factors**; displacement status whether the mother or household has been displaced due to
114 conflict and it was labeled as (yes/no).Perceived security **a** qualitative measure based on survey
115 responses regarding feelings of safety when accessing health services. Loss, damage of property
116 and nearby persons who care for them.

**Formatted:** Font: Not Bold

117 **Data collection methods:** Structured questionnaires interviewer administered was used to

**Formatted:** Level 3, Space Before: Auto, After: Auto, Line spacing: 1.5 lines

118 collect data from expectant mothers to gather data on ANC usage, socio-economic status, and
119 perceived barriers. Geographic positioning Systems (GPS) data were collected through ODK tool
120 using mobile tablets and X-Y coordinate of the household were recorded

**Formatted:** Font: Bold

121 **Statistical Method and Analysis**

**Formatted:** Line spacing: 1.5 lines

122 Univariate descriptive, bivariate ,chi-square and multivariate spatial logistic regression
123 approaches were employed to analyze the ANC data from targeted zones of the Tigray Region.
124 The univariate analysis were ~~made~~ used ~~of~~ to summarize & describe the demographic variables
125 (e.g., age, gender, etc), ~~by means of~~ using frequencies, percentages, means, and standard
126 deviations provided a detailed overview. Associations between categorical variables (e.g.,
127 gender, zone, etc) and ANC ~~interruption were~~ interruptions ~~assessed~~were assessed using
128 ~~bivariate~~ Chi-square or Fisher’s exact tests. To model the relationship between risk factors and
129 ~~cholera~~ occurrence across zones, we used ANC interruption. Spatial logistic regression is an
130 advanced modeling approach that incorporates zones, representing administrative or geographic
131 divisions, to analyze binary outcomes while accounting for spatial dependencies. The model
132 extends ordinary logistic regression by including spatial random effects (∅_𝐳_) to capture
133 unobserved spatial variation between zones, improving the accuracy of estimates and inference.
134 The model is expressed as:

135

𝑷_𝒊_

𝐥𝐨𝐠𝐢𝐭(𝑷 |)) = 𝐥𝐧 {	} = 𝛃

𝒑

+ ∑ 𝛃 𝑿

+ ∅ ,	𝐳 = 𝐒, 𝐒𝐄, 𝐌, 𝐄, 𝐂, 𝐍𝐖

^𝒊^	𝟏 − 𝑷𝒊)

𝐨

𝒌=𝟏

𝐤 𝒊𝒌	𝐳

136 where 𝑷𝒊 represents the probability of the outcome for observation 𝒊, 𝛃𝐨 is the intercept, 𝑿𝒊𝒌 are
137 predictors, 𝛃𝐤 are their coefficients, and ∅𝐳 captures the spatial effect for zone 𝐳 . This approach
138 allows the model to account for spatial clustering and patterns, addressing biases and
139 inaccuracies that arise from spatial autocorrelation in ordinary logistic regression. Spatial
140 autocorrelation in the dependent ~~variable was~~ variables were assessed using Moran’s I test. The
141 spatial logistic regression model incorporated structured spatial random effects, is modeled
142 through a Conditional Autoregressive (CAR) framework, to account for spatial dependencies
143 between zones. This makes it ~~a valuable~~ valuable tools ~~tool~~ for policymakers and researchers
144 aiming to address spatial inequalities in various fields, including public health, urban
145 development, and environmental management
146 Spatial logistic regression provides several advantages in analyzing binary outcomes, particularly
147 by incorporating spatial dependencies that traditional models often overlook. This approach
148 enhances model ~~fit~~ fitness and predictive accuracy, facilitating the identification of high-risk
149 areas through spatial clustering of central zone ,north western zone, southern,, south eastern
150 ,easten and Mekelle zones of the Tigray region Ethiopia. It also uncovers spatial patterns and
151 clusters, which can inform targeted public health interventions [18,19,20]. The analysis was
152 conducted using R version 4.4.1, with ~~weighting~~ Weighing applied to adjust for non-proportional
153 sampling. Both exploratory and confirmatory analyses were carried out, including descriptive
154 statistics, ~~visualizations~~visualisations, and inferential statistics. Predictors with p < 0.05 in the
155 bivariate and multivariate analysis were incorporated into the final spatial logistic regression
156 model.

157	**Results**

158 **Sociodemographic characteristics of participants**
159 The study participants are divided into two groups: ~~hosts~~ hosts communities 2,069 (84.7%, 95%
160 CI: [83.5%, 85.9%]) and internally displaced people 375 (15.3%, 95% CI: [14.1%, 16.5%]). The
161 mean age of participants is 28 years, with a standard deviation of 0.05.
162 A significant portion of the participants have ~~has~~ not completed formal education. Specifically,
163 835 individuals (34.2%, 95% CI: [32.5%, 36.0%]) are illiterate, while 754 (30.9%, 95% CI:
164 [29.2%, 32.6%]) completed elementary school. Additionally, 540 participants (22.1%, 95% CI:
165 [20.7%, 23.5%]) completed secondary school, and only 201 individuals (8.2%, 95% CI: [7.3%,
166 9.1%]) hold a college degree or higher.
167 Geographically, most participants reside in rural areas, with 1,530 (62.6%, 95% CI: [60.9%,
168 64.3%]) living in such regions. In contrast, 190 participants (7.8%, 95% CI: [6.5%, 9.1%]) live
169 in semi-urban areas, while 724 (29.6%, 95% CI: [27.9%, 31.3%]) inhabit urban locations. A
170 large majority, 2,239 participants (91.6%, 95% CI: [90.5%, 92.7%]), are married.
171 Access to resources is limited among the participants. For instance, 1,615 individuals (66.1%,
172 95% CI: [64.4%, 67.8%]) lack electricity, and 1,373 (56.2%, 95% CI: [54.5%, 57.9%]) do not
173 have access to public transportation. Media ownership is also low, with only 308 participants
174 (12.6%, 95% CI: [11.3%, 13.9%]) owning a radio. In contrast, mobile phone ownership is more
175 common, with 1,138 individuals (46.6%, 95% CI: [44.9%, 48.3%]) possessing a mobile phone.
176 Television ownership is relatively low, as just 400 participants (16.4%, 95% CI: [15.1%, 17.7%])
177 have a television or detail see “**Table 1**.”

178

179	Table 1: Socio-demographic of pregnant women following ANC during the war **(N 2444)**

**Variables	Response	Frequency(n)	Percent (%)**

*South*	346	14.2

*South-east*	233	9.5

*Mekelle*	308	12.6

Zone

*Eastern*	520	21.3

*Central*	624	25.5

*Northwest*	413	16.9

Participant

|  | | *IDP** | 375 | 15.3 |  |
| --- | --- | --- | --- | --- | --- |
|  | | *Orthodox* | 2369 | 96.9 |  |
| Religion |  | *Muslim* | 75 | 3.1 |  |
|  |  | *Rural* | 1530 | 62.6 |  |
| Residence |  | *Semi-urban* | 190 | 7.8 |  |
|  |  | *Urban* | 724 | 29.6 |  |
|  |  | *Unable to read and write* | 835 | 34.2 |  |
|  |  | *Able to read and write* | 114 | 4.7 |  |
| ~~Highest~~ E~~e~~ducational | Status ~~level~~ | *Elementary school* | 754 | 30.9 |  |
| ~~attained~~ |  | *Secondary school* | 540 | 22.1 |  |
|  |  | *College and above* | 201 | 8.2 |  |
|  |  | *Single* | 41 | 1.7 |  |
|  |  | *Married* | 2239 | 91.6 |  |
| Marital status |  | *Divorced* | 127 | 5.2 |  |
|  |  | *Widowed* | 11 | 0.5 |  |
|  |  | *Separated* | 26 | 1.1 |  |
| Access to electricity |  | *Yes* | 829 | 33.9 |  |
|  | | *No* | 1615 | 66.1 | |
| Radio ownership | | *Yes* | 308 | 12.6 | |
|  | | *No* | 2136 | 87.4 | |
| Television ownership | | *Yes* | 400 | 16.4 | |
|  | | *No* | 2044 | 83.6 | |
|  | | *Yes* | 1138 | 46.6 | |
| Mobile phone ownership | | *No* | 1306 | 53.4 | |
| Have been displaced | | *Yes* | 1386 | 56.7 | |
|  | | *No* | 1058 | 43.3 | |
|  | | *Yes* | 1421 | 58.1 | |
| Does the war impact your life | | *No* | 1023 | 41.9 | |
| Time taken double trip to health post | | *< 1 hour* | 1583 | 64.8 | |

*Host*	2069	84.7

*>=1 hour*	861	35.2

180

181 **Sexual and reproductive health characteristics of participants**

**Formatted:** Section start: Continuous

182 The study participants reported varying experiences regarding pregnancies and births. The
183 majority had 2 pregnancies (21.7%), followed by 1 pregnancy (20.3%). The number of
184 pregnancies decreases with higher counts: 3 pregnancies (17.2%), 5 (10.4%), 6 (6.5%), 7 (4.7%),
185 and 8 (5.2%).
186 In terms of births, most participants reported 1 birth (22.7%) or 2 births (22.6%), with
187 subsequent numbers decreasing: 3 births (16.6%), 4 (11.9%), 5 (9.0%), 6 (5.7%), 7 (3.8%), and 8
188 (3.7%). Additionally, 4.1% have not given birth.
189 Regarding live births, the distribution is as follows: 1 live birth (23.2%), 2 (22.2%), 3 (16.6%), 4
190 (11.9%), 5 (8.5%), 6 (5.6%), 7 (3.5%), and 8 or more (3.2%). Notably, 5.3% of participants
191 reported having no live births.
192 The majority, 2,328 (95.0%), reported 0 stillbirths, while 95 (3.9%) had 1 stillbirth, 18 (0.7%)
193 had 2, 2 (0.08%) had 3, and 1 (0.04%) had 4 stillbirths.
194 Regarding abortions, 1,622 (66.4%) reported having 0 abortions in their lifetime. However, a
195 notable portion has experienced abortions: 698 (28.6%) reported 1 abortion, 100 (4.1%) reported
196 2, 18 (0.7%) reported 3, 5 (0.2%) reported 4, and 1 (0.04%) reported 5 **[Figure 3]**.
197 **Fig 3. The line graph show the reproductive history of study participants of interruption of**

**Formatted:** Space After: 0 pt, Line spacing: 1.5 lines

198 **ANC during the war in Tigray 2023.**

**Formatted:** Font: Bold

199

Pregnancy intentions among participants

**Commented [MA1]:** Please check the table of this RESULT descrption

**Formatted:** Line spacing: 1.5 lines

**Formatted:** Line spacing: 1.5 lines

200 The study revealed varied pregnancy intentions among participants. A majority, 1,455 (59.5%),
201 reported actively trying to become pregnant at the time of conception. However, a significant
202 portion, 738 (30.2%), expressed a desire to wait until later to have children. Additionally, 251
203 (10.3%) stated that they did not wish to have any more children when they became pregnant.
204 findings of this study indicate a diverse range of pregnancy intentions within the study
205 population, highlighting that while many participants were intentionally seeking pregnancy, a
206 notable number preferred to delay or avoid further pregnancies. For more details, see “Table 2”.
207 Antenatal care (ANC) interruption
208 The prevalence of antenatal care (ANC) interruption was found to be 24.8% (95% CI: [23.1%,
209 26.5%]), indicating that nearly one in four women encountered difficulties in maintaining
210 consistent ANC. The primary reasons for these interruptions included insecurity (45.3%, 95%
211 CI: [43.1%, 47.5%]), health facility closures (39.8%, 95% CI: [37.6%, 42.0%]), and a lack of
212 supplies and medicine for ANC (33.1%, 95% CI: [31.0%, 35.2%]) for additional details **(Fig 1.)**
213 ANC interruption by zone
214 The spatial distribution of ANC interruptions across the Tigray region was as follows:
215 Northwestern zone 22.8%, Central zone 29.1%, Eastern zone 17.7%, Southern zone 16.7%,
216 Southeastern zone 6.4%, and Mekelle zone 7.3%. However, the Western zone of Tigray was not
217 accessible for our study due to security concerns [ Figure 2].
218 **Fig 1. Special distribution of ANC interruption ~~acroos~~ across the zone of Tigray Region**
219 **(n=2444)**
220 **(Fig2.) Reasons ~~of~~ for interruption of ANC care during the war from ~~to~~ Pretoria**
221 **November 2020 to August 2022 agreement of the Tigray region, Northern Ethiopia**
222 **Bivariate Analysis: Impact of War on Antenatal Care (ANC) Visits**
223 The study found that 75.2% (1,839, 95% CI: [73.9%, 76.5%]) of participants did not postpone or
224 interrupted their antenatal care (ANC) visits due to the ongoing war, while 24.8% (605, 95% CI:
225 [23.5%, 26.1%]) reported interruptions. Among those with a sick family member in the last two
226 weeks, 14.3% (349, 95% CI: [12.9%, 15.7%]) did not postpone their ANC visit, and 7.0% (171,
227 95% CI: [5.9%, 8.1%]) did.
228 The war's impact was significant, with 96.5% (2,358, 95% CI: [95.4%, 97.6%]) reporting a
229 family member were disabled; of these, 72.7% (1,778, 95% CI: [71.0%, 74.4%]) did not
230 postpone their ANC visit, while 23.7% (580, 95% CI: [22.2%, 25.2%]) did. Additionally, 97.0%
231 (2,370, 95% CI: [96.0%, 98.0%]) experienced the loss of a family member, with 73.2% (1,790,
232 95% CI: [71.5%, 74.9%]) continuing their ANC visits. A small fraction, 1.4% (34, 95% CI:
233 [0.8%, 2.0%]), reported a woman in their household experiencing rape due to the war, with only
234 1.1% (27, 95% CI: [0.5%, 1.7%]) not postponing ANC visits.
235 Property damage was reported by 53.3% (757, 95% CI: [51.0%, 55.6%]) of participants, with
236 38.2% (543, 95% CI: [35.9%, 40.5%]) not postponing their ANC visits. Looting affected 89.7%
237 (1,274, 95% CI: [88.5%, 90.9%]), with 66.1% (939, 95% CI: [64.5%, 67.7%]) continuing their
238 visits. Destruction of property was noted by 27.3% (388, 95% CI: [25.6%, 29.0%]) of
239 participants, where 20.3% (288, 95% CI: [18.7%, 21.9%]) did not postpone. Displacement
240 impacted 56.7% (1,386, 95% CI: [54.9%, 58.5%]), with 42.0% (1,027, 95% CI: [40.3%, 43.7%])
241 not postponing their ANC visits.
242 In terms of distance to health posts, 64.8% (1,583, 95% CI: [63.0%, 66.6%]) reported a round
243 trip of less than 1 hour, while 35.2% (861, 95% CI: [33.4%, 37.0%]) reported longer trips. Those
244 who did not postpone their ANC visits were more likely to live closer to health facilities (49.7%
245 vs. 15.1%).
246 Functionality of nearby health facilities was high, with 75.8% (1,303, 95% CI: [74.1%, 77.5%])
247 reporting nearby health posts as functional and 97.0% (2,371, 95% CI: [96.0%, 98.0%]) for
248 health centers/hospitals. Access to public transport was reported by 43.8% (1,071, 95% CI:
249 [41.9%, 45.7%]), with those who had not postponed ANC visits slightly more likely to have
250 access (32.8% vs. 11.0%).
251 Regarding electricity supply, 33.9% (829, 95% CI: [32.1%, 35.7%]) reported having it, and those
252 who did not postpone their ANC visits were somewhat more likely to have electricity (26.5% vs.
253 9.0%). Media ownership was limited, with 12.6% (308, 95% CI: [11.3%, 13.9%]) owning a radio
254 and 16.4% (400, 95% CI: [15.1%, 17.7%]) owning a television. Those who did not postpone
255 their ANC visits were slightly more likely to own a radio (9.6% vs. 3.5%) or a TV (12.6% vs.
256 4.5%). Mobile phone ownership was similar between both groups, with 46.6% (1,138, 95% CI:
257 [44.9%, 48.3%]) owning a phone

258

259

260

261 **Table 2: War and ANC interruption of ANC care during the war to Pretoria ~~Nov2020~~** November 2020 **to August 2022**
262 **agreement of the Tigray region Northern Ethiopia(N=2444)**

**Predictors**

**Categorie s**

**ANC Interruption due to**

**war	χ2**

**P-**

**value**

**Predictors**

**Categorie s**

**ANC Interruption due to**

**war	χ2**

**P-**

**value**

**No(*n=1839*) Yes(*n=605*)	No(*n=1839*)	Yes(*n=605*)**

1214(49.7%

**Sick Last 2 Weeks**

Yes	349 (14.3%) 171 (7%)

1490

**23.4**

**4**

**<0.00**

**1**

**HP Distance**

<= 1 hour

)	369(15.1%)

**5.03	0.025**

No	434 (17.8%)

|  | (61.0%) |  | >= 1 hour | 625(25.6%) | 236(9.7%) |
| --- | --- | --- | --- | --- | --- |
| Yes | 61(2.5%) | 25(1.0%) | Yes | 994(57.8%) | 309(18.0%) |
| **0.89 0.345 Functional HP** | | | | | |
| No 177(72.7%) | | 580(23.7%) | No | 249(14.5%) | 93(5.4%) |
| Yes 49(2.0%) | | 25(1.0%) | Yes | 56(37.3%) | 19(12.7%) |

**Disabled War	1.78	0.183**

|  | | | **38.5** | **<0.00** |
| --- | --- | --- | --- | --- |
| No | 18 (12.0%) | 57 (38.0%) | **1** | **1** |
| Yes | 19 (3.2%) | 90 (15.3%) | **9.03** | **0.003** |

**Lost Family member in**

**War**	No

1790(73.2%

)	580(23.7%)

**Functional HC**

**Rape victim HHs**

| No | 153 (26.0%) | 326 (55.4%) |  |
| --- | --- | --- | --- |
| Yes | 24 (23.5%) | 78 (76.5%) | 0.94 |
| No | 18 (24.0%) | 57 (76.0%) | 2 |
| Yes | 24 (23.5%) | 78 (76.5%) | 0.94 |
| No | 18 (24.0%) | 57 (76.0%) | 2 |
| Yes | 24 (23.5%) | 78 (76.5%) | 0.94 |

Yes	27(1.1%)	7(0.3%)

1812(74.1%

No

|  | | ) | 598(24.5%) |
| --- | --- | --- | --- |
| **War Damage** | Yes | 490(34.5%) | 174(12.2%) |
|  | No | 543(38.2%) | 214(15.1%) |

**Access to transport to HP/HC**

**Electricity Supply**	0.005

**War Looted**

| **3.34** | **0.068** |
| --- | --- |
| **0.32** | **0.571** |
| 0.76 | 0.383 |

Yes	94(6.6%)	53(3.7%)	11.3

No

| 939(66.1%) | 335(23.6%) | 8 |  | |
| --- | --- | --- | --- | --- |
| 1027(42.0% |  |  |  |  |
| ) | 359(14.7%) | 2.26 | 0.138 | **TV in HH** |
| 812(33.2%) | 246(10.1%) |  |  |  |

11.38	**Radio in the HH**

0.005

**Displaced due to War**

Yes No

0.005

2

| No | 18 (24.0%) | 57 (76.0%) |  |
| --- | --- | --- | --- |
| Yes | 24 (23.5%) | 78 (76.5%) | 0.94 |
|  |  |  | 2 |
| No | 18 (24.0%) | 57 (76.0%) | |

263

**Formatted:** Right: 1.31", Section start: Continuous

| 1033(42.3% |  |
| --- | --- |
| ) | 388(15.9%) |
| 806(33.0%) | 217(8.9%) |

**War Impact in HH**

Yes No

11.8

5

**<0.00**

**1**

**Mobile Ownership**

0.005

**Formatted:** Line spacing: 1.5 lines

264

**Formatted:** Section start: Continuous

265 **~~Sexual and reproductive health characteristics of participants~~**
266 ~~The study participants reported varying experiences regarding pregnancies and births. The~~
267 ~~majority had 2 pregnancies (21.7%), followed by 1 pregnancy (20.3%). The number of~~
268 ~~pregnancies decreases with higher counts: 3 pregnancies (17.2%), 5 (10.4%), 6 (6.5%), 7 (4.7%),~~
269 ~~and 8 (5.2%).~~
270 ~~In terms of births, most participants reported 1 birth (22.7%) or 2 births (22.6%), with~~
271 ~~subsequent numbers decreasing: 3 births (16.6%), 4 (11.9%), 5 (9.0%), 6 (5.7%), 7 (3.8%), and 8~~
272 ~~(3.7%). Additionally, 4.1% have not given birth.~~
273 ~~Regarding live births, the distribution is as follows: 1 live birth (23.2%), 2 (22.2%), 3 (16.6%), 4~~
274 ~~(11.9%), 5 (8.5%), 6 (5.6%), 7 (3.5%), and 8 or more (3.2%). Notably, 5.3% of participants~~
275 ~~reported having no live births.~~
276 ~~The majority, 2,328 (95.0%), reported 0 stillbirths, while 95 (3.9%) had 1 stillbirth, 18 (0.7%)~~
277 ~~had 2, 2 (0.08%) had 3, and 1 (0.04%) had 4 stillbirths.~~
278 ~~Regarding abortions, 1,622 (66.4%) reported having 0 abortions in their lifetime. However, a~~
279 ~~notable portion has experienced abortions: 698 (28.6%) reported 1 abortion, 100 (4.1%) reported~~
280 ~~2, 18 (0.7%) reported 3, 5 (0.2%) reported 4, and 1 (0.04%) reported 5~~ **~~(Fig 3)~~**~~.~~
281 **~~Fig 3. line graph showing reproductive history of study participants of interruption of ANC~~**
282 **~~during the war of Tigray 2023.~~**
283 Pregnancy intentions among participants
284 The study revealed varied pregnancy intentions among participants. A majority, 1,455 (59.5%),
285 reported actively trying to become pregnant at the time of conception. However, a significant
286 portion, 738 (30.2%), expressed a desire to wait until later to have children. Additionally, 251
287 (10.3%) stated that they did not wish to have any more children when they became pregnant.
288 ~~These~~ findings of this study indicate a diverse range of pregnancy intentions within the study
289 population, highlighting that while many participants were intentionally seeking pregnancy, a
290 notable number preferred to delay or avoid further pregnancies. For more details, see “Table 2”.

291

**Formatted:** Font: Not Bold

**Formatted:** Numbering: Continuous, Number of columns: 1, Force equal column width

**Formatted:** Indent: Left: 0.6", Line spacing: 1.5 lines

**Formatted:** Font: (Default) Times New Roman, 12 pt

**Formatted:** List Paragraph, Indent: Left: 0.6", Line spacing:

1.5 lines, Tab stops: 0.6", Left

**Formatted:** Line spacing: 1.5 lines

**Formatted:** Font: (Default) Times New Roman

**Formatted:** Font: (Default) Times New Roman, Not Expanded by / Condensed by

292 Multivariate Analysis: Parameter Estimates from SLR
293 The Mora~~n’s~~ I test indicated significant spatial autocorrelation in ANC interruption (Moran’s I =
294 0.546, p-value < 0.001), indicating the presence of spatial autocorrelation in the dependent
295 variable, validating the need for spatial logistic regression. Incorporating spatial dependencies is
296 crucial, as neglecting them in ordinary logistic regression may result in biased parameter
297 estimates and inaccurate inferences.
298 Model fitness improved substantially with the inclusion of the spatial term, as reflected by an
299 increase in log-likelihood from -1200 to -1050 and a decrease in AIC from 2410 to 2110. The
300 pseudo R-squared increased from 0.55 to 0.72, demonstrating better explanatory power.
301 Multicollinearity diagnostics showed all Variance Inflation Factors (VIFs) below 3, indicating no
302 serious multicollinearity issues among predictors.
303 The spatial logistic regression model identified several key factors influencing the interruption of antenatal
304 care (ANC) during the war. For every unit increase in distance from the health center, the log-odds of
305 interrupting ANC increased by 0.00~~0353~~1 (SE = 0.00~~0103~~1, p < 0.001). Geographic differences also
306 played a significant role; women living in the south-east zone had a 0.11~~876~~2 lower log-odds of
307 interrupting ANC compared to those in the north-west zone (SE = 0.05~~0501~~, p = 0.0~~18685~~2).
308 Additionally, women in the eastern zone exhibited a 0.16~~452~~ lower log-odds of interrupting ANC
309 compared to the north-west zone (SE = 0.04~~8012~~, p = 0.00~~0611~~1).
310 The number of children under five in the household was another important factor, with each additional
311 child increasing the log-odds of interrupting ANC by 0.08~~3134~~ (SE = 0.0~~17295~~2, p < 0.001). Furthermore,
312 households without a radio had a 0.08939 lower log-odds of interrupting ANC compared to those with a
313 radio (SE = 0.03~~3425~~, p = 0.00~~7489~~1). These results suggest that longer distances to health centers,
314 residing in the north-west zone, having more young children, and not owning a radio were all associated
315 with a greater likelihood of ANC interruption in the war-affected Tigray region of Ethiopia. In contrast,
316 the analysis for Mekelle indicated an estimate of -0.04, a standard error of 0.07, and a z-value of -0.55,
317 resulting in a p-value greater than 0.05, which shows no significant effect on ANC interruption compared
318 to the north-west zone.
319 In contrast, the estimate for women in Mekelle was -0.038 (SE = 0.069, p > 0.05), indicating no
320 statistically significant difference in ANC interruption compared to the north-west zone. These findings
321 highlight the importance of geographic location, access to information, and household burden in shaping
322 ANC service continuity in war-affected areas of Tigray, Ethiopia. Conversely, the eastern zone showed
323 significantly lower odds of ANC interruption, with an estimate of -0.160 (SE = 0.050, p < 0.05), indicating

**Formatted:** Line spacing: 1.5 lines

**Formatted:** Line spacing: 1.5 lines

**Formatted:** Line spacing: 1.5 lines

**Formatted:** Line spacing: 1.5 lines

324 better adherence to ANC in this zone as seen in Table 3.
325 ~~Conversely, the eastern zone had an estimate of -0.16, a standard error of 0.05, and a z-value of -3.43, with~~
326 ~~a p-value less than 0.05, indicating significantly lower odds of ANC interruption and better adherence in~~
327 ~~this zone for detail see “Table 3”~~
328 Table 3: Spatial logistic regression model analysis, shows the parameter estimates, standard errors, z-
329 values, and p-values for the predictors of interruption of ANC during the war in Tigray 2023.

Predictors

(Intercept) South-east zone Mekelle zone Eastern zone Central zone Northwest zone Age

Number under five children Number of Pregnancies Family size

Perceived wealth medium Perceived wealth poor Radio ownership Television ownership Mobile phone ownership

Perceived food Security moderately

Estimate 0.164124

-0.11876

-0.0377

-0.16452

0.038805

0.035293

0.000458

0.083134

0.002781

0.002114

0.08881

0.060797

-0.08939

-0.02766

-0.04237

Std. Error	Z-value

0.152572	1.0757

0.050501	-2.3518

0.068806	-0.5479

0.048012	-3.4266

0.041975	0.9245

0.046268	0.7628

0.002425	0.189

0.017295	4.8068

0.008414	0.3305

0.007472	0.2829

0.128912	0.6889

0.130133	0.4672

0.033425	-2.6743

0.051554	-0.5365

0.023887	-1.7739

Pr(>|z|) 0.28~~2055~~

0.01~~8685~~

0.58~~3761~~

0.00~~0611~~1***

0.35~~524~~

0.44~~5589~~

0.85~~008~~

1.53~~E-06~~

0.74~~1027~~

0.77~~725~~

0.49~~0878~~

0.64~~0364~~

0.007~~489~~**

0.59~~1633~~

0.07~~6077~~*

**Formatted:** Line spacing: 1.5 lines

**Formatted:** Line spacing: 1.5 lines

**Formatted:** Line spacing: 1.5 lines

**Formatted:** Line spacing: 1.5 lines

**Formatted:** Line spacing: 1.5 lines

secured

0.03115936

0.035233

-0.04285

-0.00044

0.000353

-0.00869

0.047596	0.6547

0.051334	0.6863

0.022561	-1.8993

0.000266	-1.6706

0.000103	3.415

0.029711	-0.2925

0.51~~2689~~

0.49~~2493~~

0.05~~752~~*

0.09~~4804~~

0.00~~0638~~1***

0.76~~9874~~

Perceived food Security insecure War impacted me

Distance health post in minutes Distance of health center in minutes Access to transport health Center

330	**p<0.05,	**p<0.01,	***p<0.001*

**Formatted:** Line spacing: 1.5 lines

**Formatted:** Line spacing: 1.5 lines

**Formatted:** Line spacing: 1.5 lines

**Formatted:** Line spacing: 1.5 lines

**Formatted:** Line spacing: 1.5 lines

**Formatted:** Line spacing: 1.5 lines

**Formatted:** Line spacing: 1.5 lines

**Formatted:** Line spacing: 1.5 lines

**Formatted:** Line spacing: 1.5 lines

**Formatted:** Line spacing: 1.5 lines

**Formatted:** Line spacing: 1.5 lines

**Formatted:** Line spacing: 1.5 lines

**Formatted:** Line spacing: 1.5 lines

331	**Discussion**

332 The prevalence of antenatal care (ANC) interruption was found to be 605 (24.8%), indicating that nearly
333 one in four women faced challenges in maintaining consistent ANC. ~~This is a critical concern since ANC~~
334 ~~is a well-established and cost-effective intervention that provides essential care to pregnant women from~~
335 ~~early pregnancy to delivery [21]~~. The interruption was notably more severe among mothers residing in
336 rural areas (370, 61.2%) compared to semi-urban (51, 8.4%) and urban (184, 30.4%) settings. A
337 significant majority of those affected were host residents (519, 85.8%) rather than internally displaced
338 mothers (86, 14.2%). Additionally, a large portion of participants were married (563, 91.1%).
339 Similar studies conducted in Syria in 2014 to 2017 have found that violent events significantly reduce
340 the likelihood of pregnant women seeking and accessing essential healthcare services ~~related to maternal~~
341 ~~health~~ showed negative correlation between bombardments and consultations and ANC visits was
342 reported the RR was 0.95 (95% CI 0.94–0.97) and 0.95 (95% CI 0.93–0.98), respectively.
343 . The fear and risks associated with ongoing conflicts deter pregnant women from seeking necessary care,

**Formatted:** Left, Space Before: 1.45 pt, After: 0 pt, Line spacing: 1.5 lines, No widow/orphan control, Don't adjust space between Latin and Asian text, Don't adjust space between Asian text and numbers

344 increasing the vulnerability of both mothers and their unborn children to potential health complications
345 [22]. Research in Yemen highlighted those pregnant women in remote, rural areas encountered substantial
346 barriers to reaching ANC clinics due to infrastructure damage, lack of public transportation, and financial
347 constraints [23]. Furthermore, studies in Afghanistan have shown that the Taliban's restrictions on
348 women's movement and access to healthcare during conflicts severely limited pregnant women's ability to
349 attend ANC appointments [24,25]. These challenges were particularly pronounced in rural areas compared
350 to urban settings [26]. This is a critical concern since ANC is a well-established and cost-effective
351 intervention that provides essential care to pregnant women from early pregnancy to delivery [21]
352 ~~This situation contrasts with the internal resolution of the World Health Organization (WHO 2012), which~~

**Formatted:** Left, Space After: 0 pt, Line spacing: 1.5 lines

353 ~~emphasizes the need for leadership in documenting evidence of attacks against health workers, facilities,~~
354 ~~and patients in areas affected by armed conflict~~ The high prevalence of interruptions in antenatal care
355 (ANC) services during conflict situations contradicts the internal resolution of the World Health
356 Organization (WHO, 2012), which stresses the importance of leadership in documenting attacks on health
357 workers, facilities, and patients. [27].
358 The results of the spatial logistic regression analysis in the war-affected Tigray region of Ethiopia revealed

**Formatted:** Line spacing: 1.5 lines

359 several key factors influencing ANC interruptions. For instance, the distance to health centers was a
360 significant factor; for every unit increase in distance (in minutes), the log-odds of discontinuing ANC
361 increased by 0.000353 (SE = 0.000103, p < 0.001). This finding aligns with studies conducted in Syria,
362 where ongoing conflict and insecurity significantly disrupted access to ANC services. this was compared

**Formatted:** Left, Space After: 0 pt, Line spacing: 1.5 lines

**Formatted:** Font: (Default) Segoe UI, Font color: Custom Color(RGB(80,80,80))

**Formatted:** Line spacing: 1.5 lines

363 by the time taken to reach health facilitie both before the war and during the war even if the distance is
364 constant the time taken in these two periods were different this may be the security reasons, blockage,
365 checkpoints here and there. Women reported facing barriers such as transportation difficulties, fear of
366 checkpoints, and financial constraints when trying to reach health facilities [28,29]. Additionally, a study
367 in Syria found that women living in besieged or hard-to-reach areas experienced much greater difficulty
368 accessing ANC services due to transportation challenges, security concerns, and a lack of functioning
369 health facilities [30].
370 Living in the north-west zone of the war-affected Tigray region was associated with a higher likelihood of
371 ~~discontinuing antenatal~~interruption antenatal care (ANC). this may be the blockage, movement from place
372 to place, severity of war may be higher in this area and also because it is far from Mekelle the capital city
373 of the Tigray region which is about 300kms some of the humanitarian organizations were not reaching this
374 area.this is also similar to studies conducted in Syria where bombardment and distant villages from the
375 centers interrrupted ANC service than those who are near the centers (22). This is consistent with In conflict
376 settings, healthcare access is severely compromised due to the destruction of facilities and evacuation of medical
377 personnel, leading to increased travel times and reduced service availability (31)
378 Conversely, while distance is a critical factor, other elements such as socio-economic status,
379 education, and community support also play significant roles in healthcare access and continuity
380 in conflict-affected regions (32)
381 Also align with Previous studies have consistently highlighted that geographic disparities, often
382 exacerbated by conflict, play a critical role in shaping healthcare utilization. For instance, in
383 conflict-affected regions of Afghanistan, women in remote or insecure areas faced higher
384 barriers to ANC attendance due to disrupted infrastructure and security concerns, a pattern that
385 resonates with the higher likelihood of ANC discontinuation(33)Additionally, households with a
386 greater number of young children were more prone to discontinuation, as were those that did not own a
387 radio. These findings underscore the unique challenges faced by pregnant women in war-affected regions,
388 highlighting the need for improved access to health facilities, addressing transportation and security
389 concerns, and implementing targeted interventions to support households with multiple young children. ~~By~~
390 ~~addressing these factors, it may be possible to enhance the continuity of antenatal care and ultimately~~
391 ~~improve maternal and child health outcomes in the Tigray region.~~
392 Research from Yemen and the Democratic Republic of the Congo further illustrates the severe impact
393 Impact of armed conflicts and civil wars on maternal healthcare access in sub-Saharan Africa. In Yemen,
394 the escalation of the civil war led to the closure of many health facilities, significantly hindering pregnant
395 women’s ability to receive regular ANC [3~~1~~4,3~~2~~5]. Similarly, findings from the Democratic Republic of
396 the Congo indicate that armed conflicts and displacement forced many pregnant women to miss critical
397 ANC visits, resulting in poorer maternal and child health outcomes [3~~3~~6,3~~4~~7,3~~5~~8]. Pregnant women
398 displaced to rural areas in the DRC exhibited much lower ANC attendance rates compared to those who
399 remained in or fled to urban centers [36]. Overall, the effects of civil wars on sub-Saharan Africa have
400 severely compromised maternal and child health services [39~~7~~].
401 The spatial logistic regression analysis highlighted distance to health centers as a significant factor; for

**Formatted:** Space After: 0 pt, Line spacing: 1.5 lines

402 every unit increase in distance (in minutes), the log-odds of discontinuing ANC increased by 0.00~~0353~~1
403 (SE = 0.00~~0103~~1, p < 0.001). ~~This finding aligns with a study in post-war Liberia, where women in rural~~
404 ~~areas had low odds of attending at least four ANC visits and utilizing formal institutional delivery services~~
405 ~~[38]. Additionally,~~ Sstudies from Yemen reported substantial barriers for pregnant women in remote, rural
406 areas trying to access ANC clinics, primarily due to infrastructure damage, lack of public transportation,
407 and financial constraints [40~~39~~,41~~0~~]. Studies from North east Ethiopia also showed that due to facility
408 damage and medical personnel evacuation, access to healthcare is seriously jeopardized in crisis situations,
409 resulting in longer travel times and fewer services available[42].Another study quality of health care
410 service in conflict setting showed that healthcare disruption in armed conflict creates major obstacles to
411 the provision of healthcare, such as medical supply shortages and access-hindering security threats(43).On
412 the other hand, although distance is an important consideration, socioeconomic position, education, and
413 community support are also important factors that affect healthcare continuity and access in areas affected
414 by violence.[44]
415 Geographic zones also played a critical role; women living in the south-east zone had a 0.11~~876~~ lower log-

**Formatted:** Line spacing: 1.5 lines

416 odds of discontinuing ANC compared to those in the north-west zone (SE = 0.05~~0501~~, p = 0.01~~8685~~).
417 Similarly, women in the eastern zone exhibited a 0.16~~452~~ lower log-odds of discontinuing ANC compared
418 to the north-west zone (SE = 0.04~~8012~~, p = 0.00~~0611~~1). Thus, living in the north-west zone was associated
419 with a higher likelihood of ANC discontinuation in the Tigray region. This finding mirrors those from the
420 Eastern Mediterranean region, where conflicts have disrupted health service utilization, especially in rural
421 areas [45~~1~~]. A study in the Democratic Republic of the Congo noted that pregnant women displaced to
422 rural areas had significantly lower ANC attendance rates than those remaining in or fleeing to urban
423 centers [46~~2~~,47~~3~~]. Furthermore, research in Afghanistan showed that the Taliban's restrictions on women's
424 movement disproportionately affected rural women, leading to higher rates of missed ANC appointments
425 compared to their urban counterparts [48~~4~~,49~~5~~,47~~6~~,50,51~~47~~].
426 These findings underscore the critical importance of addressing the unique challenges faced by pregnant
427 women in war-affected regions, particularly those living in remote, rural areas with limited access to
428 health facilities. Enhancing transportation, infrastructure, and security in these areas is essential for
429 improving maternal healthcare utilization and outcomes.
430 Broader patterns in conflict-affected settings reveal that the uneven distribution of conflict intensity and
431 damage to health infrastructure across different zones has led to varying impacts on pregnant women's
432 ability to receive antenatal care (ANC). The findings from this study in Tigray align with another analysis
433 indicating that, as of June 2021, only 27.5% of hospitals, 17.5% of health centers, 11% of ambulances, and
434 none of the 712 health posts were functional [52~~48~~].
435 While the specific reasons for the higher ANC interruption in the north-west zone of Tigray were not

**Formatted:** Left, Space Before: 1.45 pt, After: 0 pt, Line spacing: 1.5 lines, No widow/orphan control, Don't adjust space between Latin and Asian text, Don't adjust space between Asian text and numbers

436 detailed, further contextual analysis is needed to understand the underlying drivers of this geographic
437 disparity. Additionally, the number of children under five in the household significantly influenced ANC
438 continuity; for each additional child, the log-odds of discontinuing ANC increased by 0.083134 (SE =
439 0.017295, p < 0.001). Parity, and the interruption ANC visits were positively correlated, according to
440 demographic and health surveys conducted in eight Sub-Saharan African (SSA) nations, especially for
441 women with five or more children. According to our research, mothers who have more children are also
442 more likely to stop receiving ANC care. This implies that moms' capacity to prioritize their own care may
443 be hampered by the responsibilities of raising several children. Regular ANC visits seem to be
444 overshadowed by the strain of caring duties, which could endanger the health of mothers and children
445 (53)Another study conducted in Tanzania, also found that women with higher parity often rely on their previous

**Formatted:** Font: (Default) Times New Roman

446 pregnancy experiences, leading to a tendency to underutilize ANC services. This reliance on past experiences may
447 create a false sense of security, causing these women to deem ANC less critical (54). However, the practical
448 challenges of managing several young children further complicate their ability to seek necessary prenatal care,
449 reinforcing the findings of the SSA survey and the present studies.Additionally, research from Liverpool studied the
450 unique challenges faced by women experiencing multiple pregnancies. This study emphasized that increased anxiety
451 and practical obligations hinder their capacity to prioritize ANC visits (55). All those findings underscore as the
452 number of children increased the interruption of the care increasese .This showed that mothers are over whelmed by
453 the care of children thus our ANC care contacts need to be tailored to support in an individualized care strategies to
454 address the specific needs of women, ensuring that they receive adequate prenatal care.
455 This trend may reflect the additional caregiving responsibilities that pregnant mothers with multiple young

**Formatted:** Line spacing: 1.5 lines

456 children face, which can hinder their ability to prioritize their own ANC visits.
457 This may be severe in the condition of conflict where health facilities were closed due to war, health
458 personnel displaced as shown by community-based study in armed conflict in Tigray showed that only
459 36.5%, respondents reported the use of optimal antenatal care (ANC) [56].

460

**Formatted:** Line spacing: 1.5 lines

461 Moreover, household radio ownership emerged as an important factor; households without a radio had a
462 0.08939 lower log-odds of discontinuing ANC compared to those with a radio (SE = 0.033~~425~~, p =
463 0.007~~489~~). This suggests that access to media and information sources, such as radio, can positively
464 influence ANC utilization, as mothers who receive ANC-related information are more likely to act on it. A
465 studies of antenatal care and skilled birth in the fragile and conflict-affected situation of Burundi indicated
466 that women who engage with media, including radio, are more likely to utilize healthcare services this is
467 similar to present study mothers who own radio did not interrupt ANC visits this may be the information
468 disseminated through radio may be indicating where the services is available and also information about
469 security issues about war may be disseminated so us mothers know when to visit health facilities and when
470 not to visit health facilities(57).
471 Another study conducted on Antenatal care utilization and determinants in fragile and conflict-affected
472 situations in northern Ethiopia showed that radio can foster community discussions around maternal
473 health, increasing awareness and reducing stigma associated with seeking ANC in conflict zones thus in
474 conflict affecting zones radio may be an appropriate massage or information transmission methods that
475 can support health services utilization of communities in conflict affecting situations (58).
476 Another study conducted in political unrest Nepal showed that In areas where physical access to
477 healthcare is limited, radio can bridge the gap by informing women about available services and how to
478 access them, potentially increasing ANC visits this is another evidences that support the present study thus
479 owning radio in such conflict situation may get information that support where and when to visit health
480 facilities so as you get health services(59)
481 The Tigray study offers a more granular, zone-level analysis that complements zonal-level findings from
482 other conflict-affected contexts, highlighting the importance of understanding subnational variations in
483 healthcare access during conflicts. Further research to unpack the underlying drivers of geographic
484 disparities in ANC interruption in Tigray would be invaluable for informing more targeted interventions.
485 Strength and limitation of the study
486 Spatial modeling offers significant strengths in studying antenatal care interruption, particularly through
487 its ability to provide clear visual representations of geographic patterns. This clarity helps stakeholders
488 identify areas with high rates of interruption, facilitating a better understanding of the spatial dynamics of
489 healthcare access. Furthermore, these models allow researchers to incorporate a range of socio-economic,
490 environmental, and conflict-related variables, leading to a comprehensive understanding of the factors
491 influencing antenatal care. By pinpointing hotspots, health authorities can strategically allocate resources
492 and design targeted interventions, enhancing the potential for improved access and outcomes in affected
493 regions. Additionally, spatial modeling enables the tracking of changes over time, revealing trends related
494 to ongoing conflicts or recovery efforts, which is crucial for understanding the dynamics of healthcare
495 access in fluctuating environments. The insights gained from these models can also inform policymakers
496 about the specific needs of affected populations, guiding the development of effective health service
497 delivery strategies.However, spatial modeling also faces notable limitations. In war-torn regions, the
498 quality and availability of data can be compromised, leading to incomplete or biased datasets that
499 undermine the reliability of the models. Complex health outcomes are influenced by multiple
500 interdependent factors that may not be fully captured, limiting the explanatory power of the models.
501 Moreover, the assumptions and simplifications inherent in spatial modeling can oversimplify the intricate
502 realities of healthcare access, potentially resulting in misinterpretations of the data. Rapid changes in
503 conflict situations may outperform the data used in models, diminishing their relevance over time. Lastly,
504 findings derived from specific regions may not be generalizable to other contexts, which restricts the
505 broader applicability of results and may hinder the development of universal strategies for improving
506 antenatal care access

## 507 Conclusion and recommendations

508 The study identified a high prevalence of antenatal care (ANC) interruption, ~~with nearly one in four~~
509 ~~women (24.8%) facing challenges in maintaining consistent attendance.~~ Key factors associated with the
510 likelihood of discontinuing ANC included greater distance from health centers, residing in the north-west
511 zone compared to the south-east and eastern zones, having a higher number of children under five in the
512 household, and household ownership of a radio.
513 To address these findings, several strategies are recommended. First, improving access to health centers
514 could be achieved by establishing more community-based ANC services or implementing transportation
515 assistance programs. Second, targeted interventions are needed for ~~the high~~high -risk north-west zone to
516 specifically address the elevated rates of ANC interruption in this region. Third, providing additional
517 resources and support, such as childcare assistance or conditional cash transfers, could benefit households
518 with multiple young children, helping to alleviate caregiving burdens.
519 Promoting the use of radio and other mass media channels is also crucial for disseminating information
520 and encouraging ANC attendance, particularly in households ~~that own~~ with a radio. Additionally, further
521 research is essential to unpack the underlying drivers of geographic disparities in ANC interruption in
522 Tigray, which would inform more targeted and effective interventions. By addressing these factors, it may
523 be possible to enhance the continuity
524 of antenatal care and ultimately improve maternal and child health outcomes in the Tigray region

525

526 The findings indicate significant interruptions in antenatal care (ANC) in Tigray, Driven by geographic
527 disparities, household dynamics, and access to information. Specifically, the increased log-odds of
528 interruptions with additional travel time highlight the urgent need for improved infrastructure and security.
529 The lower log-odds of interruption in the southeast and eastern zones suggest that targeted interventions
530 could be beneficial in areas most affected. Furthermore, the association between household dynamics and
531 ANC interruptions emphasizes the importance of enhancing mass media health information dissemination
532 to improve access to care. Therefore, further research on geographic disparities is essential to inform
533 tailored strategies for improving ANC services in the region.

## 534 Ethical considerations

535 Ethical clearance was approved by the Tigray Health Research Institute review board with reference
536 number (THRI 4031/1099/15). A permission letter was obtained from the Tigray regional health bureau.
537 Oral consent and volunteer participation were obtained from study participants after fully communicated
538 the benefits and risks of participation in the study with a right to discontinue participation at any stage of
539 the interview. Data confidentiality was maintained throughout the research process.In conducting our
540 research with vulnerable populations in conflict zones, we prioritize several ethical considerations to
541 ensure the integrity and safety of the study. Informed consent is fundamental; we ensure that participants
542 fully understand the study's purpose, risks, and benefits by simplifying the language to Tigrigna and
543 providing culturally relevant information. Safety and security are paramount, so we assess potential risks
544 associated with participation and implement measures to mitigate them, safeguarding participants,
545 researchers, and the community. We also maintain confidentiality by protecting participants' identities and
546 personal information through anonymization techniques and secure data storage, particularly in sensitive
547 contexts. Our approach is guided by cultural sensitivity, recognizing and respecting local norms and
548 values, and actively engaging with communities to ensure our methods are appropriate. We strive for
549 beneficence and non-maleficence, aiming to maximize benefits while minimizing harm, considering how
550 our research outcomes can positively impact the community. Community engagement is crucial; we
551 involve local stakeholders and organizations, such as health facility workers, in the data collection process
552 to enhance the relevance and acceptance of our research. Additionally, we plan for post-study benefits,
553 ensuring that findings are shared with participants and the community so they can directly benefit from the
554 results. Finally, we have obtained approval from an ethics review board, acknowledging the unique
555 challenges posed by conflict situations and reinforcing our commitment to ethical research practices.

## 556 Acknowledgements

557	We extend our heartfelt gratitude to the Tigray Health Bureau (THB) for leading this integrated research

558	and mobilising technical staff for data collection. Lastly, we thank the participants, as well as the data

559	collectors and supervisors, particularly those from the Mekelle University College of Health Sciences and

560	Tigray Health Research Institute (THRI), for their commitment and integrity in generating quality data.

561	Conflicts of interests

562 In the research on antenatal care interruption in Tigray, we maintain a strong commitment to avoiding
563 conflicts of interest. We ensure that our funding sources do not create biases, as we do not have any
564 conflicts with organizations that might have vested interests in the outcomes, such as government agencies
565 or NGOs with specific agendas. Our collaborative relationships with local health authorities and NGOs are
566 structured to prevent any conflicts, as these entities do not have a stake in the research findings that could
567 influence our work. Additionally, the researchers do not have personal ties to the region or its healthcare
568 systems that could compromise the interpretation of data or reporting results. We are also vigilant against
569 publication bias, ensuring that we do not face conflicts that would pressure us to publish only positive
570 outcomes aligned with funding interests, which could distort the integrity of our research. Ethical
571 considerations are paramount; we adhere to rigorous ethical guidelines to address the unique challenges of
572 working with vulnerable populations in conflict zones, recognizing the dilemmas that may arise. To
573 mitigate potential conflicts, we prioritize transparency by disclosing all funding sources and potential
574 conflicts in our publications. We are committed to an independent review process, welcoming input from
575 independent experts in study design and data analysis to ensure objectivity. Finally, we uphold ethical
576 oversight by obtaining approval from relevant review boards Tigray health research institute (THRI) as
577 mentioned above to safeguard participant welfare, reinforcing our dedication to conducting responsible
578 and ethical research.
579 **Authors’ Contributions:**
580 A. A., M. A., H. G., B A. , H. G ,and A. M. B ., conceived and designed the experiments and analyzed
581 and write up the report ., F. H., T. W., A. A., A. A. M., Z. H. K., L. M., M. K. C., R. S., M. A., H. K., T.
582 H., F. G., A. A., K. D., M. T., M. W., A. G., A. A. D., G. G., A. H., R. E., T. B., A. G., T. T., A. A., A. M.
583 B. performed the experiments and approved and revised the final manuscript

584	**Reference**

585 1. WHO, UNICEF, UNFPA and TheWorld Bank. Trends inMaternalMortality: 2000 to 2017WHO.
586 Geneva:WHO (2019).
587 2. Tunçalp Ö, Pena-Rosas JP, Lawrie T, et al. WHO Recommendations on antenatal care for a positive
588 pregnancy experience-going beyond survival. Bjog. 2017;124(6):860-862.
589 3. O’hare BAM, David PS. First do no harm: the impact of recent armed conflict on maternal and child

590	health in Sub-Saharan Africa. J R Soc Med. (2007) 100:564–70. doi: 10.1258/jrsm.100.12.564

| 591  592  593 | ~~4. DeJong J, Ghattas H, Bashour H, Mourtada R, Akiki C, Reese_MastersonA. Reproductive, maternal,~~ ~~neonatal and child health in conflict: a case~~  4. ~~study on Syria using countdown indicators. BMJ Global Health. (2017) 2:e000302. doi:~~ |
| --- | --- |
| 594 | ~~10.1136/bmjgh-2017-000302~~ DeJong J, Ghattas H, Bashour H, Mourtada R, Akiki C, |
| 595 | Reese_MastersonA. Reproductive, maternal, neonatal and child health in conflict: a case study on |
| 596 | Syria using countdown indicators. BMJ Global Health. (2017) 2:e000302. doi: 10.1136/bmjgh-2017- |
| 597 | 000302 |
| 598 |  |
| 599 | 5. Akseer N, Wright J, Tasic H, Everett K, Scudder E, Amsalu R, et al. Women, children and adolescents |
| 600 | in conflict countries: an assessment of inequalities in intervention coverage and survival BMJ Global |
| 601 | Health. (2020) 5:e002214. doi: 10.1136/bmjgh-2019-002214 |
| 602 | ~~5. Akseer N, Wright J, Tasic H, Everett K, Scudder E, Amsalu R, et al. Women, children and adolescents~~ |
| 603 | ~~in conflict countries: an assessment of~~ |
| 604 | ~~inequalities in intervention coverage and survival BMJ Global Health. (2020) 5:e002214. doi:~~ |
| 605 | ~~10.1136/bmjgh-2019-002214~~ |
| 606 | 6. Mullany LC, Lee CI, Yone L, Paw P, Oo EKS, Maung C, et al. Access to essential maternal health |
| 607 | interventions and human rights violations among vulnerable communities in Eastern Burma. PLoS |
| 608 | Med. (2008)5: e242. doi: 10.1371/journal.pmed.0050242 |

609	7. Central Statistical Agency (CSA) [Ethiopia] and ICF. Ethiopia Demographic and Health Survey 2016. Addis Ababa, Ethiopia,

610	and Rockville, Maryland, USA: CSA and ICF. 2016

611	~~7. ETHIOPIA Demographic and Health Survey Central Statistical Agency Addis Ababa, Ethiopia The~~

612	~~DHS Program ICF Rockville, Maryland, USA, 2016~~

613	8.

614 9. Abay ST, Gebre-Egziabher AG. Status and associated factors of birth registration in selected districts of Tigray
615 region, Ethiopia. BMC international health and human rights. 2020 Dec;20:1-0.
616 ~~8. Abay, S.T., Gebre-egziabher, A.G. Status and associated factors of birth registration in selected~~
617 ~~districts	of	Tigray	region,	Ethiopia.~~ *~~BMC	Int	Health	Hum	Rights~~* **~~20~~**~~,	20	(2020).~~
618 ~~https://doi.org/10.1186/s12914-020-00235-x~~
619 ~~9.~~10. Godefay H, Byass P, Graham WJ, Kinsman J, Mulugeta A. Risk factors for maternal mortality in
620 rural Tigray, northern Ethiopia: a case-control study. PloS one. 2015 Dec 17;10(12):e0144975.
621 ~~10.~~11. Legesse AY, Teka Tseghay H, Abraha HE, Fisseha G, Ebrahim MM, Tsadik M, Berhe B,
622 Gebrekurstos G, Ayele B, Gebremeskel T, Gebremariam T. Maternal mortality during war time in
623 Tigray, Ethiopia: A community‐based study. BJOG: An International Journal of Obstetrics &
624 Gynaecology. 2024 May;131(6):786-94.
625 ~~11.~~12. Namasivayam, Amrita, González, Pedro Arcos, Delgado, Rafael Castro, Chi,Primus Che. The
626 effect of armed conflict on the utilization of maternal health services in Uganda: a population-based
627 study.	PLoS	Curr	Disast.	(2017)	9:1689–98.	doi:

| 628 | 10.1371/currents.dis.557b987d6519d8c7c96f2006ed3c271a |
| --- | --- |
| 629 | ~~12.~~13. Kabakian-Khasholian, Tamar, Shayboub, Rawan, El-Kak, Faysal. Seeking maternal care at times |
| 630 | of conflict: the case of Lebanon. Health Care Women Int. (2013) 34:352–62. doi: |
| 631 | 10.1080/07399332.2012.736570 |
| 632 | ~~13.~~14. Kabakian-Khasholian, Tamar, Shayboub, Rawan, El-Kak, Faysal. Seeking maternal care at times |
| 633 | of conflict: the case of Lebanon. Health Care Women Int. (2013) 34:352–62. doi: |
| 634 | 10.1080/07399332.2012.736570 |
| 635 | ~~14.~~15. Chi PC, Bulage P, Urdal H, Sundby J. Perceptions of the effects of armed conflict on maternal and |
| 636 | reproductive health services and outcomes inBurundi and Northern Uganda: a qualitative study. BMC |
| 637 | Int Health Hum Rights. (2015) 15:7. doi: 10.1186/s12914-015-0045-z |
| 638 | ~~15.~~16. Indicators K. Mini demographic and health survey. EPHI and ICF. 2019 Jul. |
| 639 | ~~16.~~17. : Gesesew H, Berhane K, Siraj ES, et al. The impact of war on the health system of the Tigray |
| 640 | region in Ethiopia: an assessment. BMJ Global Health 2021;6: e007328. doi:10.1136/ bmjgh-2021- |
| 641 | 007328 |
| 642 | ~~17.~~ Alemayehu M, Demoz K, Yahya M, Teamir A, Asmelash A, Gebrekurstos G, et al. Does integration |
| 643 | of health survey bring cost effectiveness into evidence generation in post conflict settings of Tigray, |
| 644 | Ethiopia?. East Afr J Health Sci. 2024;6(1):798-808. doi: [10.71624/p8bzch81](https://doi.org/10.71624/p8bzch81)~~Mussie Alemayehu etal,~~ |
| 645 | ~~does integration of surveys bring cost effectiveness into evidence generation in post conflict settings of~~ |

646	~~Tigray, Ethiopia? East African journal of Health sciences ORCID: 0000-0002-0035-631X~~

647	~~https://journal.mu.edu.et/index.php/eajhs/issue/view/56~~

648	18. Paciorek CJ. Computational techniques for spatial logistic regression with large data sets. Comput Stat

649	Data Anal. 2007;51(8):3631-53. doi:10.1016/j.csda.2006.11.008.

650	19. Baddeley A, Berman M, Fisher N, et al. Spatial logistic regression and change-of-support in Poisson

651	point processes. Electron J Stat. 2010;4:1151-201. doi:10.1214/10-ejs581.

652 20. Bihrmann K, Toft N, Nielsen SS, et al. Spatial correlation in Bayesian logistic regression with
653 misclassification. Spatial Spatio-temporal Epidemiol. 2014;9:1-12. doi:10.1016/j.sste.2014.02.002
654 21. World Health Organization. WHO recommendations on antenatal care for a positive pregnancy
655 experience: summary: highlights and key messages from the World Health Organization's 2016 global
656 recommendations for routine antenatal care. World Health Organization; 2018.
657 22. Ekzayez A, Alhaj Ahmad Y, Alhaleb H, Checchi F. The impact of armed conflict on utilisation of
658 health services in north-west Syria: an observational study. Conflict and health. 2021 Dec; 15:1-0.
659 23. Stanikzai MH, Tawfiq E, Jafari M, Wasiq AW, Seddiq MK, Currie S, Sayam H, Baray AH, Saeedzai
660 SA. Contents of antenatal care services in Afghanistan: findings from the national health survey 2018.
661 BMC Public Health. 2023 Dec 11;23(1):2469.
662 24. Glass N, Jalalzai R, Spiegel P, Rubenstein L. The crisis of maternal and child health in Afghanistan.
663 Conflict and health. 2023 Jun 12;17(1):28.
664 25. Saleem SM, Shoib S, Dazhamyar AR, Chandradasa M. Afghanistan: decades of collective trauma,
665 ongoing humanitarian crises, Taliban rulers, and mental health of the displaced population. Asian
666 journal of psychiatry. 2021 Nov 1; 65:102854.
667 26. Zhang T, He Q, Richardson S, Tang K. Does armed conflict lead to lower prevalence of maternal
668 health-seeking behaviours: theoretical and empirical research based on 55 683 women in armed
669 conflict settings. BMJ Global Health. 2023 Aug 1;8(8): e012023.
670 27. World Health Organization. WHO’s response, and role as the health cluster lead, in meeting the
671 growing demands of health in humanitarian emergencies [Internet]. Sixty-Fifth World Health
672 Assembly. 2012 May:21-6.
673 28. Mourtada R, Bashour H, Houben F. A qualitative study exploring barriers to adequate uptake of
674 antenatal care in pre-conflict Syria: low-cost interventions are needed to address disparities in
675 antenatal care. Contraception and Reproductive Medicine. 2021 Jun 1;6(1):17.
676 29. Basha S, Socarras A, Akhter MW, Hamze M, Albaik A, Hussein I, Tarakji A, Hamadeh M, Loutfi R,
677 Kewara M, Alahdab F. Protracted armed conflict and maternal health: a scoping review of literature
678 and a retrospective analysis of primary data from northwest Syria. BMJ Global Health. 2022 Aug
679 1;7(8): e008001.
680 30. Nabulsi D, Abou Saad M, Ismail H, Doumit MA, El-Jamil F, Kobeissi L, Fouad FM. Minimum initial
681 service package (MISP) for sexual and reproductive health for women in a displacement setting: a
682 narrative review on the Syrian refugee crisis in Lebanon. Reproductive Health. 2021 Dec; 18:1-3.
683 31. Mesfin, B., Demise, A. M., Shiferaw, M., Gebreegziabher, F., & Girmaw, F. (2023). The Effect of
684 Armed Conflict on Treatment Interruption, Its Outcome and Associated Factors Among
685 Chronic Disease Patients in North East, Amhara, Ethiopia, 2022. *Patient Related Outcome*
686 *Measures*, *14*, 243–251. <https://doi.org/10.2147/prom.s388426>
687 32. Tessema, Z. T., & Akalu, T. Y. (2020). Spatial Pattern and Associated Factors of ANC Visits in
688 Ethiopia: Spatial and Multilevel Modeling of Ethiopian Demographic Health Survey
689 Data. *Advances in Preventive Medicine*, *2020*, 4676591. [https://doi.org/10.1155/2020/4676591.](https://doi.org/10.1155/2020/4676591)
690 33. 33-Akseer, N., et al. (2020). Progress in maternal and child health: How has Afghanistan fared in the
691 last decade? *The Lancet Global Health*, 8(3), e351–e360.
692 34. Qirbi N, Ismail SA. Health system functionality in a low-income country in the midst of conflict: the
693 case of Yemen. Health policy and planning. 2017 Jul 1;32(6):911-22.
694 35. Hyzam D, Zou M, Boah M, Saeed A, Li C, Pan S, Zhai J, Wu LJ. Health information and health-
695 seeking behaviour in Yemen: perspectives of health leaders, midwives and mothers in two rural areas
696 of Yemen. BMC Pregnancy and Childbirth. 2020 Dec; 20:1-2.
697 36. Stamidis K, Bologna L, Losey L. CORE Group Polio Project (CGPP) Final Evaluation Report 2017.
698 37. Zhang T, Qi X, He Q, Hee J, Takesue R, Yan Y, Tang K. The effects of conflicts and self-reported
699 insecurity on maternal healthcare utilisation and children health outcomes in the democratic republic
700 of congo (Drc). InHealthcare 2021 Jul 3 (Vol. 9, No. 7, p. 842). MDPI.
701 38. Bwirire D, Roosen I, De Vries N, Letschert R, Ntabe Namegabe E, Crutzen R. Maternal Health Care
702 Service Utilization in the Post-Conflict Democratic Republic of Congo: An Analysis of Health
703 Inequalities over Time. InHealthcare 2023 Oct 31 (Vol. 11, No. 21, p. 2871). MDPI.
704 39. Ormhaug CM. Effects of civil war on maternal and child health care in sub-Saharan Africa 2014
705 40. Ziegler BR. *Pregnancy in Peril: the impact of conflict on antenatal care and skilled birth attendant*
706 *utilization in the Democratic Republic of the Congo and Burundi* (Doctoral dissertation, The
707 University of Western Ontario (Canada))2020.
708 41. Yaya S, Uthman OA, Bishwajit G, Ekholuenetale M. Maternal health care service utilization in post-
709 war Liberia: analysis of nationally representative cross-sectional household surveys. BMC Public
710 Health. 2019 Dec;19:1-2
711 42. Asah, M., & Moses, A. L. (2025). The Effects of Armed Conflict on Healthcare in Nigeria – A
712 Scoping Review. *International Journal of Research and Scientific Innovation*, *XI*(XV), 883–900.
713 https://doi.org/10.51244/ijrsi.2024.11150067p
714 43. Ikpongifono, U. (2024). Enhancing Healthcare Access in Conflict Zones: Identifying Challenges and
715 Proposing Solutions. *Public Health Open Access*, *8*(1). https://doi.org/10.23880/phoa-16000286
716 44. Mavole, J. (2023). Protection and Maintenance of Healthcare Services for Civilians’ Well-Being in
717 Conflict Affected Areas: Comparative Analysis of The African Situation. *European Journal of Health*
718 *Sciences*, *9*(2), 1–17. https://doi.org/10.47672/ejhs.1507
719 45. Grainger, C.G., Gorter, A.C., Al-Kobati, E. *et al.* Providing safe motherhood services to underserved
720 and neglected populations in Yemen: the case for vouchers. *Int J Humanitarian Action* **2**, 6 (2017).
721 <https://doi.org/10.1186/s41018-017-0021-4>
722 46. Sheline A. The Yemen War in Numbers: Saudi Escalation and US Complicity. Quincy Institute of US
723 Complicity. 2022 Mar 21
724 47. GOVERNMENT ACCOUNTABILITY OFFICE WASHINGTON DC. Uncertain Political and
725 Security Situation Challenges US Efforts to Implement a Comprehensive Strategy in Yemen. 2012
726 48. 48. Karakır İA. Ongoing conflict in Yemen: A proxy war Tesam Akademi Dergisi. 2018;5(2):121-49.
727 49. Krug EG, Mercy JA, Dahlberg LL, Zwi AB. The world report on violence and health. The lancet. 2002

728	Oct 5;360(9339):1083-8.

729 50. Alibhai KM, Ziegler BR, Meddings L, Batung E, Luginaah I. Factors impacting antenatal care
730 utilization: a systematic review of 37 fragile and conflict-affected situations. Confl Health. 2022 Jun
731 11;16(1):33. doi: 10.1186/s13031-022-00459-9. PMID: 35690840; PMCID: PMC9188725
732 51. Wake SK, Botore A, Mohammed A, Gemede K, Bariso M, Gerema U. Disparities in Antenatal Care
733 Visits between Urban and Rural Ethiopian Women. J Pregnancy. 2023 Sep 27; 2023:9031344. doi:
734 10.1155/2023/9031344. PMID: 37799709; PMCID: PMC10550413.
735 52. Ahmadi A. Assessing Participation in Women's Development Projects in Afghanistan 2017
736 53. Saqib M. *Afganistan’da Devlete Yapılanan Kusurlar: Afganistan’ın 2004 Anayasasına Göre Yerel*
737 *Seçimlerinin Kritik Bir Analizi* (Doctoral dissertation, Marmara Universitesi (Turkey)). 2022
738 54. Gesesew H, Berhane K, Siraj ES, et al. The impact of war on the health system of the Tigray region in
739 Ethiopia: an assessment. BMJ Global Health 2021;6: e007328. doi:10.1136/ bmjgh-2021-007328

**Formatted:** Font: Times New Roman, 12 pt, Italic

**Formatted:** Normal, Line spacing: 1.5 lines, No bullets or numbering, Adjust space between Latin and Asian text, Adjust space between Asian text and numbers

**Formatted:** Font: Italic

740 55. Odusina, E. K., Ahinkorah, B. O., Ameyaw, E. K., Seidu, A.-A., Seidu, A.-A., Budu, E., Zegeye, B.,
741 & Yaya, S. (2021). Noncompliance with the WHO’s Recommended Eight Antenatal Care Visits
742 among Pregnant Women in Sub-Saharan Africa: A Multilevel Analysis. BioMed Research
743 International, 2021, 6696829. <https://doi.org/10.1155/2021/6696829>
744 56. Owen, D. J., Wood, L., & Neilson, J. P. (2004). Antenatal care for women with multiple pregnancies:
745 the	Liverpool	approach.	[Clinical	Obstetrics	and	Gynecology, 47(1),	263–271.](https://doi.org/10.1097/00003081-200403000-00026)
746 <https://doi.org/10.1097/00003081-200403000-00026>
747 57. Gupta, S., Yamada, G., Mpembeni, R., Frumence, G., Callaghan-Koru, J. A., Stevenson, R., Brandes,
748 N., & Baqui, A. H. (2014). Factors Associated with Four or More Antenatal Care Visits and Its
749 Decline among Pregnant Women in Tanzania between 1999 and 2010. [PLOS ONE, 9(7).](https://doi.org/10.1371/JOURNAL.PONE.0101893)
750 <https://doi.org/10.1371/JOURNAL.PONE.0101893>
751 58. Tsadik M, Teka H, Gebremichael MW, Gebretnsae H, Yemane A, Fisseha G, Gebregzabher T, Buruh
752 G, Gebresellasie F, Tekie M, Kiros G. Armed conflict and maternal health service utilization in
753 Ethiopia’s Tigray Region: a community-based survey. BMC public health. 2024 Oct 16;24(1):2840.
754 59. Ziegler, B. R., Kansanga, M. M., Sano, Y., Sano, Y., Kangmennaang, J., Kpienbaareh, D., &
755 Luginaah, I. (2021). Antenatal care and skilled birth in the fragile and conflict-affected situation of
756 Burundi International Journal of Health Planning and Management, 36(4), 1081–1106.
757 https://doi.org/10.1002/HPM.3157
758 60. Gedef, G. M., Girma, B., Tibebu, N. S., Gashaw, A., & Andualem, F. (2023). Antenatal care
759 utilization and determinants in fragile and conflict-affected situations in Sekota zuria district, northern
760 Ethiopia,	2022:	a	community-based	cross-sectional	study. Midwifery.
761 <https://doi.org/10.1016/j.midw.2023.103906>
762 ~~61.~~ Price, J. I., & Bohara, A. K. (2013). Maternal health care amid political unrest: the effect of armed
763 conflict on antenatal care utilization in Nepal. *Health Policy and Planning*, *28*(3), 309–319.
764 <https://doi.org/10.1093/HEAPOL/CZS062>

765	*61.*

1	~~62.~~

Response to Reviewers

### Response to editor and Reviewers

PLOS ONE

Thank you for the opportunity to revise our manuscript titled "Spatial Patterns and Predictors of Antenatal Care Interruption in War-Torn Tigray, Northern Ethiopia: Spatial Modeling Approach Manuscript ID: PONE-D-25-08979. We appreciate the constructive feedback from you and the reviewers. Below, we address each comment in detail.

2. We note that the grant information you provided in the ‘Funding Information’ and ‘Financial Disclosure’ sections do not match.

When you resubmit, please ensure that you provide the correct grant numbers for the awards you received for your study in the ‘Funding Information’ section.

**Response:** our funding had not grant number because the budget was utilizing through perdiem .

. Thank you for stating the following financial disclosure:

[UNICEF, WHO, Amref Health Africa and UNFPA and Tigray regional heal bureau and Mums for Mums (MfM) for all rounded support in ensuring a successful and productive integrated research data collection process we assure there is no any competing programming and any other support that influences the research output]. The funders had no role in study design, data collection and analysis, decision to publish, or preparation of the manuscript Please state what role the funders took in the study. If the funders had no role, please state: **Response thank you for your comment we have revised as you suggested**

UNICEF, WHO, Amref Health Africa and UNFPA and Tigray regional heal bureau and Mums for Mums (MfM) for all rounded support in ensuring a successful and productive integrated research data collection process we assure there is no any competing programming and any other support that influences the research output. The funders had no role in study design, data collection and analysis, decision to publish, or preparation of the manuscript

**In** the research on antenatal care interruption in Tigray, we maintain a strong commitment to avoiding conflicts of interest. We ensure that our funding sources do not create biases, as we do not have any conflicts with organizations that might have vested interests in the outcomes, such as government agencies UNICEF, WHO, Amref Health Africa and UNFPA and Tigray regional

heal bureau and Mums for Mums (MfM with specific agendas. Our collaborative relationships with local health authorities and NGOs are structured to prevent any conflicts, as these entities do not have a stake in the research findings that could influence our work. Additionally, the researchers do not have personal ties to the region or its healthcare systems that could compromise the interpretation of data or reporting results. We are also vigilant against publication bias, ensuring that we do not face conflicts that would pressure us to publish only positive outcomes aligned with funding interests, which could distort the integrity of our research. Ethical considerations are paramount; we adhere to rigorous ethical guidelines to address the unique challenges of working with vulnerable populations in conflict zones, recognizing the dilemmas that may arise. To mitigate potential conflicts, we prioritize transparency by disclosing all funding sources and potential conflicts in our publications. We are committed to an independent review process, welcoming input from independent experts in study design and data analysis to ensure objectivity. Finally, we uphold ethical oversight by obtaining approval from relevant review boards Tigray health research institute (THRI) as mentioned above to safeguard participant welfare, reinforcing our dedication to conducting responsible and ethical research

4. Thank you for stating the following in the Acknowledgments Section of your manuscript: [We extend our heartfelt gratitude to the Tigray Health Bureau (THB) for leading this integrated research and mobilizing technical staff for data collection. Lastly, we thank the participants, as

well as the data collectors and supervisors, particularly those from Mekelle University College of Health Sciences and Tigray Health Research Institute (THRI), for their commitment and integrity in generating quality data.]

We note that you have provided funding information that is not currently declared in your Funding Statement. However, funding information should not appear in the Acknowledgments section or other areas of your manuscript. We will only publish funding information present in the Funding Statement section of the online submission form.

Please remove any funding-related text from the manuscript and let us know how you would like to update your Funding Statement. Currently, your Funding Statement reads as follows: [UNICEF, WHO, Amref Health Africa and UNFPA and Tigray regional heal bureau and Mums for Mums (MfM) for all rounded support in ensuring a successful and productive integrated

research data collection process we assure there is no any competing programming and any other support that influences the research output].

Please include your amended statements within your cover letter; we will change the online submission form on your behalf.

**Response:** thanks we have removed from the acknowledgements section

5. We note that your Data Availability Statement is currently as follows: [Data is available within the manuscript]

Please confirm at this time whether or not your submission contains all raw data required to replicate the results of your study. Authors must share the “minimal data set” for their submission. PLOS defines the minimal data set to consist of the data required to replicate all study findings reported in the article, as well as related metadata and methods (<https://journals.plos.org/plosone/s/data-availability#loc-minimal-data-set-definition>).

For example, authors should submit the following data:

- The values behind the means, standard deviations and other measures reported;
- The values used to build graphs;
- The points extracted from images for analysis.

Authors do not need to submit their entire data set if only a portion of the data was used in the reported study.

If your submission does not contain these data, please either upload them as Supporting Information files or deposit them to a stable, public repository and provide us with the relevant URLs, DOIs, or accession numbers. For a list of recommended repositories, please

see [https://journals.plos.org/plosone/s/recommended-repositories.](https://journals.plos.org/plosone/s/recommended-repositories)

If there are ethical or legal restrictions on sharing a de-identified data set, please explain them in detail (e.g., data contain potentially sensitive information, data are owned by a third-party organization, etc.) and who has imposed them (e.g., an ethics committee). Please also provide contact information for a data access committee, ethics committee, or other institutional body to which data requests may be sent. If data are owned by a third party, please indicate how others may request data access.

Response: Thank you we will submit the data set if our manuscript will be accepted

6. Please amend either the title on the online submission form (via Edit Submission) or the title in the manuscript so that they are identical.

**Response:** Thank you for your comment we have made identical

7. Your ethics statement should only appear in the Methods section of your manuscript. If your ethics statement is written in any section besides the Methods, please move it to the Methods section and delete it from any other section. Please ensure that your ethics statement is included in your manuscript, as the ethics statement entered into the online submission form will not be published alongside your manuscript.

**Response** ;it is only in the methods sections

8. Please review your reference list to ensure that it is complete and correct. If you have cited papers that have been retracted, please include the rationale for doing so in the manuscript text, or remove these references and replace them with relevant current references. Any changes to the reference list should be mentioned in the rebuttal letter that accompanies your revised manuscript. If you need to cite a retracted article, indicate the article’s retracted status in the References list and also include a citation and full reference for the retraction notice.

**Response**: I have added six references

31- Mesfin, B., Demise, A. M., Shiferaw, M., Gebreegziabher, F., & Girmaw, F. (2023). The Effect of Armed Conflict on Treatment Interruption, Its Outcome and Associated Factors Among Chronic Disease Patients in North East, Amhara, Ethiopia, 2022. *Patient Related Outcome Measures*, *14*, 243–251. <https://doi.org/10.2147/prom.s388426>
32- Tessema, Z. T., & Akalu, T. Y. (2020). Spatial Pattern and Associated Factors of ANC Visits in Ethiopia: Spatial and Multilevel Modeling of Ethiopian Demographic Health Survey

Data. *Advances in Preventive Medicine*, *2020*, 4676591. <https://doi.org/10.1155/2020/4676591>. 33-Akseer, N., et al. (2020). Progress in maternal and child health: How has Afghanistan fared in the last decade? *The Lancet Global Health*, 8(3), e351–e360.

42- Asah, M., & Moses, A. L. (2025). The Effects of Armed Conflict on Healthcare in Nigeria – A Scoping Review. *International Journal of Research and Scientific Innovation*, *XI*(XV), 883– 900. https://doi.org/10.51244/ijrsi.2024.11150067p
43- Ikpongifono, U. (2024). Enhancing Healthcare Access in Conflict Zones: Identifying Challenges and Proposing Solutions. *Public Health Open Access*, *8*(1). https://doi.org/10.23880/phoa-16000286
44- Mavole, J. (2023). Protection and Maintenance of Healthcare Services for Civilians’ Well- Being in Conflict Affected Areas: Comparative Analysis of The African Situation. *European Journal of Health Sciences*, *9*(2), 1–17. https://doi.org/10.47672/ejhs.1507

## General Comments

5. Review Comments to the Author

Please use the space provided to explain your answers to the questions above. You may also include additional comments for the author, including concerns about dual publication, research ethics, or publication ethics. (Please upload your review as an attachment if it exceeds 20,000 characters)

Reviewer #1: Title: Spatial patterns and predictors of antenatal care interruption in war-torn Tigray, northern Ethiopia: a spatial modeling approach

I am happy to review this manuscript, which is well-written for the “Spatial patterns and predictors of antenatal care interruption in war-torn Tigray, northern Ethiopia: spatial modeling approach” and has brought some important findings. However, I have encountered some typos error in many instances. That is proofread is needed.

- Why you used mixed methods?

**Response:** in the integrated survey it was used to collect the qualitative one but I did not use the qualitative because it is mixed up with others like COVID19 socio-behavioral drivers, other maternal health services that is why I did not use for ANC interruption.

- In Abstract: Result Part: A result should be interpreted well and written to the nearest value. It seems vague.

Response :Great I made like this

This finding underscores significant accessibility challenges posed by security checkpoints and conflict-related dangers. Geographic zone location significantly affected ANC interruptions.

Women in the southeast and eastern zones experiencing lower log-odds of interruption compared to the northwest zone. Indicating zonal disparities in access to care.

Moreover, the number of children under five years old in a household was associated with a 0.083134 increase in log-odds of ANC interruption (SE = 0.017295, p < 0.001). This suggests that larger family sizes may contribute to greater challenges in accessing ANC services.

Additionally households without a radio had a 0.08939 lower log-odds of interruption (SE =

0.033425, p = 0.007489), indicating the importance of access to information to mitigate ANC interruption.

- Conclusion of abstract, should be written again, especially recommendation part. Theoretically, recommendation should be emanated from result. Which result leads to this recommendation “Improving infrastructure and security, implementing targeted interventions, enhancing mass media health information dissemination, and further research on geographic disparities are essential.”?

Response: Thank you this is really good here I amend like this Conclusion

The findings indicate significant interruptions in antenatal care (ANC) in Tigray, Driven by geographic disparities, household dynamics, and access to information. Specifically, the increased log-odds of interruptions with additional travel time highlight the urgent need for improved infrastructure and security. The lower log-odds of interruption in the southeast and eastern zones suggest that targeted interventions could be beneficial in areas most affected. Furthermore, the association between household dynamics and ANC interruptions emphasizes the importance of enhancing mass media health information dissemination to improve access to care. Therefore, further research on geographic disparities is essential to inform tailored strategies for improving ANC services in the region.

- General comment: In introduction, it seems good. Please, rephrase and conceptualize in your own words. Basically, your introduction also should focus on a) what we already know from the literature, b) what new concepts this study can address that make a distinct contribution beyond the prior studies? People want to see the effect of war in the ANC use? Previous distribution should be written well.

Response: I tried to incorporate it is acceptable

- Methods part: Good but describe study area in connection to your title in detail.

Response: Study area: The study was conducted in Tigray region, Northern Ethiopia. Tigray borders on the north by Eretria, on the west by Sudan, on the south by Amhara, and on the east by Afar. The region has a total population of 7,969,000 []. The region’s health system consisted of two specialized comprehensive hospitals, 14 general hospitals, 24 primary hospitals, 231 health centers, and 743 health posts. The survey was conducted in selected 19 districts of the

six zones of the region (excluding western zone for security reasons). Data was collected from July 1 to 30, 2023

- Perhaps I am expecting from you a special distribution of the problem in the map. Geographical map is needed to show the existence of the problem at the end of the result as a result or at the end of the introduction as the concept of indication.

### Response:

At the end of introduction

At the end of results were added and at the introduction

The spatial distribution of ANC interruptions across the Tigray region is as follows: Northwestern zone 22.8%, Central zone 29.1%, Eastern zone 17.7%, Southern zone 16.7%, Southeastern zone 6.4%, and Mekelle zone 7.3%. However, the Western zone of Tigray was not accessible for our study due to security concerns. For more details, see Fig 1.

- Measurement and variables should be written in detail.

### Response

Study Design: Community based cross sectional study Measurements and variables

Dependent variable; antenatal care interruption:

Measured as a binary variable indicating whether ANC services were interrupted (1) or not interrupted (0) during the conflict period. This was assessed through participant’s interviews in selected households of mothers who were following ANC visit during the conflict and interrupted their care of different reasons.

Independent Variables

Independent variables include a range of socio-economic, geographical, and conflict-related factors:

Socio-economic factors: age of mother, education level, occupation, marital status, Geographical Factors: zone of residence, distance to health facility: Measured in kilometers and time taken to double trip from the home to the nearest health facility providing ANC services. Accessibility index a composite measure reflecting road conditions, transportation availability, and geographic barriers, security. Conflict-related Factors; displacement status whether the

mother or household has been displaced due to conflict (yes/no).Perceived security a qualitative measure based on survey responses regarding feelings of safety when accessing health services. Loss, damage of property and nearby persons who care for them.

Data collection methods: Structured questionnaires interviewer administered to expectant mothers to gather data on ANC usage, socio-economic status, and perceived barriers. Geographic positioning Systems (GPS) data were collected through ODK tool using mobile tablets and X-Y coordinate of the household were recorded

### Response:

- Ethical considerations part: Ethical approval and consent to participate part should follow the Helsinki declaration of human participants while you are writing ethics.

**Response:** In conducting our research with vulnerable populations in conflict zones, we prioritize several ethical considerations to ensure the integrity and safety of the study. Informed consent is fundamental; we ensure that participants fully understand the study's purpose, risks, and benefits by simplifying the language to Tigrigna and providing culturally relevant information. Safety and security are paramount, so we assess potential risks associated with participation and implement measures to mitigate them, safeguarding participants, researchers, and the community. We also maintain confidentiality by protecting participants' identities and personal information through anonymization techniques and secure data storage, particularly in sensitive contexts. Our approach is guided by cultural sensitivity, recognizing and respecting local norms and values, and actively engaging with communities to ensure our methods are appropriate. We strive for beneficence and non-maleficence, aiming to maximize benefits while minimizing harm, considering how our research outcomes can positively impact the community. Community engagement is crucial; we involve local stakeholders and organizations, such as health facility workers, in the data collection process to enhance the relevance and acceptance of our research. Additionally, we plan for post-study benefits, ensuring that findings are shared with participants and the community so they can directly benefit from the results. Finally, we have obtained approval from an ethics review board, acknowledging the unique challenges posed by conflict situations and reinforcing our commitment to ethical research practices.

### Strengths and Limitations Strengths:

Spatial modeling offers significant strengths in studying antenatal care interruption, particularly through its ability to provide clear visual representations of geographic patterns. This clarity helps stakeholders identify areas with high rates of interruption, facilitating a better understanding of the spatial dynamics of healthcare access.

Furthermore, these models allow researchers to incorporate a range of socio-economic, environmental, and conflict-related variables, leading to a comprehensive understanding of the factors influencing antenatal care. By pinpointing hotspots, health authorities can strategically allocate resources and design targeted interventions, enhancing the potential for improved access and outcomes in affected regions. Additionally, spatial modeling enables the tracking of changes over time, revealing trends related to ongoing conflicts or recovery efforts, which is crucial for understanding the dynamics of healthcare access in fluctuating environments. The insights gained from these models can also inform policymakers about the specific needs of affected populations, guiding the development of effective health service delivery strategies.

### Limitations:

However, spatial modeling also faces notable limitations. In war-torn regions, the quality and availability of data can be compromised, leading to incomplete or biased datasets that undermine the reliability of the models. Complex health outcomes are influenced by multiple interdependent factors that may not be fully captured, limiting the explanatory power of the models. Moreover, the assumptions and simplifications inherent in spatial modeling can oversimplify the intricate realities of healthcare access, potentially resulting in misinterpretations of the data. Rapid changes in conflict situations may outperform the data used in models, diminishing their relevance over time. Lastly, findings derived from specific regions may not be generalizable to other contexts, which restricts the broader applicability of results and may hinder the development of universal strategies for improving antenatal care access.

- In results part, good to narrate in this way. However, the study should show new insights and should enhance practical relevance while clarifying the scientific concept.

**Response** :tried in its section thank you In Discussion part:

- Please do not repeat result (Numbers). Rather the implication of each association should be clearly explained.

**Response:** made at the document thank you.

- Under the discussion section, you have to start a discussion by summarizing the main findings.

**Response**: Great okay tried at the discussion.

- Possible scientific justification is required for each association whether it agrees or not with other studies.

**Response**: just tried at each but there is limited studies to compare and contrast. Good luck!

Reviewer #2: The research project's title is scientifically sound, and I believe it will significantly contribute to the body of knowledge in maternal health. However, the document is not written in standard English and requires revision. Additionally, some areas need substantial changes and improvements. All comments and suggestions are tracked changes.

**Response**: tried to incorporate your comments thank you.

Comment # 1 Introduction: Explain these new Guidelines. It does not make sense to readers.

**Response:** A N C i n t e r r u p t i o n i s d e f i n e d a s i f m o t h e r d i d n o t f o l l o w A N C

v i s i t a s o f the new World Health Organization (WHO) recommendations, pregnant mothers should attend at least eight antenatal care (ANC) visits throughout their pregnancy.

Comment #2 Materials and methods: Please clarify whether this research utilised secondary data from the "Evaluating" survey, "Cost-Effectiveness in Post-Conflict Settings in Tigray, Ethiopia," or relied on primary data.

**Response**: it is not actually secondary data it is part of the integrated large survey that contains maternal child health services, Social and behavioral derivers of COVID 19 and effectiveness of integrated health services. I mean the data was collected in integrated way.

Comment#3 Result: Please specify the model's fitness and describe the methods employed to assess multicollinearity.

**Response**: model fit improved substantially with the inclusion of the spatial term, as reflected by an increase in log-likelihood from -1200 to -1050 and a decrease in AIC from 2410 to 2110. The

pseudo R-squared increased from 0.55 to 0.72, demonstrating better explanatory power. Multicollinearity diagnostics showed all Variance Inflation Factors (VIFs) below 3, indicating no serious multicollinearity issues among predictors.

Comment #4 Result: Indicate the variables with a P-value of less than 0.05 or significant. Response; Thanks I incorporate it in the document

Comment #5 Discussion: What is the data from the study conducted in Syria?

**Response:** The study in Syria showed negative correlation between bombardments and consultations and ANC visits was reported in the RR was 0.95 (95% CI 0.94–0.97) and 0.95 (95% CI 0.93–0.98), respectively.

Comment # 6 Discussion: How can you compare the distance to health facilities with the impact of conflict and war on the interruption of antenatal care services?

**Response:** the distance was compared using traveling time taken to reach health facilities, though the distance is the same before and after the war because of the many check points, security, blockage , presence of active war and fear of travel alone all together the time needed to reach health facility is different even if the distance is same.

Comment # 7 Discussion: Justify why this particular zone has higher odds of ANC interruption. Compare and contrast with similar findings.

**Response**: Actually I could not get exact zone versus zone variation but I have tried to compare some literature which have similar events like this

this may be the blockage ,movement from place to place ,severity of war may be higher in this area and also because it is far from Mekelle the capital city of the Tigray region which is about 300kms some of the humanitarian organizations were not reaching this area. This is also similar to studies conducted in Syria where bombardment and distant villages from the centers interrupted ANC service than those who are near the centers (22) . This is consistent with In conflict settings, healthcare access is severely compromised due to the destruction of facilities and evacuation of medical personnel, leading to increased travel times and reduced service availability(31)Conversely, while distance is a critical factor, other elements such as socio- economic status, education, and community support also play significant roles in healthcare access and continuity in conflict-affected regions(32)Also align with Previous studies have consistently highlighted that geographic disparities, often exacerbated by conflict, play a critical role in shaping healthcare utilization. For instance, in conflict-affected regions of Afghanistan,

women in remote or insecure areas faced higher barriers to ANC attendance due to disrupted infrastructure and security concerns, a pattern that resonates with the higher likelihood of ANC discontinuation

Comment #8 Discussion: Correlate these findings with similar studies.

**Response**; thanks just I have tried to correlate with other similar studies in the document thanks Comment # 9 Discussion: You should move this sentence to the recommendation section.

Response: Just I did it thank you

Comment # 10 Discussion: You need to establish a connection between the increased distance to healthcare facilities in war-torn areas and the discontinuation of the ANC program in rural regions. Rural areas often suffer from inadequate infrastructure, particularly in terms of roads, which hinders access to health facilities even in peaceful places. Consider how you can uniquely frame this argument to support your position.

Comment # 11 Discussion: The argument lacks clarity. Does the North-West zone have more rural kebeles compared to the South-West zone? How can you formulate an argument regarding the rural population in this context?

**Response**:yes you right the north west zone may not have different rural kebeles in particular

,this may be the security ,war intensity and remote from Mekelle which may have many humanitarian organizations during the war period ,now I tried to find similar even if not exact comparisons .

Comment # 12 Discussion: Move this paragraph to the recommendations section. Response: Thanks just I did it

Comment # 13 Discussion: You need to provide reference findings. Response :discussed in the document

53. Odusina, E. K., Ahinkorah, B. O., Ameyaw, E. K., Seidu, A.-A., Seidu, A.-A., Budu, E., Zegeye, B., & Yaya, S. (2021). Noncompliance with the WHO’s Recommended Eight Antenatal Care Visits among Pregnant Women in Sub-Saharan Africa: A Multilevel Analysis. BioMed Research International, 2021, 6696829. <https://doi.org/10.1155/2021/6696829>
54. Owen, D. J., Wood, L., & Neilson, J. P. (2004). Antenatal care for women with multiple pregnancies: the Liverpool approach. [Clinical Obstetrics and Gynecology, 47(1), 263–271.](https://doi.org/10.1097/00003081-200403000-00026) <https://doi.org/10.1097/00003081-200403000-00026>

55 Gupta, S., Yamada, G., Mpembeni, R., Frumence, G., Callaghan-Koru, J. A., Stevenson, R., Brandes, N., & Baqui, A. H. (2014). Factors Associated with Four or More Antenatal Care Visits and Its Decline among Pregnant Women in Tanzania between 1999 and 2010. [PLOS ONE, 9(7).](https://doi.org/10.1371/JOURNAL.PONE.0101893) <https://doi.org/10.1371/JOURNAL.PONE.0101893>

56. Tsadik M, Teka H, Gebremichael MW, Gebretnsae H, Yemane A, Fisseha G, Gebregzabher T, Buruh G, Gebresellasie F, Tekie M, Kiros G. Armed conflict and maternal health service utilization in Ethiopia’s Tigray Region: a community-based survey. BMC public health. 2024 Oct 16;24(1):2840.

57 Ziegler, B. R., Kansanga, M. M., Sano, Y., Sano, Y., Kangmennaang, J., Kpienbaareh, D., & Luginaah, I. (2021). Antenatal care and skilled birth in the fragile and conflict-affected situation of Burundi International Journal of Health Planning and Management, 36(4), 1081–1106. https://doi.org/10.1002/HPM.3157

Comment # 14 Discussion: You need to provide reference findings. Response :discussed in the document

58.Gedef, G. M., Girma, B., Tibebu, N. S., Gashaw, A., & Andualem, F. (2023). Antenatal care utilization and determinants in fragile and conflict-affected situations in Sekota zuria district, northern Ethiopia, 2022: a community-based cross-sectional study. Midwifery. <https://doi.org/10.1016/j.midw.2023.103906>

59. Price, J. I., & Bohara, A. K. (2013). Maternal health care amid political unrest: the effect of armed conflict on antenatal care utilization in Nepal. *Health Policy and Planning*, *28*(3), 309–

319. https://doi.org/10.1093/HEAPOL/CZS062

Comment # 15: Add the strengths and limitations of the study.

### Response:

Strength and limitation of the study

Spatial modeling offers significant strengths in studying antenatal care interruption, particularly through its ability to provide clear visual representations of geographic patterns. This clarity

helps stakeholders identify areas with high rates of interruption, facilitating a better understanding of the spatial dynamics of healthcare access. Furthermore, these models allow researchers to incorporate a range of socio-economic, environmental, and conflict-related variables, leading to a comprehensive understanding of the factors influencing antenatal care. By pinpointing hotspots, health authorities can strategically allocate resources and design targeted interventions, enhancing the potential for improved access and outcomes in affected regions.

Additionally, spatial modeling enables the tracking of changes over time, revealing trends related to ongoing conflicts or recovery efforts, which is crucial for understanding the dynamics of healthcare access in fluctuating environments. The insights gained from these models can also inform policymakers about the specific needs of affected populations, guiding the development of effective health service delivery strategies.

However, spatial modeling also faces notable limitations. In war-torn regions, the quality and availability of data can be compromised, leading to incomplete or biased datasets that undermine the reliability of the models. Complex health outcomes are influenced by multiple interdependent factors that may not be fully captured, limiting the explanatory power of the models. Moreover, the assumptions and simplifications inherent in spatial modeling can oversimplify the intricate realities of healthcare access, potentially resulting in misinterpretations of the data. Rapid changes in conflict situations may outperform the data used in models, diminishing their relevance over time. Lastly, findings derived from specific regions may not be generalizable to other contexts, which restricts the broader applicability of results and may hinder the development of universal strategies for improving antenatal care access.

Comment # 16: Declare any conflict of interest.

### Response: thanks for your comments we have added the conflicting interest in the revised manuscript

In the research on antenatal care interruption in Tigray, we maintain a strong commitment to avoiding conflicts of interest. We ensure that our funding sources do not create biases, as we do not have any conflicts with organizations that might have vested interests in the outcomes, such as government agencies or NGOs with specific agendas. Our collaborative relationships with local health authorities and NGOs are structured to prevent any conflicts, as these entities do not

have a stake in the research findings that could influence our work. Additionally, the researchers do not have personal ties to the region or its healthcare systems that could compromise the interpretation of data or reporting results. We are also vigilant against publication bias, ensuring that we do not face conflicts that would pressure us to publish only positive outcomes aligned with funding interests, which could distort the integrity of our research. Ethical considerations are paramount; we adhere to rigorous ethical guidelines to address the unique challenges of working with vulnerable populations in conflict zones, recognizing the dilemmas that may arise. To mitigate potential conflicts, we prioritize transparency by disclosing all funding sources and potential conflicts in our publications. We are committed to an independent review process, welcoming input from independent experts in study design and data analysis to ensure objectivity. Finally, we uphold ethical oversight by obtaining approval from relevant review boards Tigray health research institute (THRI) as mentioned above to safeguard participant welfare, reinforcing our dedication to conducting responsible and ethical research.
