# Supplementary material for: Spatial patterns and predictors of antenatal care interruption in war-torn Tigray, northern Ethiopia: Spatial modelling approach
Source: PLoS One. 2025 Aug 14;20(8):e0328802. doi: 10.1371/journal.pone.0328802 (PMC12352755; doi:10.1371/journal.pone.0328802)
Supplement: Attachment — Submitted filename: Response_to_reviewers_auresp_2.docx [file pone.0328802.s004.docx]

Response to Editors and reviewers

Kahsu Gebrekidan, Ph.D.

Academic Editor

PLOS ONE

Dear Dr. Kahsu Gebrekidan and esteemed reviewer,

I hope this message finds you well. I am writing to submit a revised version of our manuscript titled Spatial Patterns and Predictors of Antenatal Care Interruption in War-Torn Tigray, Northern Ethiopia: Spatial Modeling Approach (PONE-D-25-08979R1: Spatial patterns and predictors of antenatal care interruption in war-torn Tigray, northern Ethiopia: spatial modelling approach) in response to the valuable comments and questions provided by the editor and reviewers.

We sincerely appreciate the reviewers' thorough evaluations and insightful feedback, which have greatly enhanced the quality of our work. Below, I summarize our responses to each of the reviewers' comments, along with an outline of the revisions made in the manuscript. We have attached the revised manuscript, a version with track changes, and our one-on-one responses in the system.

Regards

Assefa Ayalew

Mekelle,Tigray ,Ethiopia .

Thank you so much for reviewing our work

1. One of the comments from our reviewers were to separate conclusion and recommendation, thank you so much accepted and we separated as the reviewer recommendation
2. The other comment was proof reading just we read and read by other co-authors too.

Response to editor

The issues raised by the editor was about referencing thank you so much the following references were replaced by other proper references just the replaced once are in the manuscript .

1. WHO, UNICEF, UNFPA and TheWorld Bank. Trends inMaternalMortality: 2000 to 2017WHO. Geneva:WHO (2019).
2. O’hare BAM, David PS. First do no harm: the impact of recent armed conflict on maternal and child health in Sub-Saharan Africa. J R Soc Med. (2007) 100:564–70. doi: 10.1258/jrsm.100.12.564
3. Akseer N, Wright J, Tasic H, Everett K, Scudder E, Amsalu R, et al. Women, children and adolescents in conflict countries: an assessment of inequalities in intervention coverage and survival BMJ Global Health. (2020) 5:e002214. doi: 10.1136/bmjgh-2019-002214
4. Abay ST, Gebre-Egziabher AG. Status and associated factors of birth registration in selected districts of Tigray region, Ethiopia. BMC international health and human rights. 2020 Dec;20:1-0.
5. Stamidis K, Bologna L, Losey L. CORE Group Polio Project (CGPP) Final Evaluation Report 2017.
6. Asah, M., & Moses, A. L. (2025). The Effects of Armed Conflict on Healthcare in Nigeria – A Scoping Review. International Journal of Research and Scientific Innovation, XI(XV), 883–900. https://doi.org/10.51244/ijrsi.2024.11150067p
7. Ahmadi A. Assessing Participation in Women's Development Projects in Afghanistan 2017
8. Owen, D. J., Wood, L., & Neilson, J. P. (2004). Antenatal care for women with multiple pregnancies: the Liverpool approach. [Clinical Obstetrics and Gynecology, 47(1), 263–271. https://doi.org/10.1097/00003081-200403000-00026](https://doi.org/10.1097/00003081-200403000-00026)
